# Protection and mechanism of action of a novel human respiratory syncytial virus vaccine candidate based on the extracellular domain of small hydrophobic protein

**DOI:** 10.15252/emmm.201404005

**Published:** 2014-10-08

**Authors:** Bert Schepens, Koen Sedeyn, Liesbeth Vande Ginste, Sarah De Baets, Michael Schotsaert, Kenny Roose, Lieselot Houspie, Marc Van Ranst, Brian Gilbert, Nico van Rooijen, Walter Fiers, Pedro Piedra, Xavier Saelens

**Affiliations:** 1 VIB Inflammation Research CenterGhent, Belgium; 2 Department of Biomedical Molecular Biology, Ghent UniversityGhent, Belgium; 3 Laboratory of Clinical Virology, Rega Institute for Medical Research, KU LeuvenLeuven, Belgium; 4 Department of Molecular Virology and Microbiology, Baylor College of MedicineHouston, TX, USA; 5 Department of Molecular Cell Biology, Vrije Universiteit AmsterdamAmsterdam, the Netherlands; 6 Department of Pediatrics, Baylor College of MedicineHouston, TX, USA

**Keywords:** alveolar macrophages, Fcγ receptor, human respiratory syncytial virus, small hydrophobic protein, vaccine

## Abstract

Infections with human respiratory syncytial virus (HRSV) occur globally in all age groups and can have devastating consequences in young infants. We demonstrate that a vaccine based on the extracellular domain (SHe) of the small hydrophobic (SH) protein of HRSV, reduced viral replication in challenged laboratory mice and in cotton rats. We show that this suppression of viral replication can be transferred by serum and depends on a functional IgG receptor compartment with a major contribution of FcγRI and FcγRIII. Using a conditional cell depletion method, we provide evidence that alveolar macrophages are involved in the protection by SHe-specific antibodies. HRSV-infected cells abundantly express SH on the cell surface and are likely the prime target of the humoral immune response elicited by SHe-based vaccination. Finally, natural infection of humans and experimental infection of mice or cotton rats does not induce a strong immune response against HRSV SHe. Using SHe as a vaccine antigen induces immune protection against HRSV by a mechanism that differs from the natural immune response and from other HRSV vaccination strategies explored to date. Hence, HRSV vaccine candidates that aim at inducing protective neutralizing antibodies or T-cell responses could be complemented with a SHe-based antigen to further improve immune protection.

## Introduction

Human respiratory syncytial virus (HRSV) is the most important viral cause of acute lower respiratory tract infections (ALRI) in infants worldwide (Liu *et al*, 2012). About 66,000–199,000 children die every year due to complications caused by HRSV infection (Nair *et al*, 2010). By the age of 2 years, most children have been infected at least once by HRSV (Glezen *et al*, 1986; Hall *et al*, 1991). Although in most children HRSV replication remains restricted to the upper respiratory tract, infection regularly spreads to the lower respiratory tract, causing bronchiolitis. This inflammation of the bronchioles is thought to result from massive HRSV infection of the bronchial and alveolar epithelial cells, resulting in sloughing of these cells and formation of clumps that occlude the small airways of the developing infant lungs (DeVincenzo *et al*, 2005; Welliver *et al*, 2008). In high-risk infants, severe HRSV bronchiolitis can be prevented by prophylactic treatment with palivizumab, a HRSV-neutralizing monoclonal antibody (IMpact-RSV Study Group, 1998). Palivizumab, or its affinity-matured variant motavizumab, has however no therapeutic benefit (Ramilo *et al*, 2014). A Cochrane study concluded that therapeutic treatment with aerosolized ribavirin, a guanosine analogue with antiviral activity against both RNA and DNA viruses, might reduce mortality and days of hospitalization in infants with severe HRSV infection (Ventre & Randolph, 2007). Due to its potential teratogenicity, ribavirin is not generally used to treat HRSV-associated illness. As there is no effective antiviral or anti-inflammatory therapy for HRSV, treatment in hospitals is mainly supportive and includes fluid and oxygen supply, and mechanical ventilation. Besides causing acute bronchiolitis, severe HRSV infections in infants can evoke recurrent wheezing at a later age and correlate with a predisposition to allergic asthma (Sigurs *et al*, 2000; Andreakos, 2012; Blanken *et al*, 2013).

The global burden caused by HRSV infections extends beyond very young children. A study performed by Nair *et al* (2013) estimated that annually, HRSV causes about 33.8 million cases of ALRI and 3.3 million cases of severe ALRI requiring hospitalization in children younger than 5 years. In industrialized countries, deaths due to HRSV ALRI are rare (0.7% of all severe ALRI) and occur almost exclusively in children younger than 1 year. However, in developing countries, fatal HRSV infections are more frequent (2.1% of all severe HRSV cases) and remain frequent at later ages (Nair *et al*, 2013). Furthermore, HRSV is increasingly being recognized as a major pathogen in elderly and immunocompromised adults, and even in previously healthy adults (Hall *et al*, 2001; Falsey *et al*, 2005; Luchsinger *et al*, 2012).

Despite the medical importance of HRSV and decades of intensive research, there is at present no licensed vaccine for this virus. A major obstacle and puzzle facing the development of a vaccine with long-lasting protection is the apparent inability of natural HRSV infections to elicit protective immunity. This is illustrated by the recurrence of HRSV infections in all age groups and the high rate of HRSV infections in infants with maternally derived HRSV-neutralizing antibodies (Henderson *et al*, 1979; Hall *et al*, 2001; Collins & Graham, 2008). Even healthy individuals with high levels of neutralizing serum antibodies can be re-infected, even with the same HRSV strain within, 2–6 months (Hall *et al*, 1991).

Vaccines used to prevent or treat infectious diseases aim at mimicking at least part of the host immune response that accompanies recovery from natural infection. In many cases, this implies the induction of neutralizing antibodies directed against major surface proteins of the pathogen. Likewise, most HRSV vaccines being developed aim at inducing HRSV-neutralizing antibodies directed against either the HRSV attachment protein (G) or the fusion protein (F) (Graham, 2011). This strategy has not yet produced an effective HRSV vaccine, but is reasonably still further explored.

We have explored an alternative, unconventional vaccination strategy to control HRSV infection. Next to the F and G proteins, HRSV also expresses a third membrane protein, the small hydrophobic (SH) protein (Olmsted & Collins, 1989; Collins *et al*, 1990). Although the exact function of SH remains poorly understood, it folds into pentameric cation-selective ion channels that can activate the NLRP3 inflammasome (Carter *et al*, 2010; Gan *et al*, 2012; Triantafilou *et al*, 2013). The importance of these functions remains unknown as recombinant HRSV that lacks SH expression is not attenuated *in vitro* and only slightly attenuated in mice and non-human primates (Bukreyev *et al*, 1997; Whitehead *et al*, 1999). Due to its small size and low abundance on HRSV virions, SH is poorly immunogenic upon natural infections (Connors *et al*, 1991; Akerlind-Stopner *et al*, 1993). We generated immunogens that allowed induction of IgG antibodies directed against the SH ectodomain (SHe) in laboratory mice and cotton rats. These SHe-specific antibodies lacked virus-neutralizing activity, readily bound to the surface of HRSV-infected cells and reduced HRSV replication in these two animal models. Based on selective immune cell depletion studies and passive transfer experiments, we also identified the mechanism of action of this novel HRSV vaccination approach.

## Results

### SHe-specific immunity reduces HRSV replication

**Figure 1:**
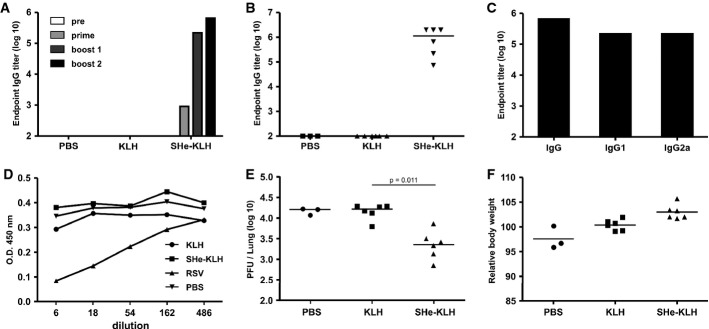
Immunization with SHe conjugate vaccine reduces HRSV replication in BALB/c mice SHe-KLH immunization induces SHe-specific serum IgG antibodies. BALB/c mice were immunized 3 times intraperitoneally with 20 μg KLH or SHe-KLH combined with incomplete Freund's adjuvant or with PBS. Serum was collected one day before the first immunization and 20 days after each immunization.Endpoint IgG titers of pooled sera from each group (*n* = 6, except PBS group: *n* = 3) tested in a SHe peptide ELISA.Individual SHe-specific serum IgG titers obtained 20 days after the second boost immunization. Horizontal bars represent the median.SHe-specific serum IgG, IgG1, and IgG2 endpoint titers in pooled sera after the second boost immunization.SHe-KLH immune serum does not neutralize HRSV *in vitro*. An HRSV A2 *in vitro* neutralization assay using the indicated dilutions of pooled sera, obtained after the second boost vaccination of BALB/c mice immunized as in (A), was performed. RSV: serum from BALB/c mice that were previously infected with HRSV A2. The amount of viral antigen was quantified by ELISA using goat anti-HRSV immune serum. The graph shows the O.D. for each sample.Vaccination with SHe-KLH reduces HRSV A2 replication in the lungs of challenged BALB/c mice. The graph shows the number of PFU per lung for each mouse, sampled 5 days after challenge with 10^6^ PFU of HRSV A2. Horizontal bars depict the median value (Dunn's multiple comparisons test).Challenged SHe-KLH-immunized BALB/c mice do not display weight loss. The graphs are representative for two independent experiments, and horizontal bars depict the mean value.

Experimental or natural infection with HRSV leads to a poor humoral immune response toward the SH ectodomain (Akerlind-Stopner *et al*, 1993; Connors *et al*, 1991 and see below). Therefore, we first improved the immunogenicity of a peptide corresponding to SHe by linking it chemically to keyhole limpet hemocyanin (KLH). The SHe-KLH conjugate was used to immunize BALB/c mice in combination with incomplete Freund's adjuvant (IFA). Using a SHe peptide-based ELISA, we found that immunization with SHe-KLH induced SHe-specific serum IgG1 and IgG2a antibodies and that the levels of IgG antibodies were increased by booster immunizations (Fig 1A–C). In contrast, little or no SHe-specific IgG antibodies were detected in mice infected with live HRSV (Fig 8C; Supplementary Fig S2C).

We then investigated whether SHe-specific serum antibodies from immunized mice could neutralize HRSV infections in an *in vitro* plaque reduction assay. In contrast to sera derived from HRSV A2 infected mice, high-titer SHe immune serum failed to neutralize this virus *in vitro* (Fig 1D). To test whether SHe-based immunization could counteract HRSV infections, mice were challenged with 1 × 10^6^ plaque-forming units (PFU) of HRSV A2. Compared to control vaccinated animals, all SHe-KLH-immunized mice displayed significantly lower pulmonary HRSV titers at 5 days post-infection (Fig 1E). Furthermore, following challenge, SHe-KLH-immunized mice had a slightly higher body weight compared to both control groups (Fig 1F). SHe-specific antibodies were also induced by SHe peptides conjugated to virus-like particles derived from Hepatitis B core (HBc) protein and by SHe linked genetically to recombinant tetrameric and pentameric scaffold proteins, although these responses were less robust than those induced by SHe-KLH (data not shown).

**Figure 2:**
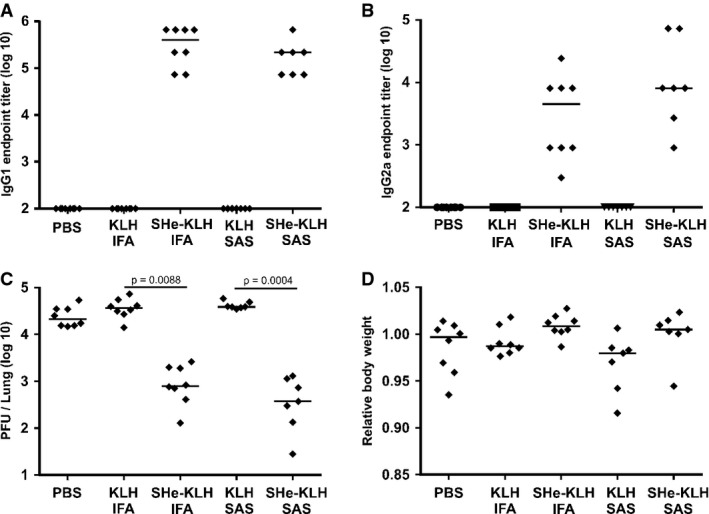
The reduction of HRSV in SHe-KLH-immunized mice is not short living Two groups of 8 BALB/c mice were immunized three times intraperitoneally with KLH or SHe-KLH combined with incomplete Freund's adjuvant (KLH IFA and SHe-KLH IFA). In parallel, two groups of 7 mice were immunized two times intraperitoneally with KLH or SHe-KLH in combination with Sigma Adjuvant System (KLH SAS and SHe-KLH SAS). As a third immunization, the latter two groups received PBS without adjuvant. As negative control, one group of 8 mice was vaccinated with PBS without adjuvant (PBS). All immunizations were performed every 2 weeks. Serum was collected 20 days after each immunization. Six weeks after the last immunization with incomplete Freund's adjuvant and 8 weeks after the last immunization with Sigma Adjuvant System, the mice were challenged with 1 × 10^6^ PFU HRSV A2. Six days after challenge, the lungs were collected to determine the pulmonary HRSV titer by plaque assay.SHe-KLH immunization induces SHe-specific serum IgG1 antibodies. The graph shows the SHe-specific IgG1 serum endpoint titers of each mouse at 3 weeks before viral challenge as determined by SHe peptide ELISA. Horizontal bars indicate the mean IgG1 titers.SHe-KLH immunization induces SHe-specific serum IgG2a antibodies. The graph shows the SHe-specific IgG2a serum endpoint titers of each mouse at 3 weeks before viral challenge as determined by SHe peptide ELISA. Horizontal bars indicate the mean IgG2a titers.Vaccination with SHe-KLH reduces HRSV A2 replication in the lungs of challenged BALB/c mice. The graph shows the number of PFU per lung for each mouse, sampled 6 days after challenge with 1 × 10^6^ PFU of HRSV A2. Horizontal bars represent the median (one-way ANOVA Dunn's multiple comparisons test).Vaccination with SHe-KLH is not associated with enhanced body weight loss upon HRSV infection. The graph shows the relative body weight at day 6 post-infection calculated as the ratio between body weight at day 6 and body weight at day 0. Horizontal bars represent the median.

To investigate whether the reduction of HRSV replication in SHe-KLH-vaccinated mice is short living or long living, BALB/c mice were vaccinated with KLH or SHe-KLH in combination with either IFA or Sigma Adjuvant System (SAS). As a negative control, mice were mock-vaccinated with PBS without adjuvant. Immunizations with IFA were performed three times, whereas immunizations with SAS were performed twice. Figure2A and B show that mice immunized with SHe-KLH with either adjuvant had high levels of SHe-specific serum IgG1 and moderate levels of serum IgG2a at 3 weeks before viral challenge. Six weeks after the last immunization with IFA and 8 weeks after the last immunization with SAS adjuvant, the mice were challenged with 1 × 10^6^ PFU of HRSV A2. At six days post-challenge, all mice that were vaccinated with SHe-KLH had significantly lower lung HRSV titers as compared to KLH- or PBS-vaccinated mice (Fig 2C). Up till 6 days post-infection, no significant differences in body weight were observed, although there was a trend toward somewhat higher relative body weight for SHe-KLH-immunized mice as compared to KLH-immunized mice (Fig 2D). In a separate experiment, HRSV challenge was postponed to eleven weeks after the last immunization with KLH or SHe-KLH in combination with IFA. Supplementary Fig S1 shows that at eleven weeks after the last immunization, all mice had high SHe-specific IgG serum titers that were slightly lower than serum titers at 4 weeks after the last immunization. Supplementary Fig S1C illustrates that also when challenge with 1 × 10^6^ PFU, HRSV A2 is performed 11 weeks after the last immunization, SHe-KLH-vaccinated mice had significantly lower lung HRSV titers as compared to KLH-vaccinated mice. Together, these data indicate that the protection afforded by SHe-based vaccination is relatively long living.

The amino acid sequence of SHe is highly conserved among the group A HRSV but differs substantially from SHe in group B HRSV where it is also sequence conserved (Supplementary Figs S10 and S11) (Collins *et al*, 1990). Consequently, it is likely that vaccination against HRSV B subgroup viruses would require a subgroup-B-matched SHe (SHe-B). Immunization of mice with an SHe-B peptide conjugated to HBc-VLP-induced SHe-B-specific serum IgG that reacted poorly with SHe of subgroup A HRSV (Supplementary Fig S2A–C). These sera however readily bound to the surface of cells infected with HRSV B (Supplementary Fig S2D). To test whether SHe-B immunization affects HRSV B replication in mice, we investigated whether replicating virus could be isolated from the lungs of BALB/c mice infected with a laboratory strain of HRSV B (HRSV B1) or with HRSV B clinical isolates (JX576729, JX576730, JX576731 in Supplementary Fig S11) (Tan *et al*, 2013a,b). Despite several attempts, we could not isolate replicating virus from the lungs of mice that had been infected with any of these HRSV B strains. However, infection of BALB/c mice with clinical HRSV B isolates caused significant body weight loss and pulmonary infiltration of leukocytes (data not shown). Therefore, as a read out for protection against HRSV B, we monitored body weight loss and cellular infiltration of the lungs of mice infected with clinical HRSV B isolate JX576731. BALB/c mice were vaccinated three times with HBc-SHeB, with unconjugated HBc, both in combination with IFA, or were mock vaccinated with PBS (Supplementary Fig S2). Due to the high degree of conservation of the F protein between HRSV A and HRSV B subgroup viruses, HRSV A infections induce HRSV B neutralizing antibodies (White *et al*, 2005). Therefore, prior infection with HRSV A2 was included as a positive control. Three weeks after the last immunization, the mice were challenged with 1.5 × 10^6^ PFU of HRSV B. Seven days after infection, there was a trend toward lower body weight loss, less pulmonary CD8^+^T-cell infiltration, and lower levels of HRSV B-specific RNA in the lungs of HBc-SHeB-vaccinated mice as compared to HBc-vaccinated control mice (Supplementary Fig S2E–G). Prior infection with HRSV A strongly reduced the levels of HRSV B-specific RNA in the lungs but failed to prevent body weight loss and pulmonary cell infiltration. These data suggest that SHe-B based vaccination might also hamper HRSV B replication *in vivo*. To test the potential cross-reactivity between SHe of HRSV A and HRSV B viruses, KLH, SHe-KLH, and HBc-SHeB immune sera were tested in a SHeA and SHeB peptide ELISA. Supplementary Fig S2I–J show that SHeA peptide is bound by SHeA-KLH immune serum and not by HBc-SHeB immune serum. Accordingly, SHeB peptide can only be bound by HBc-SHeB immune serum and not by SHeA-KLH immune serum. Hence to cover both HRSV A and HRSV B viruses, a SHe-based vaccine should contain both the SHeA and SHeB antigen.

### SHe-based vaccination reduces HRSV replication in cotton rats

**Figure 3:**
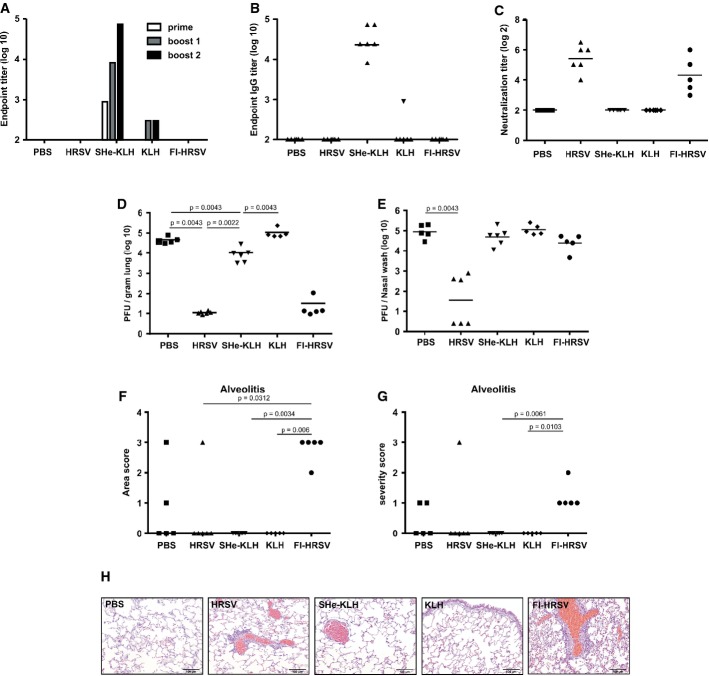
SHe-KLH immunization reduces pulmonary HRSV replication in cotton rats Cotton rats (*n* = 6 per group) were immunized three times intraperitoneally with 20 μg of KLH or SHe-KLH combined with incomplete Freund's adjuvant or with PBS. Additional groups of animals were infected once intranasally with live HRSV or immunized once intramuscularly with formalin-inactivated HRSV (FI-HRSV) at the same time of priming of the other groups. Serum was collected one day before the first immunization and 20 days after each immunization (boost one and two).Endpoint titer of SHe-specific IgG in pooled sera after each immunization.Individual SHe-specific serum IgG endpoint titers, sampled 20 days after the second boost. Horizontal bars represent the mean.SHe-KLH immune cotton rat serum does not neutralize HRSV *in vitro*. The graph shows the HRSV-neutralizing titer in serum isolated 20 days after boost immunization of each cotton rat. Note: One animal that was vaccinated with FI-RSV died before HRSV challenge. Horizontal bars represent the mean.Vaccination with SHe-KLH reduces HRSV replication in the lungs of challenged cotton rats. The graph shows the number of PFU/g of lung tissue for each animal, sampled 5 days post-infection with 2.25 × 10^5^ PFU of HRSV Tracy. Horizontal bars represent the mean (unpaired 2-sided Mann–Whitney *U*-test). Note: One animal in the KLH and one animal in the PBS-vaccinated group died after HRSV challenge.HRSV titers in the upper respiratory tract of challenged cotton rats. The graph shows the number of PFU per nasal wash for each animal, sampled 5 days post-infection with 2.25 × 10^5^ PFU of HRSV Tracy. Horizontal bars represent the mean (unpaired 2-sided Mann–Whitney *U*-test).Alveolitis area score for the lungs of each animal (one-way ANOVA Dunn's multiple comparisons test).Alveolitis severity score for the lungs of each anima (one-way ANOVA Dunn's multiple comparisons test).Representative Micrographs (20×) of H&E-stained lung sections. Scale bars: 100 μm.

Cotton rats are more permissive for HRSV infection than laboratory mice (Byrd & Prince, 1997). Therefore, we also evaluated SHe-based immune protection in this animal model. Cotton rats (six animals per group) were immunized three times intraperitoneally with KLH or SHe-KLH in combination with IFA, or with PBS. Two booster immunizations were given after 3-week intervals. As positive control for protection, one group of cotton rats was infected with HRSV A Tracy (GA1 genotype) at the time of priming of vaccinated animals. Enhancement of disease, characterized by increased alveolitis and alveolar eosinophilia, upon infection with HRSV of formalin-inactivated HRSV (FI-HRSV) vaccine recipients can be recapitulated in cotton rats (Piedra *et al*, 1989; Boukhvalova *et al*, 2009). Because our SHe-based vaccine approach does not mimic natural immunity and does not induce an *in vitro* neutralizing response, we wanted to ascertain that this approach was safe. As a positive control for exacerbation of disease following HRSV A infection, one group of cotton rats was immunized with alum-adjuvanted formalin-inactivated HRSV A Bernett (GA1 genotype). In agreement with our findings in laboratory mice, SHe-KLH elicited high levels of SHe-specific serum IgG antibodies in cotton rats. Also in cotton rats, HRSV A infection did not elicit detectable SHe-specific serum IgG, nor could SHe-specific IgG be detected in sera from cotton rats that had been immunized with FI-HRSV (Fig 3A and B). HRSV infection or immunization with FI-HRSV plus alum-induced protective levels of HRSV A neutralizing serum antibodies (Fig 3C). In contrast, HRSV A neutralizing activity was undetectable in sera from cotton rats immunized with SHe-KLH (Fig 3C). Despite the absence of HRSV A neutralizing antibodies, challenged SHe-immunized cotton rats displayed a tenfold lower pulmonary HRSV A titer as compared to KLH-immunized cotton rats, a difference that was statistically significant (Fig 3D). SHe-KLH-immunized cotton rats also displayed a trend toward reduced HRSV A titers in the nasal tract compared to KLH control animals (Fig 3E). Importantly, and in contrast to challenged FI-HRSV immunized cotton rats, HRSV A challenge of SHe-KLH-immunized animals was not associated with alveolitis or pulmonary infiltration of eosinophils upon HRSV A infection, indicating that vaccination with SHe-KLH in combination with IFA does not exacerbate pulmonary pathology upon infection in cotton rats (Fig 3F–H; Supplementary Fig S3A–J).

### Adoptive transfer of SHe-specific immune serum reduces HRSV replication and associated morbidity in mice

**Figure 4:**
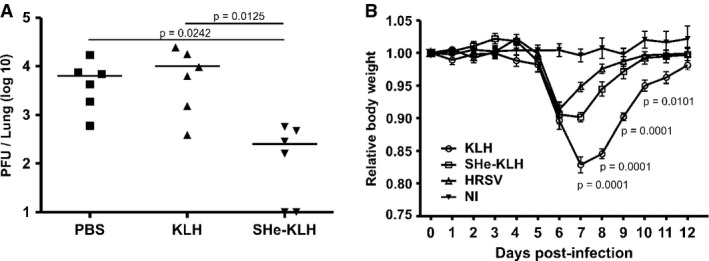
SHe immune serum reduces pulmonary HRSV replication and morbidity One day before and one day after HRSV A2 infection, BALB/c mice were treated intranasally with 35 μl of PBS (PBS), KLH (KLH) or SHe-KLH immune serum (SHe-KLH). Five days after challenge with 5 × 10^5^ PFU of HRSV A2, the lungs were collected to determine HRSV A2 titers. The graph shows the number of HRSV plaques per lung for each mouse 5 days after infection. Horizontal bars represent the mean (one-way ANOVA Dunn's multiple comparisons test).Passively transferred SHe-KLH immune serum reduces body weight loss in HRSV-challenged mice. One day before and one day after infection with 1 × 10^7^ PFU of HRSV A2, BALB/c mice were treated intranasally with 35 μl, KLH, or SHe-KLH immune serum. In addition, one group of mice received serum from mice that had been infected with HRSV. Mice that were not treated and not infected (NI) were used as additional control. The graph represents the average relative (compared to day 0) body weight ± SEM of all mice in each group (*n* = 6, two-way ANOVA Tukey's multiple comparisons test, the indicated *P*-value's concern differences between the KLH and SHe-KLH groups).

Because SHe-based immunization is associated with reduced HRSV replication upon challenge of mice and cotton rats in the absence of demonstrable neutralizing antibodies, we first investigated whether SHe-specific antibodies contributed to protection. We administered serum from mice immunized with PBS, KLH, or SHe-KLH intranasally into naive mice 1 day before and 1 day after HRSV challenge with 5 × 10^5^ PFU of HRSV A2. Five days after infection, the pulmonary HRSV titer was determined. Figure4A shows that lung virus titers were significantly lower in mice that had been treated with SHe-KLH immune serum compared to mice that had received PBS or KLH immune serum. To investigate the dose effect of passive immunization with SHe immune serum, different amounts ranging from 0 to 50 μl of SHe-KLH serum were administered intranasally to mice 1 day before and 1 day after HRSV challenge. All administrated sera were adjusted to a final volume of 50 μl using KLH immune serum. Whereas a dose of 5 μl SHe immune serum could reduce the lung HRSV titer by threefold, 25 and 50 μl SHe immune serum reduced the lung HRSV titer by 11 and 17-fold, respectively, on day 5 after infection (Supplementary Fig S4A and B). None of the mice displayed body weight loss during the experiment (Supplementary Fig S4C and D). To test whether next to intranasal administration also parenteral passive immunization with SHe immune serum could reduce HRSV replication, KLH or SHe-KLH immune serum or HRSV convalescent serum was injected intraperitoneally 16 h before HRSV challenge. Supplementary Fig S4C and D illustrates that also parenteral administration of SHe-KLH immune serum can hamper HRSV replication although to a lesser extent than HRSV convalescent serum.

Although HRSV replicates poorly in mice, challenge of BALB/c mice with a high viral dose is often associated with considerable body weight loss (Graham, 2011). To test whether SHe-specific antibodies can reduce this type of HRSV-induced morbidity, mice were treated with SHe-KLH or control KLH immune serum or with convalescent serum from mice that had been challenged with HRSV. Mice that were not treated and not infected (NI) were used as negative control for HRSV-associated body weight loss. Next, we challenged the animals with a dose of 10^7^ PFU of HRSV A2 and monitored body weight. From day five after infection onwards, all challenged mice started to lose weight (Fig 4B). However, from day 6 after challenge, mice that had received SHe or HRSV immune serum started to recover, whereas mice that had been treated with KLH immune serum continued to lose weight and recovered significantly more slowly (Fig 4B). We conclude that SHe-KLH immune serum reduces HRSV replication and associated body weight loss in mice.

### SHe-antibody-based immune protection depends on Fcγ receptors

**Figure 5:**
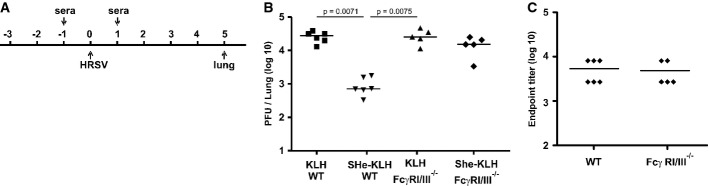
Reduction of HRSV replication by SHe immune serum depends on Fcγ receptors I and/or III Schematic overview of the protocol used to investigate the role of Fcγ receptors in SHe-antibody-mediated reduction of HRSV replication. One day before and one day after HRSV A2 infection, wild-type (WT, 6 mice per group) or Fcγ receptor I and III double knockout (FcγR I/III^−/−^, 5 mice per group) BALB/c mice were treated intranasally with 35 μl of PBS (PBS), KLH (KLH) or SHe-KLH immune serum (SHe-KLH). Five days after challenge with 5 × 10^5^ PFU of HRSV A2, the lungs were collected and HRSV A2 titers were determined by plaque assay.Transfer of SHe-KLH immune serum reduces HRSV replication in wild-type, but not in FcγR I/III^−/−^ mice. The graph shows the number of PFU per lung of each mouse, and horizontal bars represent the means (one-way ANOVA Dunn's multiple comparisons test).SHe-specific IgG levels in lung homogenates of wild-type and FcγR I/III^−/−^ mice are comparable. The graph shows the SHe-specific endpoint IgG titer in lung homogenates prepared 5 days after infection of each mouse treated with SHe-KLH immune serum, with horizontal bars representing the mean. The graphs are representative for two independent experiments.

SHe-specific antibodies failed to neutralize HRSV *in vitro*, yet SHe-specific immune serum reduced HRSV replication *in vivo*, indicating that an alternative mechanism of protection was operating *in vivo*. Antibodies that are directed against virus surface antigens can cooperate with leukocytes to kill or remove virus-infected cells by mechanisms that require Fc receptors on the surface of different types of leukocytes, including NK cells, macrophages, dendritic cells, neutrophils, and eosinophils (Bruhns, 2012; Jiang *et al*, 2011). To test whether Fcγ receptors are involved in the control of HRSV replication mediated by SHe immune serum, we performed adoptive serum transfer experiments in BALB/c mice with a targeted disruption of the activating receptors FcγRI and FcγRIII (Fig 5A) (Hazenbos *et al*, 1996; Meyer *et al*, 1998; Nimmerjahn & Ravetch, 2006). HRSV challenge of wild-type and (FcγRI, FcγRIII)^−/−^ BALB/c mice that had received KLH control immune serum had comparable levels of HRSV in their lungs, indicating that these two mouse strains are equally susceptible to HRSV (Fig 5B) (Bukreyev *et al*, 2008). As expected, SHe immune serum significantly reduced HRSV titers in the lungs of wild-type BALB/c mice. In contrast, adoptively transferred SHe immune serum failed to reduce pulmonary HRSV titers in (FcγRI, FcγRIII)^−/−^ mice. As Fcγ receptors are involved in the pharmacokinetics of IgG, we investigated whether the failure of SHe immune serum to reduce HRSV replication in (FcγRI, FcγRIII)^−/−^ mice might be due to lower levels of SHe-specific IgG antibodies in the lungs (Wang *et al*, 2008). SHe peptide ELISA of lung homogenates revealed that the levels of SHe-specific IgG were comparable in wild-type and (FcγRI, FcγRIII)^−/−^ mice (Fig 5C). We conclude that SH-specific IgG require FcγRI and or FcγRIII to reduce pulmonary HRSV replication in mouse.

### SHe-antibody-based immune protection depends on alveolar macrophages

There are three activating Fcγ receptors in mouse: FcγRI, FcγRIII, and FcγRIV (Nimmerjahn & Ravetch, 2006). These as well as the inhibitory receptor FcγRII are differentially expressed on myeloid and lymphoid cells (Bruhns, 2012). NK cells, for example, can kill infected cells through FcγRIII-mediated antibody-dependent cell-mediated cytotoxicity. As NK cells infiltrate the lungs early during HRSV infection (Hussell & Openshaw, 1998; Moore *et al*, 2008), we tested whether NK cells are involved in SHe-specific IgG-mediated immune protection. To deplete NK cells and prevent NK cell infiltration in the lungs, we treated BALB/c mice with anti-asialoGM1 or rabbit control serum (Supplementary Fig S5A). Treatment of HRSV-infected mice with anti-asialoGM1 antibodies reduced NK cell infiltration in the lungs at 3 days post-infection (Supplementary Fig S5B–D). Anti-asialoGM1 antibody treatment did however not significantly impact the of pulmonary HRSV replication in mice passively immunized with SHe immune serum (Supplementary Fig S5E). These data indicate that NK cells do not play a pivotal role in SHe-specific IgG-mediated control of HRSV replication.

**Figure 6:**
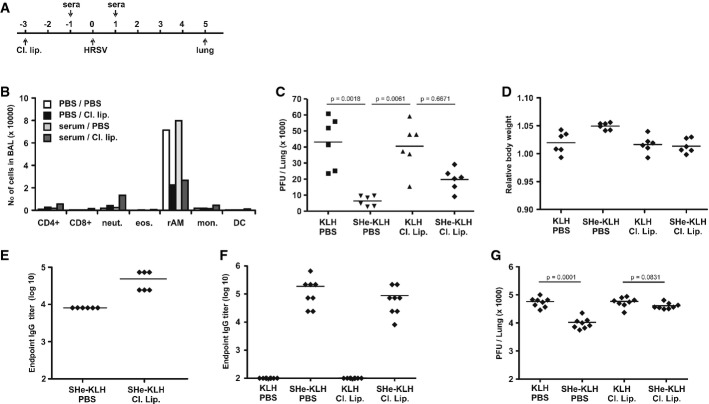
Suppression of HRSV replication by SHe immune serum depends on alveolar macrophages A Schematic overview of the protocol used to investigate the role of alveolar macrophages in SHe-antibody-mediated protection against HRSV. Three days before infection, anesthetized BALB/c mice were treated intranasally with PBS (PBS) or clodronate-loaded liposomes (Cl. Lip.). One day before and one day after HRSV A2 infection, the mice received KLH (KLH) or SHe-KLH immune serum (SHe-KLH) via the intranasal route. On day 5 after challenge with 5 × 10^5^ PFU of HRSV A2, mice were sacrificed and the pulmonary viral load was determined by plaque assay. B Treatment with clodronate-loaded liposomes selectively reduces the number of alveolar macrophages in the lungs. Mice were treated with PBS or clodronate-loaded liposomes 3 days before BAL fluid collection. Two days later, these mice were additionally treated with PBS or KLH immune serum. The number and type of cells in the BAL fluid was determined by flow cytometry. The graph represents the number of CD8^+^ T cells (CD8+), CD4^+^ T cells (CD4+), neutrophils (neut.), eosinophils (eos.), resident alveolar macrophages (rAM), infiltrating monocytes (Mon.), and dendritic cells (DC) in BAL fluid. PBS/PBS: passive transfer of PBS and treatment with PBS; PBS/Cl. lip.: passive transfer of PBS and treatment with clodronate-loaded liposomes; serum/PBS: passive transfer of KLH immune serum and treatment with PBS; Serum/Cl. lip.: passive transfer of KLH immune serum and treatment with clodronate-loaded liposomes. C Treatment of mice with clodronate-loaded liposomes impairs SHe-immune serum-mediated suppression of HRSV replication. The graph shows the number of PFU per lung of each mouse on day 5 after challenge. Horizontal bars represent the means (one-way ANOVA Dunn's multiple comparisons test). The graph is representative for two independent experiments. D Depletion of alveolar macrophages does not affect body weight upon HRSV infection. The graph shows the relative body weight of each animal on day 5 after infection and horizontal bars depict the mean. E Depletion of alveolar macrophages does not decrease the amount of SHe-specific IgG in the lung. The graph shows the SHe-specific IgG endpoint titers in cleared lung homogenates prepared on day 5 after challenge with HRSV A2, as determined by a SHe peptide ELISA. Horizontal bars represent the mean. F, G BALB/c mice (*n* = 16 per group) were immunized three times with KLH or SHe-KLH in combination with incomplete Freund's adjuvant. Immunizations were performed intraperitoneally with 3-week interval. Nineteen days after the last immunization, half of the mice were treated with, respectively, PBS (KLH PBS and SHe-KLH PBS) or clodronate-loaded liposomes (KLH Cl. Lip. and SHe-KLH Cl. Lip.). Three days later, all mice were challenged with 1 × 10^6^ PFU RSV A2. Five days after challenge, the lungs were collected for HRSV titration. (F) SHe-specific IgG endpoint titers of sera collected 2 weeks after the last immunization, as tested by SHe peptide ELISA. Horizontal bars represent the mean. (G) The reduction of HRSV replication in SHe-KLH-vaccinated mice depends on alveolar macrophages. The graph shows the number of PFU per lung for each mouse. Horizontal bars represent the means (one-way ANOVA Tukey's multiple comparisons test).

Alveolar macrophages are the most abundant cell type in the lumen of normal, non-infected lungs. Recently, the presence of FcγRI on the surface of mouse alveolar macrophages has been illustrated by the enhanced binding of FcγRI-specific antibodies as compared to isotype control antibodies (Bruhns, 2012; Guilliams *et al*, 2014). To confirm the expression of FcγRI on the surface of mouse alveolar macrophages, we tested the binding of FcγRI-specific antibodies on the surface of alveolar macrophages isolated from the BALF of wild-type and FcγRI^−/−^ mice by flow cytometry. BALF cells of wild-type mice and FcγRI^−/−^ mice were stained with either IgG1κ anti-mouse FcγRI-specific antibody or an IgG1κ isotype control antibody both in combination with anti-CD11c antibody. Resident alveolar macrophages were detected as autofluorescent (FITC channel), CD11c-positive cells. Supplementary Fig S6 illustrates that in contrast to FcγRI^−/−^ alveolar macrophages, alveolar macrophages from wild-type mice are readily recognized by anti-FcγRI antibodies. These data confirm that alveolar macrophages do indeed express FcγRI. Next, we examined the role of resident alveolar macrophages in SHe-antibody-mediated control of HRSV replication by depleting these cells with clodronate-loaded liposomes (Fig 6A) (Van Rooijen & Sanders, 1994; Pribul *et al*, 2008; El Bakkouri *et al*, 2011). Prior intranasal administration of clodronate-loaded liposomes reduced the number of alveolar macrophages by approximately 70% on the day of infection (Fig 6B). As expected, in HRSV-challenged mice that were treated with PBS, intranasal administration of SHe immune serum significantly reduced the pulmonary HRSV titer as compared to KLH immune serum and was associated with a slightly higher body weight after challenge (Fig 6C and D). In mice that had received control KLH immune serum, depletion of monocytes by clodronate-loaded liposomes did not significantly affect the HRSV pulmonary titer (Fig 6C). In contrast, treatment with clodronate-loaded liposomes partially but significantly restored HRSV replication in the lungs of mice that had been treated with SHe immune serum (Fig 6C). Importantly, pulmonary SHe-specific IgG antibodies were not lower but rather higher in the mice with depleted alveolar macrophages (Fig 6E). This is in line with the role of macrophages in the clearance of IgG (Ordas *et al*, 2012; Wang *et al*, 2008). To investigate the contribution of alveolar macrophages at different time points during HRSV infection, the pervious experiment was repeated but instead of at 5 days post-infection, the lungs were collected at 4 and 6 days post-infection. As expected in mice that were not treated with clodronate-loaded liposomes, intranasal administration of SHe-KLH immune serum significantly reduced the lung HRSV titer at 4 days post-infection by approximately tenfold (Supplementary Fig S7A). In contrast, in mice in which the resident alveolar macrophages were depleted by clodronate-loaded liposomes, there was only a twofold, statistically not significant, reduction in lung HRSV titer in mice that were treated with SHe-KLH immune serum as compared to mice that were treated with KLH immune serum (Supplementary Fig S7A). At 6 days after infection, the reduction (approximately 110-fold) of HRSV replication in the lungs of SHe-KLH immune serum treated mice was more pronounced than at 4 days post-infection (Supplementary Fig S7C). In mice in which the resident alveolar macrophages were depleted by clodronate-loaded liposomes, treatment with SHe-KLH immune serum still reduced the lung viral titer by approximately 15-fold. Supplementary Fig S7B and D illustrate that the reduced effect of SHe immune serum in mice with depleted alveolar macrophages is not due to lower SHe-specific IgG titers in the lungs of these mice. These data indicate that at later time points during HRSV infections, other, possibly infiltrating, leukocytes aid at reducing HRSV replication in the lungs.

Because the intranasal route of serum administration might affect the mechanism by which SHe-specific antibodies hamper HRSV replication in the lungs, the role of alveolar macrophages was also investigated when SHe immune serum was administered parentally. Supplementary Fig S8A and B illustrate that depletion of alveolar macrophages abrogated the reduction of HRSV replication in mice that were treated with SHe immune serum by intraperitoneal injection. To further investigate the role of alveolar macrophages, we tested whether depletion of alveolar macrophages also impairs protection afforded by active SHe vaccination. Mice were vaccinated with KLH or SHe-KLH in combination with IFA. Nineteen days after the last immunization, half of the mice were treated with PBS or clodronate-loaded liposomes. Three days later, all mice were challenged with 1 × 10^6^ PFU RSV A2. Five days after challenge, the lungs were collected for HRSV titration. Figure6F shows that at 3 weeks after the last immunization, mice that were treated with clodronate-loaded liposomes had comparable levels of SHe-specific serum IgG as mock-treated mice. However, in contrast to mock-treated mice, mice that were treated with clodronate liposomes, SHe-KLH vaccination did not reduce HRSV replication in the lungs (Fig 6G). Taken together, these data indicate that SHe-specific antibodies reduce HRSV replication by a mechanism involving Fcγ receptors and alveolar macrophages. However, especially at later time points after infection, also other leukocytes might be involved.

### SHe-specific antibodies bind to HRSV-infected cells but barely to HRSV virions

**Figure 7:**
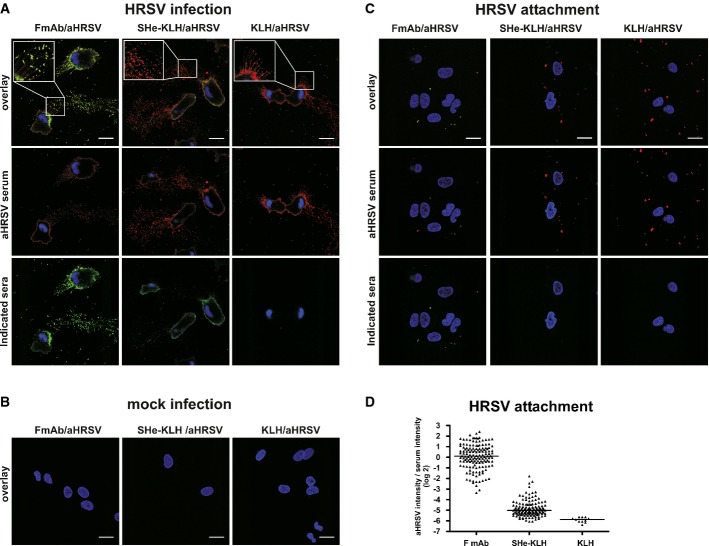
SHe-specific IgG binds to HRSV-infected cells A–C A549 cells were infected overnight with 0.5 MOI HRSV A2 (A), mock-infected (B). In parallel, A549 cells were incubated with 5 MOI HRSV A2 at 4°C for 2 h to allow virus attachment without cell entry (C). After infection or virus attachment, the cells were fixed and stained with SHe-KLH mouse immune serum, KLH mouse immune serum, or a HRSV F-specific mouse monoclonal antibody. Reactivity of mouse IgG was revealed with a green fluorescently labeled secondary antibody. In addition, cells were stained with a polyclonal goat anti-HRSV immune serum, followed by a red fluorescently labeled secondary anti-goat antibody. Nuclei were stained with DAPI (blue color). Confocal images were recorded with a Zeiss SP5 confocal microscope. The upper row of each figure shows the overlay of the red (anti-HRSV), green (indicated serum or antibodies) and the blue (DAPI) signal. The scale bars in the right lower corner of the overlay images indicate 20 μm. D Quantification of virion-associated immunoreactivity of F, SHe-KLH, and KLH mouse IgG on cell-attached HRSV A2 virus particles. This quantification is based on image analysis of multiple micrographs obtained with the attachment and immunostaining experiment shown in (B). The graph shows for each cell-attached virion the ratio of a/b, with ‘a’ being the total pixel intensities of either bound HRSV F-specific monoclonal IgG antibodies (F mAb), SHe-KLH or KLH immune serum IgG and with ‘b’ being the total pixel intensities of bound goat anti-HRSV IgG antibodies. The virions were identified as anti-RSV IgG-positive regions. Multiples images were used for this analysis. Horizontal bars represent the median.

Virus-specific antibodies can affect virus replication by multiple ways ranging from direct virus neutralization to engaging Fc receptor-expressing cells that remove IgG-opsonized virus particles or remove and kill infected cells. To discriminate between an Fcγ receptor mechanism targeting HRSV virions or infected cells, we tested to what extent SHe-specific IgG antibodies in SHe immune serum can bind HRSV virions or HRSV-infected cells. This was investigated by an immunostaining experiment in which the binding of SHe-specific IgG antibodies present in SHe-KLH immune serum to the surface of HRSV A2-infected A549 cells was compared to binding to HRSV A2 virions attached to the surface of A549 cells at 4°C. Because the F protein is readily accessible at the surface of infected cells and virions, a HRSV F-specific IgG monoclonal antibody was used as positive control (Magro *et al*, 2010). Polyclonal anti-HRSV goat immune serum was used to identify the HRSV-infected cells and HRSV virions. Polyclonal anti-HRSV serum recognized the surface of the infected cells, including the long filaments and especially the sprouts of these filaments (Fig 7A and B). Reactivity of the F protein-specific monoclonal antibody was largely confined to the cell surface and filament tips. Since these F-rich tips are of similar size and have an F protein staining pattern that is similar to HRSV virions that are attached to the target cells (Fig 7C), it is likely that these sprouts correspond to budding virions. Similar to the F-specific monoclonal antibody and polyclonal anti-HRSV immune serum, SHe immune serum readily recognized the surface of HRSV-infected cells. However, in contrast to the F-specific monoclonal antibody, SHe immune serum barely bound to the filament tips (Fig 7A and D). In a second approach, performed in parallel, we studied the binding of SHe-specific antibodies to HRSV virions that were attached to but not yet fused with A549 cells (Schepens *et al*, 2011). HRSV virions were recognized by both polyclonal anti-HRSV serum and the F protein-specific monoclonal antibody (Fig 7C and D). More specifically, all virions that were stained by the polyclonal HRSV serum were also recognized by the monoclonal anti-F protein antibody. In contrast, SHe immune serum largely failed to bind to HRSV virions (Fig 7A, C and D). Taken together, these results suggest that SHe immune serum reduces HRSV replication *in vivo* by a mechanism that targets HRSV-infected cells rather than virions.

### Human sera lack high levels of SHe-specific antibodies

**Figure 8:**
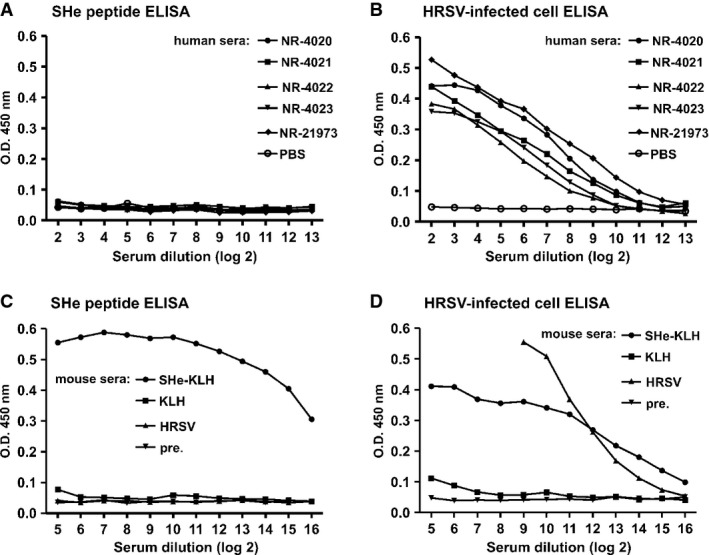
Human sera with high HRSV-neutralizing activity lack high levels of SHe-specific IgG A–D SHe peptide ELISA of human (A) and mouse (C) sera. Human reference sera to HRSV were obtained from the NIH Biodefense and Emerging Infections Research Resources Repository (NIAID, NIH). Antiserum NR-4020: pooled human reference serum; NR-4021: pooled human sera with high HRSV neutralization titer; NR-4022: pooled human sera with intermediate HRSV neutralization titer: NR-4023: pooled human sera with low HRSV neutralization titer; and NR-21973: human reference Ig to HRSV (1% Ig in PBS). The mouse sera represent pooled pre-immune sera (pre.), pooled immune sera from mice immunized three times intraperitoneally with SHe-KLH or KLH in combination with incomplete Freund's adjuvant, and pooled sera of mice infected with HRSV A2 (HRSV). HRSV-specific ELISA of human (B) and mouse (D) sera. The ELISA plates in (B) and (D) were coated with the supernatant of HRSV A2 infected cells and tested with the sera used in (A) and (C).

Many of the current HRSV vaccine approaches aim at inducing HRSV-neutralizing antibodies directed against the F or G protein. However, most adults already have high levels of HRSV-neutralizing serum antibodies. To investigate the levels of SHe-specific IgG in sera of adults, we used SHe peptide ELISA to examine a panel of reference sera with varying levels of HRSV-neutralizing antibodies, as well as purified human serum IgG (Yang *et al*, 2007). In contrast to serum from SHe-KLH-vaccinated mice, sera from HRSV-infected or KLH-vaccinated mice and all human sera lacked detectable SHe-specific IgG antibodies (Fig 8A and C). Still, all tested human sera and sera from HRSV-infected or SHe-KLH-immunized mice readily recognized HRSV proteins in lysates of HRSV-infected cells (Fig 8B and D). These data suggest that HRSV infections in humans do not induce long-living high levels of SHe-specific IgG.

## Discussion

Although the identification of HRSV as an important cause of bronchiolitis in infants was made more than 55 years ago, there is still no licensed vaccine for this important respiratory pathogen (Collins & Graham, 2008). Most attempts to develop such a vaccine aim at inducing neutralizing antibodies directed against the major surface proteins, F and G (Graham, 2011). Inducing neutralizing serum antibodies is a reasonable approach because different reports have illustrated that high levels of such antibodies correlate with reduced disease (Piedra *et al*, 2003; Luchsinger *et al*, 2012; Glezen *et al*, 1986). Moreover, prophylactic passive immunization of high-risk infants with the humanized monoclonal antibody palivizumab, which is F specific and virus neutralizing, reduces the risk of HRSV-associated hospitalization by approximately 50% (IMpact-RSV Study Group, 1998). However, natural HRSV infection does not induce long-lasting immunity. Unlike human influenza, this lack of protection against reinfection occurs without significant antigenic drift (Collins & Graham, 2008). Apart from a recently described neutralizing epitope that is unique to the prefusion conformation of F, the fusion protein of HRSV is highly conserved (McLellan *et al*, 2013). Healthy adults with high levels of neutralizing antibodies in circulation can be experimentally reinfected with HRSV (Hall *et al*, 1991). In addition, some studies even reported a lack of demonstrable correlation between high levels of serum neutralizing antibodies and protection against severe disease caused by HRSV (Friedewald *et al*, 1968; Mills *et al*, 1971; Falsey & Walsh, 1992; Brandenburg *et al*, 1997; Falsey *et al*, 1999; Wright *et al*, 2002). Taken together, it remains to be determined whether vaccination strategies that mimic the host immune response following natural infection with HRSV could provide significant protection.

We developed an alternative vaccination strategy that is not based on natural correlates of protection. In contrast to the major surface proteins, F and G, the SH protein does not elicit high levels of antibodies or contribute to protection upon infection (Connors *et al*, 1991; Akerlind-Stopner *et al*, 1993). SH is a pentameric membrane protein that is not absolutely required for *in vitro* virus growth but presumably modulates the host innate immune response and it is essential for *in vivo* viral fitness (Bukreyev *et al*, 1997; Gan *et al*, 2012; Triantafilou *et al*, 2013).

The inability of HRSV infections to induce high levels of SHe-specific antibodies can be explained by the close proximity of this 24 amino acid long ectodomain to the cell and viral membrane and because of its limited incorporation into budding virions (Rixon *et al*, 2004). We confirmed the findings of Rixon *et al*, which were obtained by immune electron microscopy, by immunostaining HRSV virions attached to the surface of target cells.

In agreement with the weak immunogenicity of the SH ectodomain upon HRSV infection and the weak or absent binding of SHe IgG to HRSV virions, SHe immune sera failed to neutralize HRSV *in vitro*. Passive immunization of mice with heat-inactivated SHe immune serum revealed that SHe-specific antibodies were associated with reduced pulmonary HRSV replication, suggesting that these antibodies are prime mediators of immune protection by this novel SHe-based HRSV vaccination approach. Next to SHe-specific antibodies, SHe-specific T cells might also be involved in SHe-vaccination-mediated reduction of viral replication. In mouse, a CD4^+^ T-cell epitope within SHe, and in humans, a CD8^+^ T cells epitope within the SH protein have been reported (Nicholas *et al*, 1989; Cherrie *et al*, 1992). However, we could not detect SHe-specific T cells in the spleens of HRSV-infected mice by ELISPOT or by intracellular cytokine staining, whereas F-specific CD8^+^ T cells were readily detected in the spleens of these HRSV-infected mice (unpublished data). Moreover, depletion of CD8 cells upon HRSV challenge did not abolish the reduction of HRSV in SHe vaccinated mice Supplementary Fig S9).

Apart from direct neutralization of viral particles, antibodies can also combat viral infections by opsonizing viral particles, marking them for uptake by phagocytes or complement-dependent lysis, or by initiating the elimination of infected cells through antibody-dependent cellular cytotoxicity (ADCC), antibody-dependent cellular phagocytosis (ADCP), or complement-dependent cytotoxicity (CDC) (Hogarth & Pietersz, 2012). ADCC and ADCP depend on activating Fc receptors FcγRI, FcγRIII, or FcγRIV in mouse and on FcγRI, FcγRIIA, FcγRIIC, and FcγRIIIB in human (Nimmerjahn & Ravetch, 2006; Bruhns, 2012). These receptors are expressed alone or combined with other activating and/or silencing Fcγ receptors (FcγRIIb in mouse and FcγRIIB in human) on the surface of innate immune effector cells such as monocytes, macrophages, NKs, DCs, neutrophils, eosinophils, and mast cells (Bruhns, 2012). Using (FcγRI, FcγRIII)^−/−^ BALB/c mice, we demonstrated that the reduction of pulmonary HRSV replication mediated by SHe immune serum strongly depends on FcγRI and/or FcγRIII. In addition, conditional depletion of resident alveolar macrophages (rAM) significantly but not completely abrogated the effect of SHe immune serum on HRSV pulmonary titers. The residual antiviral effect of SHe immune serum could be explained by the observation that rAM depletion was not complete (Fig 6B). Alternatively, this may indicate that other Fcγ R expressing cells such as infiltrating monocytes, NK cells, and neutrophils could also be involved, especially at later time points during infection (Supplementary Fig S7).

Therefore, we propose a mechanism in which rAM cooperate with SHe-specific IgG to target infected cells rather than free virions. rAM might also indirectly contribute to SHe immune serum-mediated inhibition of viral replication. Recently, it has become clear that the crosstalk between macrophages and NK cells is important for the host response to pathogens and tumors (Nedvetzki *et al*, 2007; Lapaque *et al*, 2009; Klezovich-Benard *et al*, 2012). Depletion of rAM by clodronate liposomes was shown to impair early NK infiltration and activation in the lungs of HRSV-infected mice (Pribul *et al*, 2008). In this way, macrophages might also promote SHe-specific, antibody-dependent, NK-mediated ADCC of HRSV-infected cells. However, our observation that depletion of NK cells does not impact the activity of SHe immune serum argues against a pivotal role for NK cells.

It is clear that in both experimental models, mice and cotton rats, the impact of SHe protein vaccination on HRSV replication (6 to 70-fold reduction) does not seem to match up to the sterilizing immunity that some of the F protein-based vaccine candidates elicit, including the FI-HRSV vaccine used here in cotton rats. However, one has to take into account that both BALB/c mice and cotton rats are only semi-permissive for HRSV infection, and so high-titer inocula delivered directly to the lower respiratory tract are required for productive infection in these animals. In humans, HRSV infection starts in the upper respiratory tract and viral spread to the lower respiratory tract probably involves multiple rounds of infections. In such a situation, when the number of infected cells increases gradually over time, antibodies that reduce HRSV replication by eliminating HRSV-infected cells could have a greater impact. SHe-specific antibodies were not detected in human reference sera with HRSV-neutralizing activity. Whereas F protein-based vaccination strategies aim at increasing the level of HRSV-neutralizing antibodies, which are already significantly present in sera from adults, a SHe-based vaccination strategy would induce antibodies against an additional immune -protective antigen. Taken into account that SHe-based vaccination does not provide sterile immunity, clinical implementation of an HRSV vaccine based solely on SHe antigen is unlikely. Adding SHe antigen to vaccine candidates that are based on HRSV F and G, in order to induce neutralizing antibodies, or to vaccines that aim at inducing protective T cells, would be an interesting approach to increase immune protection.

There is increasing evidence that non-neutralizing antibodies that can mediate ADCC contribute significantly to protection against HIV infections. The recent RV144 phase III trial for the first time resulted in some level of protection (31% compared to placebo) (Rerks-Ngarm *et al*, 2009). Surprisingly, this protection did not correlate with the induction of neutralizing antibodies but with high levels of non-neutralizing antibodies capable of mediating ADCC (Rerks-Ngarm *et al*, 2009; Bonsignori *et al*, 2012; Haynes *et al*, 2012; Liao *et al*, 2013). Likewise, not the presence of neutralizing antibodies but the presence of high levels of antibodies capable of ADCC in breast milk correlates with protection against vertical transmission of HIV via breastfeeding (Mabuka *et al*, 2012). Moreover, the protective activity of a broadly neutralizing HIV antibody in macaques relies largely on its interaction with Fc receptors (Hessell *et al*, 2007). In addition, ADCC exerted by cross-reactive HA-specific antibodies is associated with cross-protective immunity against the 2009 pandemic H1N1 in macaque monkeys that had been previously infected with seasonal H1N1 (Jegaskanda *et al*, 2013). Moreover, in animal models, protection against influenza infections by antibodies directed against the conserved ectodomain of the influenza M2 protein strongly depends on Fcγ receptors on macrophages (El Bakkouri *et al*, 2011). Next to the non-neutralizing M2e antibodies, also the recently discovered human ‘broadly neutralizing’ antibodies that bind to the conserved stalk of influenza HA require on Fcγ receptors for their protective activity *in vivo* (Corti & Lanzavecchia, 2013; Dilillo *et al*, 2014). These examples indicate that induction of antibodies able to eliminate infected cells by Fc receptor-expressing host cells could be an effective strategy for development of vaccines against intracellular pathogens.

In infants, HRSV pathology is characterized by airway occlusion caused by sloughed HRSV-infected cells (Welliver *et al*, 2008). This feature of HRSV pathology in infants can be mimicked in HRSV-infected mice in which alveolar macrophages were depleted by clodronate liposomes (Reed *et al*, 2008; Welliver *et al*, 2008). This indicates that macrophages might help to avert airway occlusion by clearing HRSV-infected cells that are sloughed off as well as apoptotic leukocytes. Hence, it was suggested that vaccination strategies that stimulate local macrophage function might be particularly effective in infants (Reed *et al*, 2008).

For a vaccine or antibody treatment based on a small antigen, it is crucial that the antigen is highly conserved in time and among strains. Recently, a small number of studies described SH coding sequences or complete HRSV genome sequences of clinical isolates. These studies included isolates collected between 1998 and 2011 in Europe, Brazil, and the USA and belonging to different HRSV A genotypes (GA1, GA2, GA4, GA5, and GA7) (Kumaria *et al*, 2011; Rebuffo-Scheer *et al*, 2011; Lima *et al*, 2012; Tan *et al*, 2013a,b). To evaluate the conservation of the ectodomain of HRSV A subgroup SH, we aligned the SHe sequences from these studies (Supplementary Fig S10 and S11). Of the 135 retrieved sequences, 5 isolates (3.7%) displayed one amino acid substitution compared to the consensus sequence. None of the sequences displayed more than one amino acid substitution. No deletions or insertions were observed. In addition, SHe of the HRSV A laboratory strains RSV A2 (M74568) and RSV long (AY911262), respectively, isolated in 1961 in Australia and in 1956 in the USA, and the RSS-2 (NC001803) line-19 (FJ614813) and HRSV A Tracy used in our cotton rat model, are identical to the consensus amino acid sequence. Importantly, the sequence of SHe from group A HRSV and group B HRSV differs substantially (Chen *et al*, 2000). Therefore, a SHe comprising HRSV vaccine candidate should contain both the SHeA and the SHeB antigen.

In conclusion, SHe-based immunity is not based on natural immunity and entails an ADCC-type or ADCP-type of protective mechanism and therefore can be considered complementary to F-, G- and N-based subunit immunization strategies that are being explored (Graham, 2011; Remot *et al*, 2012). Additional preclinical development of this SHe-based vaccine might benefit from additional evaluation in an animal model that is more permissive for HRSV or demonstration of its efficacy in natural hosts for HRSV-related paramyxoviruses that also encode SH. In addition, we propose that a viable path for further clinical evaluation of SHe would be to include this antigen in other HRSV vaccine candidates that aim at inducing neutralizing antibodies and protective T-cell responses.

## Materials and Methods

### Ethics statement

All mouse experiments described in this study were conducted according to the national (Belgian Law 14/08/1986 and 22/12/2003, Belgian Royal Decree 06/04/2010) and European (EU Directives 2010/63/EU, 86/609/EEG) animal regulations. All experiments on mice were animal protocols approved by the ethics committee of Ghent University (permit number LA1400091, approval ID 2007/027, 2010/025, and 2013/025). All efforts were made to avoid or ameliorate suffering of animals. The cotton rat experiment was performed in strict accordance with the recommendations in the Guide for the Care and Use of Laboratory Animals of the National Institutes of Health. The experimental protocols were approved by the BCM Investigational Animal Care and Use Committee.

### Cells and viruses

Hep-2 cells (ATCC, CCL-23), Vero cells (ATCC, CCL-81), and A549 cells ATCC, CCL-185) were grown at 37°C in the presence of 5% CO_2_ in DMEM supplemented with 10% heat-inactivated fetal calf serum (FCS), 1% penicillin, 1% streptomycin, 2 mM L-glutamine, non-essential amino acids (Invitrogen, Carlsbad, California), and 1 mM sodium pyruvate. HRSV A2 (VR-1540) and HRSV B1 (VR-1580) were obtained from ATCC (ATCC, Rockville). The clinical HRSV B isolates were obtained from Gasthuisberg University Medical Hospital of Leuven, Belgium, and described in Tan *et al* (2013a,b) (JX576729, JX576730, and JX576731, see Supplementary Fig S8). All HRSVs were propagated on sub-confluent Hep-2 cells infected with 0.1–0.01 MOI. Three days after infection, the culture medium was collected and cleared by centrifugation (1,000 × *g*) for 15 min. The supernatant was adjusted to 10% PEG and incubated at 4°C with agitation for 4 h. Subsequently, HRSV was isolated by centrifugation (4,000 × *g*) for 30 min at 4°C. The HRSV pellet was resuspended in HBSS containing 20% sucrose, aliquoted, and stored at −80°C. The infectious units in the HRSV stocks were quantified in duplicate or triplicate on Vero cells by plaque assay.

### Mice

Specific pathogen-free female BALB/c mice at the age of 7–8 weeks were purchased from Charles River, and (FcγRI, FcγRIII)^−/−^ mice (BALB/c genetic background) were originally obtained from Dr. Verbeek and bred in the specific pathogen-free (SPF) animal facility of DMBR (Hazenbos *et al*, 1996). All mice were genotyped by a PCR protocol using genomic DNA. The mice were housed in a SPF temperature-controlled environment with 12-h light/dark cycles and given water and food *ad libitum*. Mice were used at 9 weeks of age after adaptation in the animal room for 1 week.

### Production of SHe–KLH antigen

For chemical conjugation of SHe to keyhole limpet hemocyanin (KLH), we used a SHe peptide variant (CGGGS-NKLSEYNVFHNKTFELPRARVNT) in which the cysteine at position 4 in the natural SHe amino acid sequence was substituted by a serine to prevent conjugation at this position. An N-terminal CGGGS linker was added as a flexible spacer separating SHe from the carrier. KLH-peptide conjugation and isolation was performed using the Imject Maleimide-Activated mcKLH Kit (Thermo Fisher Scientific, Rockford, USA) according to the manufacturer's instructions.

### Production of HBc-SHeB antigen

A peptide corresponding to the SH ectodomain of HRSV B with an N-terminal CGGGS linker and cysteine at position 10 replaced by a serine (CGGGS-NKLSEHKTFSNKTLEQGQMYQINT) was chemically conjugated to Hepatitis B core protein virus-like particles (HBc-SHeB) by using the heterobifunctional crosslinker Sulfo-MBS according to the manufacturer's instructions (Pierce, Rockford, USA). The production and purification of recombinant HBc particles in which a single lysine was inserted in the immune dominant loop between amino acids 76 and 77 to allow chemical conjugation is described in De Filette *et al* (2005).

### Mice immunization and infection

At the age of 9 weeks, the mice were immunized intraperitoneally with 20 μg antigen dissolved in PBS to a volume of 100 μl PBS and mixed with 100 μl incomplete Freund's adjuvant or the Sigma Adjuvant System (Sigma-Aldrich, St. Louis, USA). Immunizations were performed three times at 3-week intervals, unless indicated otherwise. Two weeks after each immunization, blood samples were collected from the lateral tail vein. Blood was left to clot at 37°C for 30 min, and serum was collected by taking the supernatant from two consecutive centrifugations. The serum titers of SHe-specific IgG, IgG1, and IgG2a were determined by peptide ELISA. Three weeks after the final immunization, the mice were sedated with isoflurane and challenged intranasally with 1 × 10^6^ PFU of HRSV or a different dose as specified. Five days after infection, the mice were killed by cervical dislocation. The lungs were collected and homogenized with a Heidolph RZR 2020 homogenizer in 1.0 ml HBSS containing 20% sucrose. The lung homogenates were cleared by centrifugation (1,000 × *g*) for 15 min at 4°C and used to determine the viral titer by plaque assay on Vero cells.

### Immunization and infection of cotton rats

This experiment was performed at Baylor College by Dr. PA. Piedra and Dr. B. Gilbert. Cotton rats (*Sigmoden hispidis*) of both genders were bred and housed in the Baylor College of Medicine (Houston, TX, USA) vivarium in cages covered with barrier filters and given food and water *ad libitum*. Cotton rats were immunized intraperitoneally with PBS or with 100 μg KLH or SHe-KLH dissolved in 100 μl PBS and mixed with 100 μl incomplete Freund's adjuvant. Immunizations were performed three times at 3-week intervals. At the time of the first immunization, one group of cotton rats were infected intranasally with 2.04 × 10^5^ PFU of HRSV Tracy (HEp-2 grown) under light sedation with isoflurane, and one group were immunized in the left tibialis anterior with formalin-inactivated HRSV-Bernett grown in Vero cells (Piedra *et al*, 1989). Blood was collected 3 weeks after each immunization. Twenty-four days after the last immunization, all cotton rats were challenged with 2.25 × 10^5^ PFU of HRSV Tracy (100 μl) under light sedation with isoflurane. Five days after challenge, the cotton rats were euthanized with CO_2_. The left lung was tied off and used for histopathology. The remaining right lobes from the lung were removed, rinsed in sterile water to remove external blood contamination, and weighed. The right lobes were transpleurally lavaged using 3 ml of Iscove's medium with 15% glycerin mixed with 2% FBS-MEM (1:1, v:v) injected at multiple sites to totally inflate the lobes. Lavage fluid was recovered by gently flattening the inflated lobes and stored on ice until titered. For nasal washes of the upper respiratory tract, the jaws were disarticulated. The head was then removed, and 1 ml of Iscove's media with 15% glycerin mixed with 2% FBS-MEM (1:1, v:v) was pushed through each nare (total of 2 ml). The effluent was collected from the posterior opening of the pallet and stored on ice until titered. The virus titer in the lung lavage fluids and nasal washes was determined by plaque assay using HEp-2 cells. Six days after infection with duplicate dilution series of the prepared lung lavage fluids and nasal washes, the viral plaques were stained with 0.1% crystal violet in 10% formalin solution and counted. For histopathology, the left lung was perfused with 10% neutral buffered formalin (for 2 h). Tissues were stored in 10% neutral buffered formalin and sent to the Center for Comparative Medicine, BCM, where the pathologist who assigned the qualitative and quantitative histopathological scores was blinded to the group assignments.

### Adoptive serum transfer experiments

SHe-KLH, KLH, and PBS immune sera were obtained from mice that had been immunized 3 times with 20 μg SHe-KLH or KLH in combination with incomplete Freund's adjuvant (Merck Milipore, Billerica, USA),with PBS or from immunologically naive mice that had been infected with 1 × 10^6^ PFU of HRSV to obtain serum with virus-neutralizing activity. Three weeks after the third immunization, blood was obtained from the orbital sinus of mice anesthetized with an intraperitoneal injection of avertin (2.5% in PBS). Blood was left to clot at 37°C for 30 min, and serum was obtained by taking the supernatant from two consecutive centrifugations. The serum was heat-inactivated by incubation at 56°C for 30 min. In passive immunization experiments, 35 μl of heat-inactivated serum was administered intranasally under isoflurane sedation 24 h before and 24 h after HRSV challenge. Five days after challenge with 10^6^ PFU HRSV, the mice were sacrificed and their lungs were excised to determine viral load.

### ELISA

ELISA was used to determine antibody titers in sera from individual mice, or pooled sera. To determine SHe-specific IgG in mouse, cotton rat, and human sera, microtiter plates (type II F96 MaxiSorp, Nunc) were coated with 0.2 μg SHe peptide (NKLCEYNVFHNKTFELPRARVNT) in 100 μl of 50 mM sodium bicarbonate buffer, pH 9.7, and incubated overnight at 37°C. A peptide corresponding to the influenza M2 ectodomain (SLLTEVETPIRNEWGCRCNDSSD) was used as negative control. Alternatively, microtiter plates were coated at 4°C with supernatant of cultured Vero cells that had been infected with HRSV A2. After washing, the plates were blocked for 1 h with 200 μl of 1% BSA in PBS. After 1-h incubation, the plates were washed again. Unless specified otherwise, a series of threefold dilutions of different mouse, cotton rat, or human serum samples, starting with a 1/100 dilution, were loaded on the coated plates. The following human serum samples were obtained from the NIH Biodefense and Emerging Infections Research Resources Respiratory: NR-40210, human reference antiserum to respiratory syncytial virus; NR-4021: human antiserum to respiratory syncytial virus, high control; NR-4022, human antiserum to respiratory syncytial virus, medium control; NR-4023, human antiserum to respiratory syncytial virus, low control; and NR-21973, human reference immune globulin to respiratory syncytial virus (Yang *et al*, 2007). The bound antibodies were detected with individual or a mixture of peroxidase-labeled antibodies directed against mouse isotypes IgG1, IgG2a, IgG2b, or IgG3 (individually diluted 1/6,000) (Southern Biotechnology Associates, Inc., Birmingham, AL, USA), or against human IgG1, IgG2, IgG3, and IgG4 (individually diluted 1/2,000) (Southern Biotechnology Associates, Inc.), or against cotton rat IgG (diluted 1/1,000) (Immunology Consultants Laboratory Inc., Portland, USA) in PBS + 1% BSA + 0.05% Tween 20. After washing, the microtiter plates were incubated for 5 min with TMB substrate (tetramethylbenzidine, Sigma-Aldrich). The reaction was stopped by adding an equal volume 1 M H_2_SO_4_, and absorbance at 450 nm was measured. Endpoint titers were defined as the highest dilution producing an O.D. value twice that of background (pre-immune serum).

### Plaque assay

Monolayers of Vero cells were infected with 50 μl of serial threefold dilutions of the lung homogenates in a 96-well plate in serum-free OptiMEM medium (Invitrogen) supplemented with penicillin and streptomycin. After 3 h, the medium was removed and the cells were washed twice with PBS. After adding 150 μl of growth medium containing 2% FCS and 0.6% avicel RC-851 (FMC Biopolymers), the cells were incubated for 4–5 days at 37°C. After infection, the cells were washed twice with PBS and subsequently fixed in 2% paraformaldehyde. After overnight fixation at 4°C, the paraformaldehyde solution was removed and the cells were washed twice with PBS. Subsequently, the cells were permeabilized with PBS containing 0.2% Triton X-100 for 5 min and blocked with PBS containing 1% BSA. The viral plaques were stained with a polyclonal goat anti-HRSV serum (AB1128, Chemicon International) (1/4,000). After washing three times with 1% BSA in PBS, the cells were incubated with HRP-conjugated anti-goat IgG antibodies (SC2020, Santa Cruz) for 30 min. Non-binding antibodies were removed by washing four times with PBS containing 1% BSA and 0.01% Triton X-100 and once with PBS. Finally, the plaques were visualized by using TrueBlue peroxidase substrate (KPL, Gaithersburg). The plaques of different dilutions were counted, and for each dilution, the number of PFU per lung (1 ml of lung homogenate) was calculated as the number of plaques present in the dilution × the factor of dilution × 20 (20 = 1,000 μl total supernatant volume/50 μl of supernatant used to infect the first well of the dilution series). The number of PFU/lung was then calculated as the average number of PFU/lung calculated for the different dilutions. As each supernatant of the homogenized lungs was tested in duplicate, the final number of PFU/lung was calculated as the average of these duplicates.

### *In vitro* neutralization using mouse sera

Virus neutralization activity of mouse sera was tested by plaque reduction assay. Mouse sera (not heat inactivated) were diluted in serum-free medium and incubated with approximately 50 PFU HRSV for 30 min at 37°C in a final volume of 50 μl. These samples were then used to infect a confluent cell layer of Vero cells grown in a 96-well plate. After 3 h incubation at 37°C, 150 μl of growth medium containing 2% FCS and 0.6% avicel RC-851 (FMC Biopolymers) was added. Three days after infection, plaque formation was detected by immunostaining with goat anti-HRSV serum and HRP-coupled anti-goat IgG. The stained plaques were visualized by using TrueBlue peroxidase substrate (KPL, Gaithersburg, USA) and counted visually. Alternatively, viral antigen was detected by adding 100 μl TMB substrate. After incubation for a few min, 50 μl of colorized TMB substrate of each sample was mixed with an equal volume of 1 M H_2_SO_4_ in a 96-well plate and absorbance was measured at 450 nm.

### *In vitro* neutralization of cotton rat sera

Tests for serum neutralizing antibodies to HRSV Tracy were performed with HEp-2 cells grown in 96-well microtiter plates. Serial twofold dilutions in duplicates starting at 3 log_2_ were performed to determine the neutralizing antibody (Ab) titer for each sample. The neutralizing antibody titer was defined as the serum dilution resulting in > 50% reduction in viral cytopathic effect (CPE). CPE is determined visually after the wells are fixed with 10% neutral buffered formalin and stained with crystal violet. Neutralizing antibody titers are categorical log numbers and not continuous values. The lowest detectable Nt Ab titer is 2.5 log_2_. Samples with non-detectable neutralizing antibody titers were assigned a value of 2 log_2_.

### QPCR of HRSV B RNA in BALF of infected mice

The relative levels of HRSV B N cDNA were determined by qRT–PCR using primers specific for the HRSV B N RNA (RSVB-N-Fw: 5′-GGCTCCAGAATATAGGCATGATTC-3′ and RSVB-N-Rev 5′-TGGTTATTACAAGAGCAGCTATACACAGT-3′) and an HRSV B N-specific FAM-conjugated nucleotide probe (5′-TATCATCCCACAGTCTG-3′). The relative RNA level calculated as 1/2^*n*^ (*n* is the number of PCR cycles) with the calculated value for the mock-infected mice was set as 1.

### Detection of FcγRI on the surface of resident alveolar macrophages

BALF of 4 wild-type mice and 3 FcγRI^−/−^ mice were prepared and, respectively, pooled. After fixation with 1% PFA for 10 min at 4°C, the BALF cells were washed and blocked with PBS containing 1% BSA and 1 μg/ml anti-CD16/CD32 Fc-block for 1 h. Subsequently, the cells were stained with PE/Texas Red-conjugated anti-CD11c in combination with either Alexa647-conjugated anti-FcγRI antibody or a APC-conjugated mouse IgG1κ isotype control antibody. For unstained and single stain controls, a mixtures of wild-type and FcγRI^−/−^ BALF cells were used. Resident alveolar macrophages were detected as highly autofluroscent (FITC channel), CD11c-positive single cells. The samples were measured on a LSRII flowcytometer and analyzed with Flowjo.

### Depletion of alveolar macrophages and flowcytometric analysis of BALF cells

Liposomes containing dichloromethylene diphosphonate (clodronate) or PBS were prepared as described previously (Van Rooijen & Sanders, 1994; Pribul *et al*, 2008). Fifty microliters of PBS or clodronate-loaded liposomes were administered intranasally to isoflurane sedated BALB/c mice 3 days before HRSV challenge. Depletion of alveolar macrophages (AM) was ascertained by determining the cell content of bronchoalveolar lavage (BAL) isolated 72 h after liposome administration under anesthesia with an intraperitoneal injection of avertin (2.5% in PBS). A 23-gauge cannula was inserted into the trachea, and cells were collected by washing the airway lumen twice with 0.5 ml PBS containing 0.05 mM EDTA. Total numbers of BAL cells were counted by using a Bürker chamber (Marienfeld, Lauda-Königshofen, Germany). Trypan Blue was added to exclude dead cells. BAL immune cell composition was determined on an LSR-II flow cytometer (BD Biosciences) by analyzing cellular autofluorescence and surface expression of CD3ε, CD4, CD8a, CD11b, CCR3, MHC-II, and CD11c, similar to the protocol described in Schepens *et al* (2011). All antibodies were purchased from Pharmingen (BD Biosciences) except for CCR3 (R&D Systems). For challenge experiments, clodronate liposomes were administered 3 days before HRSV challenge. One day before and 1 day after HRSV challenge, 35 μl of heat-inactivated SHe-KLH or KLH mouse immune serum was administered intranasally to mice sedated with isoflurane.

### Immunostaining HRSV-infected cells and cells to which HRSV is attached

On day 1, 7 × 10^3^ A549 cells were seeded on glass cover slips in two different 24-well plates. On day 2, the cells of 1 plate were infected with 1 MOI of HRSV A2. On day 3, the cells of the second plate were washed once with cold PBS and then incubated with cold OptiMEM medium containing either 5 MOI HRSV A2 or no virus for 2 h on ice. Subsequently, all cells were washed twice with medium containing 10% FCS and twice with cold PBS. Next, the cells were fixed with 2% paraformaldehyde in PBS for 30 min on ice. After fixation, the cells were washed twice with PBS and blocked with 1% BSA in PBS. The cells were then stained with goat anti-HRSV serum (diluted 1/2,000 in 1% BSA in PBS) (AB1128, Millipore, Massachusetts, USA) in combination with either an HRSV F-specific mouse monoclonal antibody (diluted 1/2,000 in 1% BSA in PBS) (MAB858-1, Millipore, Massachusetts, USA) or serum (precleared overnight with detached A549 cells and diluted 1/500 in 1% BSA) of KLH or SHe-KLH-immunized mice. After washing three times with 1% BSA in PBS, the bound goat and mouse IgG antibodies were detected with, respectively, Alexa594-conjugated donkey anti-goat IgG and Alexa488-conjugated donkey anti-mouse IgG antibodies (Invitrogen Molecular Probes, Paisley, UK). The nucleus was stained with DAPI nuclear stain. Images were recorded with a Leica TCS SP5 confocal microscope. The infection and attachment samples were recorded with the same settings. Images were processed with Volocity software (Perkin Elmer, Massachusetts, USA). For the quantitative analysis of antibody binding to HRSV virions attached to cells, seven images were used for the samples stained with SHe-KLH serum and the F-specific monoclonal antibody. One image was used for the control sample stained with KLH serum.

## References

[b1] Akerlind-Stopner B, Hu A, Mufson MA, Utter G, Norrby E (1993). Antibody responses of children to the C-terminal peptide of the SH protein of respiratory syncytial virus and the immunological characterization of this protein. J Med Virol.

[b2] Andreakos E (2012). Asthma exacerbations: a molecular dichotomy between antiviral and pro-inflammatory responses revealed. EMBO Mol Med.

[b3] Blanken MO, Rovers MM, Molenaar JM, Winkler-Seinstra PL, Meijer A, Kimpen JL, Bont L (2013). Respiratory syncytial virus and recurrent wheeze in healthy preterm infants. N Engl J Med.

[b4] Bonsignori M, Pollara J, Moody MA, Alpert MD, Chen X, Hwang KK, Gilbert PB, Huang Y, Gurley TC, Kozink DM (2012). Antibody-dependent cellular cytotoxicity-mediating antibodies from an HIV-1 vaccine efficacy trial target multiple epitopes and preferentially use the VH1 gene family. J Virol.

[b5] Boukhvalova MS, Prince GA, Blanco JC (2009). The cotton rat model of respiratory viral infections. Biologicals.

[b6] Brandenburg AH, Groen J, van Steensel-Moll HA, Claas EC, Rothbarth PH, Neijens HJ, Osterhaus AD (1997). Respiratory syncytial virus specific serum antibodies in infants under six months of age: limited serological response upon infection. J Med Virol.

[b7] Bruhns P (2012). Properties of mouse and human IgG receptors and their contribution to disease models. Blood.

[b8] Bukreyev A, Whitehead SS, Murphy BR, Collins PL (1997). Recombinant respiratory syncytial virus from which the entire SH gene has been deleted grows efficiently in cell culture and exhibits site-specific attenuation in the respiratory tract of the mouse. J Virol.

[b9] Bukreyev A, Yang L, Fricke J, Cheng L, Ward JM, Murphy BR, Collins PL (2008). The secreted form of respiratory syncytial virus G glycoprotein helps the virus evade antibody-mediated restriction of replication by acting as an antigen decoy and through effects on Fc receptor-bearing leukocytes. J Virol.

[b10] Byrd LG, Prince GA (1997). Animal models of respiratory syncytial virus infection. Clin Infect Dis.

[b11] Carter SD, Dent KC, Atkins E, Foster TL, Verow M, Gorny P, Harris M, Hiscox JA, Ranson NA, Griffin S (2010). Direct visualization of the small hydrophobic protein of human respiratory syncytial virus reveals the structural basis for membrane permeability. FEBS Lett.

[b12] Chen MD, Vazquez M, Buonocore L, Kahn JS (2000). Conservation of the respiratory syncytial virus SH gene. J Infect Dis.

[b13] Cherrie AH, Anderson K, Wertz GW, Openshaw PJ (1992). Human cytotoxic T cells stimulated by antigen on dendritic cells recognize the N, SH, F, M, 22K, and 1b proteins of respiratory syncytial virus. J Virol.

[b14] Collins PL, Graham BS (2008). Viral and host factors in human respiratory syncytial virus pathogenesis. J Virol.

[b15] Collins PL, Olmsted RA, Johnson PR (1990). The small hydrophobic protein of human respiratory syncytial virus: comparison between antigenic subgroups A and B. J Gen Virol.

[b16] Connors M, Collins PL, Firestone CY, Murphy BR (1991). Respiratory syncytial virus (RSV) F, G, M2 (22K), and N proteins each induce resistance to RSV challenge, but resistance induced by M2 and N proteins is relatively short-lived. J Virol.

[b17] Corti D, Lanzavecchia A (2013). Broadly neutralizing antiviral antibodies. Annu Rev Immunol.

[b18] De Filette M, Min Jou W, Birkett A, Lyons K, Schultz B, Tonkyro A, Resch S, Fiers W (2005). Universal influenza A vaccine: optimization of M2-based constructs. Virology.

[b19] DeVincenzo JP, El Saleeby CM, Bush AJ (2005). Respiratory syncytial virus load predicts disease severity in previously healthy infants. J Infect Dis.

[b20] Dilillo DJ, Tan GS, Palese P, Ravetch JV (2014). Broadly neutralizing hemagglutinin stalk-specific antibodies require FcgammaR interactions for protection against influenza virus in vivo. Nat Med.

[b21] El Bakkouri K, Descamps F, De Filette M, Smet A, Festjens E, Birkett A, Van Rooijen N, Verbeek S, Fiers W, Saelens X (2011). Universal vaccine based on ectodomain of matrix protein 2 of influenza A: Fc receptors and alveolar macrophages mediate protection. J Immunol.

[b22] Falsey AR, Hennessey PA, Formica MA, Cox C, Walsh EE (2005). Respiratory syncytial virus infection in elderly and high-risk adults. N Engl J Med.

[b23] Falsey AR, Walsh EE (1992). Humoral immunity to respiratory syncytial virus infection in the elderly. J Med Virol.

[b24] Falsey AR, Walsh EE, Looney RJ, Kolassa JE, Formica MA, Criddle MC, Hall WJ (1999). Comparison of respiratory syncytial virus humoral immunity and response to infection in young and elderly adults. J Med Virol.

[b25] Friedewald WT, Forsyth BR, Smith CB, Gharpure MA, Chanock RM (1968). Low-temperature-grown RS virus in adult volunteers. JAMA.

[b26] Gan SW, Tan E, Lin X, Yu D, Wang J, Tan GM, Vararattanavech A, Yeo CY, Soon CH, Soong TW (2012). The small hydrophobic protein of the human respiratory syncytial virus forms pentameric ion channels. J Biol Chem.

[b27] Glezen WP, Taber LH, Frank AL, Kasel JA (1986). Risk of primary infection and reinfection with respiratory syncytial virus. Am J Dis Child.

[b28] Graham BS (2011). Biological challenges and technological opportunities for respiratory syncytial virus vaccine development. Immunol Rev.

[b30] Guilliams M, Bruhns P, Saeys Y, Hammad H, Lambrecht BN (2014). The function of Fcgamma receptors in dendritic cells and macrophages. Nat Rev Immunol.

[b31] Hall CB, Long CE, Schnabel KC (2001). Respiratory syncytial virus infections in previously healthy working adults. Clin Infect Dis.

[b32] Hall CB, Walsh EE, Long CE, Schnabel KC (1991). Immunity to and frequency of reinfection with respiratory syncytial virus. J Infect Dis.

[b33] Haynes BF, Gilbert PB, McElrath MJ, Zolla-Pazner S, Tomaras GD, Alam SM, Evans DT, Montefiori DC, Karnasuta C, Sutthent R (2012). Immune-correlates analysis of an HIV-1 vaccine efficacy trial. N Engl J Med.

[b34] Hazenbos WL, Gessner JE, Hofhuis FM, Kuipers H, Meyer D, Heijnen IA, Schmidt RE, Sandor M, Capel PJ, Daeron M (1996). Impaired IgG-dependent anaphylaxis and Arthus reaction in Fc gamma RIII (CD16) deficient mice. Immunity.

[b35] Henderson FW, Collier AM, Clyde WA, Denny FW (1979). Respiratory-syncytial-virus infections, reinfections and immunity. A prospective, longitudinal study in young children. N Engl J Med.

[b36] Hessell AJ, Hangartner L, Hunter M, Havenith CE, Beurskens FJ, Bakker JM, Lanigan CM, Landucci G, Forthal DN, Parren PW (2007). Fc receptor but not complement binding is important in antibody protection against HIV. Nature.

[b37] Hogarth PM, Pietersz GA (2012). Fc receptor-targeted therapies for the treatment of inflammation, cancer and beyond. Nat Rev Drug Discov.

[b38] Hussell T, Openshaw PJ (1998). Intracellular IFN-gamma expression in natural killer cells precedes lung CD8+ T cell recruitment during respiratory syncytial virus infection. J Gen Virol.

[b29] IMpact-RSV Study Group (1998). Palivizumab, a humanized respiratory syncytial virus monoclonal antibody, reduces hospitalization from respiratory syncytial virus infection in high-risk infants. The IMpact-RSV Study Group. Pediatrics.

[b39] Jegaskanda S, Weinfurter JT, Friedrich TC, Kent SJ (2013). Antibody-dependent cellular cytotoxicity is associated with control of pandemic H1N1 influenza virus infection of macaques. J Virol.

[b79] Jiang XR, Song A, Bergelson S, Arroll T, Parekh B, May K, Chung S, Strouse R, Mire-Sluis A, Schenerman M (2011). Advances in the assessment and control of the effector functions of therapeutic antibodies. Nat Rev Drug Discov.

[b40] Klezovich-Benard M, Corre JP, Jusforgues-Saklani H, Fiole D, Burjek N, Tournier JN, Goossens PL (2012). Mechanisms of NK cell-macrophage Bacillus anthracis crosstalk: a balance between stimulation by spores and differential disruption by toxins. PLoS Pathog.

[b41] Kumaria R, Iyer LR, Hibberd ML, Simoes EA, Sugrue RJ (2011). Whole genome characterization of non-tissue culture adapted HRSV strains in severely infected children. Virol J.

[b42] Lapaque N, Walzer T, Meresse S, Vivier E, Trowsdale J (2009). Interactions between human NK cells and macrophages in response to Salmonella infection. J Immunol.

[b43] Liao HX, Bonsignori M, Alam SM, McLellan JS, Tomaras GD, Moody MA, Kozink DM, Hwang KK, Chen X, Tsao CY (2013). Vaccine induction of antibodies against a structurally heterogeneous site of immune pressure within HIV-1 envelope protein variable regions 1 and 2. Immunity.

[b44] Lima HN, Botosso VF, Oliveira DB, Campos AC, Leal AL, Silva TS, Bosso PA, Moraes CT, Filho CG, Vieira SE (2012). Molecular epidemiology of the SH (small hydrophobic) gene of human respiratory syncytial virus (HRSV), over 2 consecutive years. Virus Res.

[b45] Liu L, Johnson HL, Cousens S, Perin J, Scott S, Lawn JE, Rudan I, Campbell H, Cibulskis R, Li M (2012). Global, regional, and national causes of child mortality: an updated systematic analysis for 2010 with time trends since 2000. Lancet.

[b46] Luchsinger V, Piedra PA, Ruiz M, Zunino E, Martinez MA, Machado C, Fasce R, Ulloa MT, Fink MC, Lara P (2012). Role of neutralizing antibodies in adults with community-acquired pneumonia by respiratory syncytial virus. Clin Infect Dis.

[b47] Mabuka J, Nduati R, Odem-Davis K, Peterson D, Overbaugh J (2012). HIV-specific antibodies capable of ADCC are common in breastmilk and are associated with reduced risk of transmission in women with high viral loads. PLoS Pathog.

[b48] Magro M, Andreu D, Gomez-Puertas P, Melero JA, Palomo C (2010). Neutralization of human respiratory syncytial virus infectivity by antibodies and low-molecular-weight compounds targeted against the fusion glycoprotein. J Virol.

[b80] McLellan JS, Chen M, Leung S, Graepel KW, Du X, Yang Y, Zhou T, Baxa U, Yasuda E, Beaumont T (2013). Structure of RSV fusion glycoprotein trimer bound to a prefusion-specific neutralizing antibody. Science.

[b49] Meyer D, Schiller C, Westermann J, Izui S, Hazenbos WL, Verbeek JS, Schmidt RE, Gessner JE (1998). FcgammaRIII (CD16)-deficient mice show IgG isotype-dependent protection to experimental autoimmune hemolytic anemia. Blood.

[b50] Mills JT, Van Kirk JE, Wright PF, Chanock RM (1971). Experimental respiratory syncytial virus infection of adults. Possible mechanisms of resistance to infection and illness. J Immunol.

[b51] Moore ML, Chi MH, Goleniewska K, Durbin JE, Peebles RS (2008). Differential regulation of GM1 and asialo-GM1 expression by T cells and natural killer (NK) cells in respiratory syncytial virus infection. Viral Immunol.

[b52] Nair H, Nokes DJ, Gessner BD, Dherani M, Madhi SA, Singleton RJ, O'Brien KL, Roca A, Wright PF, Bruce N (2010). Global burden of acute lower respiratory infections due to respiratory syncytial virus in young children: a systematic review and meta-analysis. Lancet.

[b53] Nair H, Simoes EA, Rudan I, Gessner BD, Azziz-Baumgartner E, Zhang JS, Feikin DR, Mackenzie GA, Moisi JC, Roca A (2013). Global and regional burden of hospital admissions for severe acute lower respiratory infections in young children in 2010: a systematic analysis. Lancet.

[b54] Nedvetzki S, Sowinski S, Eagle RA, Harris J, Vely F, Pende D, Trowsdale J, Vivier E, Gordon S, Davis DM (2007). Reciprocal regulation of human natural killer cells and macrophages associated with distinct immune synapses. Blood.

[b55] Nicholas JA, Levely ME, Mitchell MA, Smith CW (1989). A 16-amino acid peptide of respiratory syncytial virus 1A protein contains two overlapping T cell-stimulating sites distinguishable by class II MHC restriction elements. J Immunol.

[b56] Nimmerjahn F, Ravetch JV (2006). Fcgamma receptors: old friends and new family members. Immunity.

[b57] Olmsted RA, Collins PL (1989). The 1A protein of respiratory syncytial virus is an integral membrane protein present as multiple, structurally distinct species. J Virol.

[b81] Ordas I, Mould DR, Feagan BG, Sandborn WJ (2012). Anti-TNF monoclonal antibodies in inflammatory bowel disease: pharmacokinetics-based dosing paradigms. Clin Pharmacol Ther.

[b58] Piedra PA, Faden HS, Camussi G, Wong DT, Ogra PL (1989). Mechanism of lung injury in cotton rats immunized with formalin-inactivated respiratory syncytial virus. Vaccine.

[b82] Piedra PA, Jewell AM, Cron SG, Atmar RL, Glezen WP (2003). Correlates of immunity to respiratory syncytial virus (RSV) associated-hospitalization: establishment of minimum protective threshold levels of serum neutralizing antibodies. Vaccine.

[b59] Pribul PK, Harker J, Wang B, Wang H, Tregoning JS, Schwarze J, Openshaw PJ (2008). Alveolar macrophages are a major determinant of early responses to viral lung infection but do not influence subsequent disease development. J Virol.

[b60] Ramilo O, Lagos R, Saez-Llorens X, Suzich J, Wang CK, Jensen KM, Harris BS, Losonsky GA, Griffin MP (2014). Motavizumab treatment of infants hospitalized with respiratory syncytial virus infection does not decrease viral load or severity of illness. Pediatr Infect Dis J.

[b61] Rebuffo-Scheer C, Bose M, He J, Khaja S, Ulatowski M, Beck ET, Fan J, Kumar S, Nelson MI, Henrickson KJ (2011). Whole genome sequencing and evolutionary analysis of human respiratory syncytial virus A and B from Milwaukee, WI 1998-2010. PLoS One.

[b62] Reed JL, Brewah YA, Delaney T, Welliver T, Burwell T, Benjamin E, Kuta E, Kozhich A, McKinney L, Suzich J (2008). Macrophage impairment underlies airway occlusion in primary respiratory syncytial virus bronchiolitis. J Infect Dis.

[b63] Remot A, Roux X, Dubuquoy C, Fix J, Bouet S, Moudjou M, Eleouet JF, Riffault S, Petit-Camurdan A (2012). Nucleoprotein nanostructures combined with adjuvants adapted to the neonatal immune context: a candidate mucosal RSV vaccine. PLoS One.

[b64] Rerks-Ngarm S, Pitisuttithum P, Nitayaphan S, Kaewkungwal J, Chiu J, Paris R, Premsri N, Namwat C, de Souza M, Adams E (2009). Vaccination with ALVAC and AIDSVAX to prevent HIV-1 infection in Thailand. N Engl J Med.

[b65] Rixon HW, Brown G, Aitken J, McDonald T, Graham S, Sugrue RJ (2004). The small hydrophobic (SH) protein accumulates within lipid-raft structures of the Golgi complex during respiratory syncytial virus infection. J Gen Virol.

[b66] Schepens B, Ibanez LI, De Baets S, Hultberg A, Bogaert P, De Bleser P, Vervalle F, Verrips T, Melero J, Vandevelde W (2011). Nanobodies(R) specific for respiratory syncytial virus fusion protein protect against infection by inhibition of fusion. J Infect Dis.

[b67] Sigurs N, Bjarnason R, Sigurbergsson F, Kjellman B (2000). Respiratory syncytial virus bronchiolitis in infancy is an important risk factor for asthma and allergy at age 7. Am J Respir Crit Care Med.

[b68] Tan L, Coenjaerts FE, Houspie L, Viveen MC, van Bleek GM, Wiertz EJ, Martin DP, Lemey P (2013a). The comparative genomics of human respiratory syncytial virus subgroups A and B: genetic variability and molecular evolutionary dynamics. J Virol.

[b69] Tan L, Lemey P, Houspie L, Viveen MC, Jansen NJ, van Loon AM, Wiertz E, van Bleek GM, Martin DP, Coenjaerts FE (2013b). Genetic variability among complete human respiratory syncytial virus subgroup A genomes: bridging molecular evolutionary dynamics and epidemiology. PLoS One.

[b70] Triantafilou K, Kar S, Vakakis E, Kotecha S, Triantafilou M (2013). Human respiratory syncytial virus viroporin SH: a viral recognition pathway used by the host to signal inflammasome activation. Thorax.

[b71] Van Rooijen N, Sanders A (1994). Liposome mediated depletion of macrophages: mechanism of action, preparation of liposomes and applications. J Immunol Methods.

[b72] Ventre K, Randolph AG (2007). Ribavirin for respiratory syncytial virus infection of the lower respiratory tract in infants and young children. Cochrane Database Syst Rev.

[b73] Wang W, Wang EQ, Balthasar JP (2008). Monoclonal antibody pharmacokinetics and pharmacodynamics. Clin Pharmacol Ther.

[b74] Welliver TP, Reed JL, Welliver RC (2008). Respiratory syncytial virus and influenza virus infections: observations from tissues of fatal infant cases. Pediatr Infect Dis J.

[b75] White LJ, Waris M, Cane PA, Nokes DJ, Medley GF (2005). The transmission dynamics of groups A and B human respiratory syncytial virus (hRSV) in England & Wales and Finland: seasonality and cross-protection. Epidemiol Infect.

[b76] Whitehead SS, Hill MG, Firestone CY, St Claire M, Elkins WR, Murphy BR, Collins PL (1999). Replacement of the F and G proteins of respiratory syncytial virus (RSV) subgroup A with those of subgroup B generates chimeric live attenuated RSV subgroup B vaccine candidates. J Virol.

[b77] Wright PF, Gruber WC, Peters M, Reed G, Zhu Y, Robinson F, Coleman-Dockery S, Graham BS (2002). Illness severity, viral shedding, and antibody responses in infants hospitalized with bronchiolitis caused by respiratory syncytial virus. J Infect Dis.

[b78] Yang DP, Zielinska E, Quiroz J, Madore D, Rappaport R (2007). Preparation of a respiratory syncytial virus human reference serum for use in the quantitation of neutralization antibody. Biologicals.

