# Supplementary material for: Protection and mechanism of action of a novel human respiratory syncytial virus vaccine candidate based on the extracellular domain of small hydrophobic protein
Source: EMBO Mol Med. 2014 Oct 8;6(11):1436–54. doi: 10.15252/emmm.201404005 (PMC4237470; doi:10.15252/emmm.201404005)
Supplement: Supplementary file 1 [file emmm0006-1436-sd1.pdf]

Protection and mechanism of action of a novel human Respiratory Syncytial Virus vaccine candidate based on the extracellular domain of Small Hydrophobic protein

Bert Schepens, Koen Sedeyn, Liesbeth Vande Ginste, Sarah De Baets, Michael Schotsaert, Kenny Roose, Lieselot Houspie, Marc Van Ranst ,Brian Gilbert, Nico van Rooijen, Walter Fiers, Pedro Piedra, Xavier Saelens

Corresponding author: Xavier Saelens, Ghent University and VIB

Review timeline:

Submission date:	24 February 2014
Editorial Decision:	27 March 2014
Revision received:	24 July 2014
Editorial Decision:	26 August 2014
Revision received:	08 September 2014
Accepted:	09 September 2014

Transaction Report:

(Note: With the exception of the correction of typographical or spelling errors that could be a source of ambiguity, letters and reports are not edited. The original formatting of letters and referee reports may not be reflected in this compilation.)

Editor: Céline Carret

1st Editorial Decision 27 March 2014

Thank you for the submission of your manuscript to EMBO Molecular Medicine. We have now heard back from the three referees whom we asked to evaluate your manuscript. Although the referees find the study to be of potential interest, they do raise a number of concerns that need to be addressed in a major revision of your work.

As you will see from the enclosed reports, both referees 1 and 3 have similar requests for inclusion regarding the vaccine strategy and protection induced. Referee 2 is more concerned about the alveolar macrophage response and suggests performing a time course experiment to strengthen the data. In addition, all referees have further questions and suggestions to improve clarity and conclusiveness and we would like to strongly encourage you to follow their advice.

Should you be able to address the raised concerns with additional experiments where appropriate, we would be willing to consider a revised manuscript.

Please note that it is EMBO Molecular Medicine policy to allow a single round of revision in order to avoid the delayed publication of research findings. Consequently, acceptance or rejection of the manuscript will depend on the completeness of your responses included in the next version of the manuscript.

EMBO Molecular Medicine has a "scooping protection" policy, whereby similar findings that are published by others during review or revision are not a criterion for rejection. Should you decide to submit a revised version, I do ask that you get in touch after three months if you have not completed it, to update us on the status.

Please also contact us as soon as possible if similar work is published elsewhere. If other work is published we may not be able to extend the revision period beyond three months.

I look forward to receiving your revised manuscript.

***** Reviewer's comments *****

Referee #1 (Remarks):

The Authors describe an RSV candidate vaccine based on the extracellular domain of the small hydrophobic protein SH (SHe), which is expressed on infected cells, but is not present on the virion. They show that immunization of mice and cotton rats with a SHe peptide conjugated to KLH or VLPs significantly reduces RSV replication in the lungs, but not in the upper airways. They also demonstrate that the protective effect is mediated by serum antibodies and requires alveolar macrophages and FcgRI/III. Finally they show that the SH antigen is expressed on RSV-infected cells and that antibodies to SH are not elicited by natural infection. These findings are interesting since identify a mechanism of immunity that is not elicited by natural infection, but can be readily elicited by immunization. Overall the data are convincing and extend to RSV an approach that has been pioneered by this lab in the field of influenza with the development of an M2e vaccine.

Specific points:

Can the Authors provide data on the level and on the duration of protection?

In the passive transfer experiment the antibodies were delivered intranasally. Would an intravenous injection achieve the same level of protection and show the same dependency on alveolar macrophages?

In Fig 2 and E2 it seems that immunization with KLH can reduce alveolitis, while mice treated with PBS alone show some pathology. What is the basis of this non-specific effect?

Referee #2 (Remarks):

Induction of neutralizing antibodies against the attachment (G) and/or the fusion (F) proteins of respiratory syncytial virus (RSV) is the usual metric by which immunity is measured. The work done by Schepens and colleagues, here, demonstrate that non-neutralizing antibodies induced in in vivo against the small hydrophobic protein (SHe) of RSV poses an alternative approach in designing an RSV vaccine and is additionally important to others in the field interested in developing novel methods in mounting a protective response in vivo. The authors show that antibodies generated against SHe require Fc-receptor engagement - specifically through alveolar macrophage, to clear RSV-infected cells (as SHe antibody tend to recognize infected cells and not viruses).

However, with the current data, there is weak evidence alveolar macrophages play a major role in the clearance of RSV-infected cells. As shown in Figure 5, knock out of about 75% of alveolar macrophages (Figure 5B) only increased pfu/lung by about two-fold (Figure 5C; SHe-KLH PBS vs

SHe-KLH Cl. Lip.). Although the values may be significant, it is hard to believe that this can correlate to the logs differences of lung titers found in the other experiments. Have the authors considered looking at different time points besides 5 days post infection - perhaps the authors should do a time course experiment to see how these alveolar macrophages could affect viral replication. Additionally, the authors should also look how complement may be involved in virus-infected cells as was done by Terajima and colleagues (JVI December 2011, vol. 85, no. 24).

Specific comments/corrections:

1) In the first paragraph of the introduction, the authors mentioned that there is no effective anti-viral or anti-inflammatory therapy for human respiratory syncytial virus. It would be beneficial for the readers if the authors mention a couple of countermeasure currently used in the introduction section (mention of these countermeasure is noted in the discussion section), besides the ones the authors pose - namely, palivizumab (a monoclonal antibody against the fusion protein) and ribavirin (guanosine analogue).

2) It may also be helpful for the readers if the authors also give a brief description (or at least what is known about it) on the role of the small hydrophobic protein in the viral replication cycle.

3) In the last sentence of the first paragraph on page 7, I think the authors were referring to figure 2F to H instead of 3F to H.

4) In the first paragraph of page 8, the authors should also mention that they also treated the mice with ¨¨¨'not immunized (NI)' sera as one of their controls.

5) Figure 5B, y-axis: a better abbreviation of 'number' would be 'No.' rather than 'Nbr'.

6) Page 10; Figure E4: Showing that Fc-gamma-R1-specific antibodies bind to WT macrophages and not the ones found in knock-out mice by immunofluorescence is visually pleasing, however, perhaps using FACs analysis to measure binding to WT vs KO macrophages would be a better way (to show quantitatively) that they are truly not binding to the KO alveolar macrophages.

Referee #3 (Comments on Novelty/Model System):

Reasons for these choices can be found in my comments to authors

Referee #3 (Remarks):

The paper by Schepens et al. explores the performance of a respiratory syncytial virus (RSV) vaccine based on the extracellular domain of the small hydrophobic (SH) protein of human RSV. The authors synthesized a 23 amino acid peptide based on the SH ectodomain sequence of a group A RSV strain and linked it to keyhole limpet hemocyanin (KLH). The SHe-KLH conjugate was used to immunize mice and cotton rats searching for: i) peptide specific antibody responses and ii) protection against a RSV challenge. The results obtained demonstrate the induction of non-neutralizing antibodies specific for the SH ectodomain and some level of protection against a RSV challenge, suggesting a potential use of SH based vaccines in the future. Passive transfer of immune serum and depletion of certain cell types provided evidence that the SHe-KLH vaccine confers protection by non-neutralizing antibodies that act by some kind of antibody-dependent cellular mechanism likely involving alveolar macrophages. The results are clearly presented and generally support the conclusions reached by the authors.
I think that this unconventional SH based vaccine merits the investigation carried out by Schepens et al., but I feel that relevant information is missing in the manuscript to appreciate the vaccine potential. Thus, the authors should provide as much data and/or information as they have about the following main points:
1.- Duration of protection afforded by the SHe-KLH vaccine. There have been reports of RSV vaccines based on the N or M2-1 proteins that induced short lived protection, while vaccines based

on G or F glycoproteins afforded long lasting protection. Is the new SH based vaccine of the first or second type?
2.- Given the sequence differences between the SH ectodomains of RSV group A and group B viruses, what is the degree of cross-protection (if any) afforded by the SHe-KLH vaccine? Is there any cross-reactivity of the antibodies induced by SH from group A with the SH peptide from group B and vice versa?
3.- What are the basis for the dose used in the immunizations? It would be helpful if the authors could provide data or information about dose effects that justify the chosen amount of SHe-KLH conjugate. The same apply to the passive administration of immune serum. Why 35 µl of serum and why intranasal administration? In other words, are the conditions used for this work optimal or we may expect even better performance of the SHe-KLH vaccine?
Other points:
1.- Page 3, line 6: Is Haynes et al., the correct reference here? Please, check.
2.- Given the sequence divergence between the SH ectodomains of group A and group B viruses, I miss clear data/information about the degree of cross-reactivity of the respective antisera against the heterologous peptide and about the degree of cross-protection afforded in challenge studies. I may have missed this information but if so, it was not clear in the paper.
3.- The use of hepatitis B virus like particles is described only in the expanded view part of the manuscript. The generation of these particles and conjugation of the SH peptide should be described in Materials and Methods. Why not using the KLH approach in this case?
4.- The depletion of macrophages was done only in the mice that passively received immune serum. Why not depleting macrophages in mice immunized with SHe-KLH before challenge? This would be more informative about the role of macrophages in the full response afforded by the conjugate and not only by passively administered antibodies.
5.- As acknowledged by the authors, the impact of SHe protein vaccination on RSV replication is lower than the sterilizing immunity afforded by F-protein based vaccines. This is however not easily seen in the Figures, because the way data is represented. For instance, in Figure 2C the difference in PFU/gram lung between HRSV and SHe-KLH groups seems minimal, since all HRSV values are on top of the abscissas' axis. A log representation of these data would be more illustrative. Please, change Figures to offer a clear picture of SHe-KLH performance in comparison with HRSV.
6.- I found the Discussion extremely long and cumbersome. Please, shorten it by avoiding repeating what it was already mentioned and concluded under Results.
7.- The heading of the second Figure E6 should be "Figure E7".
8.- Figure 7D: The sera from RSV infected mice reacted with SHe-KLH but in Figures 2A and 2B the sera of RSV infected mice failed to react with the same conjugate. Please, clarify.
In summary, addition of more information and data will help to illustrate the full potential of the SH based RSV vaccine as well as polishing some of the aspects raised in the comments mentioned above will improve the manuscript clarity.

1st Revision - authors' response 24 July 2014

Referee 1.

Referee #1 (Remarks):

The Authors describe an RSV candidate vaccine based on the extracellular domain of the small hydrophobic protein SH (SHe), which is expressed on infected cells, but is not present on the virion. They show that immunization of mice and cotton rats with a SHe peptide conjugated to KLH or VLPs significantly reduces RSV replication in the lungs, but not in the upper airways. They also demonstrate that the protective effect is mediated by serum antibodies and requires alveolar macrophages and FcgRI/III. Finally they show that the SH antigen is expressed on RSV-infected cells and that antibodies to SH are not elicited by natural infection. These findings are interesting since identify a mechanism of immunity that is not elicited by natural infection, but can be readily elicited by immunization. Overall the data are convincing and extend to RSV an approach that has been pioneered by this lab in the field of influenza with the development of an M2e vaccine.

We are pleased with the appreciation of referee 1 for our work on SHe as an immunogen that can reduce RSV replication in challenged mice and cotton rats.

Specific points:

Can the Authors provide data on the level and on the duration of protection?

We evaluated the level of protection primarily by determining the number of plaque-forming units (PFU) in lung samples of HRSV A2 challenged mice. We consistently noticed a 5 to 50 fold reduction in lung viral load in SHe-KLH immunized mice (Figures 1E, 2C, E1C) or in mice that received SHe-KLH immune serum (Figures 4A, 5B, 6C, 6G, E4A, E4C, E5E, E7A, E7C, E8B, E9D) compared to control immunized animals. Challenge of BALB/c mice was performed with 5 x 10^5, 1 x 10^6 of 1 x 10^7 (PFU of HRSV A2, a commonly used laboratory strain that replicates fairly well in mice. Lung virus titers were determined on day 4 (Figure E7A) but more often on day 5 or day 6 after challenge. We also monitored the body weight of challenged mice. Although body weight loss after challenge was minimal in negative control mice, which is consistent with published data on HRSV-associated morbidity in laboratory mice, SHe-conjugate vaccination prevented body weight loss (Figures 1F, 2D, 4B, 6D, E1D, E2E, E4B and E4D).
Cotton rats were challenged with 2.25 x 10^5 PFU of HRSV Tracy and nasal washes and lung lavages were prepared on day 5 after challenge and used for virus titration by plaque assay. In the cotton rat experiment, we included an FI-RSV immunized group and a group that was infected with HRSV Tracy on day 0 of the experiment. These two groups served as positive controls for vaccine-associated disease enhancement and protection against HRSV, respectively.

We performed two new experiments to evaluate the duration of protection by SHe-based immunization. The results of these experiments are included in the revised version of our manuscript and presented in two new figures: **Figure E1 and Figure 2**.

For the first experiment, we used BALB/c mice from an ongoing immunization experiment that had been set up prior to the receipt of the referee reports and was initially intended to produce immune sera. This experiment comprised two groups of six BALB/c mice that had been immunized by intraperitoneal injection of 20 µg KLH or SHe-KLH adjuvanted with incomplete Freund's adjuvant (IFA). Immunizations were performed 3 times with 3 weeks interval. These animals were challenged 11 weeks (instead of 3 weeks) after the last immunization with 1 x 10^6 PFU HRSV. Six days after challenge, the lungs were collected to determine the pulmonary HRSV titer. Figure E1A and E1B illustrate that at 11 weeks after the last immunization all SHe-KLH immunized mice contained high levels of SHe-specific serum IgG that were only slightly lower than the levels 4 weeks after the last immunization. Importantly, challenged SHe-KLH immunized mice had a significantly, 10-fold lower pulmonary HRSV titer than KLH immunized control mice (Figure E1C). The body weight relative to the day of challenge was comparable for the two groups (Figure E1D).

In a second experiment we used five groups of BALB/c mice. We included an additional negative control group of mice immunized with PBS without adjuvant and we evaluated the performance of an alternative adjuvant: Sigma Adjuvant System (SAS), an oil-in-water adjuvant containing Monophosphoryl Lipid A. Two groups of mice were immunized intraperitoneally with SHe-KLH in combination with IFA or with SAS. As negative control mice were immunized with non-conjugated KLH in combination with the respective adjuvants. Immunizations with PBS, KLH/IFA and SHe-KLH/IFA were performed 3 times with 2 weeks interval. Immunizations with KLH/SAS and SHe-KLH/SAS adjuvant were performed 2 times with 2 weeks interval. As a third "immunization" the latter two groups of mice received PBS without adjuvant. Six weeks after the last immunization (this is 8 weeks after the last immunization with SAS), the mice were challenged with 1 x 10^6 PFU of HRSV A2. As shown in the new Figures 2A and 2B, three weeks after the last immunization most mice had high levels of SHe-specific serum IgG1 and IgG2a titers. Six days after HRSV challenge, the pulmonary HRSV titers of mice that were immunized with SHe-KLH in combination with IFA were significantly lower (about 30-fold) than the viral titer in mice that were immunized with non-conjugated KLH (Figure 2C). Similarly, also mice that were immunized with SHe-KLH combined with SAS had significantly lower pulmonary HRSV titers than the KLH/SAS mice. Although at 6 days post infection no significant bodyweight loss was observed, mice that were vaccinated with SHe-KLH either in combination with IFA or SAS tended to have a somewhat higher relative bodyweight as compared to the KLH control mice (Figure 2D).

In our opinion, the data from these two new experiments indicate that protection by SHe-KLH vaccination is relatively long living in mice.

In the passive transfer experiment the antibodies were delivered intranasally. Would an intravenous injection achieve the same level of protection and show the same dependency on alveolar macrophages?

Passive transfer of antibodies through the intranasal route is less conventional than parenteral administration of antibodies, *i.e.* by needle injection. We used the intranasal route of antiserum delivery because in our experience with influenza virus and HRSV infection of mice, that route of administration provides better protection against these respiratory viruses than intraperitoneal injection (e.g. Schepens et al., JID 2011, p. 1692-16701; Ibañez et al., JID 2011, p. 1063-1072; De Baets et al., JV 2013, p. 3314-3323; Cardoso et al., JV 2014, p. 8278-8296).

We performed two new experiments to assess whether passive transfer of antiserum by injection would also protect and, if so, would also be dependent on alveolar macrophages. However, we used intraperitoneal rather than intravenous injection of antibodies because this route of antiserum/antibody injection is very often used and because it was more convenient for us to perform as compared to intravenous injection.

We first performed a small-scale experiment using three groups of three mice. The mice received 400 µl of KLH or SHe-KLH immune serum, that had been collected three weeks after the last immunization of a series of 3 immunizations performed with 20 µg KLH or SHe-KLH in combination with IFA. Mice in the third group received 400 µl of convalescent serum derived from BALB/c mice that had been infected with HRSV. KLH, SHe-KLH and HRSV convalescent sera were administered by a single intraperitoneal injection 16 hours before challenge with 5×10^5 PFU HRSV A2. Five days after challenge the lungs were collected to determine the pulmonary HRSV titer by plaque assay. Compared to mice that were treated with KLH immune serum, SHe-KLH immune and HRSV convalescence serum recipients had approximately 8-fold and 40-fold lower pulmonary HRSV titers, respectively. From this experiment we conclude that intraperitoneal injection of SHe-KLH immune serum suppresses HRSV replication in challenged mice. The results of this experiment are shown in **panels C and D of the new Figure E4**.

We next determined if protection following intraperitoneal injection of SHe-immune serum is also dependent on alveolar macrophages. Groups of 6 BALB/c mice were treated intranasally with clodronate loaded liposomes (Cl. Lip., two groups) or PBS (PBS, two groups). One day before challenge with 1×10^6 PFU of HRSV, mice received 400 µl of KLH or SHe-KLH immune serum by intraperitoneal injection. Five days after challenge mice were sacrificed and the lungs were collected for virus titration. On day 5 after infection all SHe-KLH immune serum recipient mice had comparable SHe-specific IgG titers in their sera. PBS treated mice, *i.e.* with intact alveolar macrophage compartment, that were passively immunized with SHe-KLH immune serum had significantly lower levels of HRSV in their lungs as compared to KLH immune serum recipients. In contrast, when alveolar macrophages were depleted by clodronate-loaded liposomes the HRSV titer of SHe-KLH serum treated mice did not differ from the HRSV titer of mice treated with KLH serum. These data indicate that alveolar macrophages are involved in the reduction of HRSV replication by SHe immune serum passively administered via intranasal or parenteral (i.p.) administration. These data are included in the revised manuscript as the **new Figure E8**.

In Fig 2 and E2 it seems that immunization with KLH can reduce alveolitis, while mice treated with PBS alone show some pathology. What is the basis of this non-specific effect?

Figures 2 and E2 in our original submission show data derived from an immunization/challenge experiment in cotton rats. In this experiment we also included an FI-HRSV vaccinated group, which served as a positive control group for exacerbation of disease upon HRSV challenge. We wanted to evaluate in the cotton rat model if SHe-based vaccination might or might not promote such an effect.

From the results shown in figure 2 and figure E2 of the original manuscript, it indeed appears as if challenged PBS vaccinated cotton rats display some alveolitis whereas KLH vaccinated cotton rats do not. The question is what the basis is for this “non-specific” effect. In the original manuscript the mean pathological score is displayed as a bar + SEM. Presenting the data in this way, indeed suggests a degree of alveolitis in the PBS control group, which is absent in the KLH (and SHe-KLH) group. We have changed the presentation of the raw data for the alveolitis area and severity score from the cotton rat experiment: instead of showing an average with SEM, we now present the alveolitis data for each individual animal. In this representation it is clear that 2 out of 5 PBS vaccinated cotton rats and 1 out of 6 HRSV vaccinated animal displayed alveolitis, whereas none of the KLH and SHe-KLH vaccinated cotton rats displayed evidence of alveolitis. In contrast, all cotton rats that were vaccinated with FI-HRSV had alveolitis. There is no statistically significant difference in alveolitis scores between PBS vaccinated and KLH vaccinated animals. In contrast, there is a significant difference in alveolitis scores between KLH or SHe-KLH vaccinated animals compared to animals that were vaccinated with FI-HRSV. In the revised manuscript, we have

presented the pathology scores as data points for the individual animals in each group and we have added the statistical analysis (please see **Figure 3F and –G and Figure E3**).

Although not statistically significant the trend towards lower alveolitis in KLH vaccinated animals as compared to PBS vaccinated animals might suggest a "non-specific" effect on pathology. This possible trend towards lower frequency of alveolitis in KLH vaccinated animals was not associated with lower HRSV titers at 5 days post infection (Figure 3D). This is to some extend reminiscent of a report by Walzl et al (J Exp Med. 2000, 6, p.1317-1326). Walzl and colleagues demonstrated that prior infection with influenza could prevent eosinophilia and disease without affecting HRSV replication. They demonstrated that this protection was mediated by influenza specific memory T cells that upon HRSV infection migrate to the lungs. Although the induced influenza specific memory CD8 T cells did not cross-react with HRSV infected cells, these CD8 T cells expressed IFN-γ. As IFN-γ can prevent eosinophilia upon HRSV infection, the "non-specific" protective effect of influenza virus infection might be mediated through IFN-γ produced by lung infiltrating influenza-specific CD8 cells (Hussell et al., Eur. J. Immunol., 1997, 27, p. 3341-3349). As KLH has strong T-cell epitopes and enables cross-presentation, repeated KLH immunization may also have induced a pool of KLH-specific T cells that could partially counteract alveolar eosinophilia (Harris and Markl, Micron. 1999, 30, p. 597-623).

An alternative explanation for the trend towards reduced alveolitis in the challenged KLH-immunized group is the immunologically naïve nature of the cotton rats. Exacerbation of disease by FI-HRSV vaccination was clearest in very young infants. The typically Th2 skewed immune response of naïve infants might thus promote Th2 driven eosinophilia during FI-HRSV associated exacerbation of disease (Graham et al., J Immunol., 1993, 15, p. 2032-2040; Hussell et al., Eur. J. Immunol., 1997, 27, p. 3341-3349). As growing infants experience infections their immune responses become more Th1 oriented (Prescott et al., Lancet, 1999, 16, p. 196-200). Consecutive immunizations with KLH might act as surrogate for such infection in laboratory mice, likewise suppress a Th2 biased immune response and hence alveolar eosinophilia upon HRSV challenge (Walzl et al., J Exp Med. 2000, 6, p.1317-1326).

Referee 2.

Referee #2 (Remarks):

Induction of neutralizing antibodies against the attachment (G) and/or the fusion (F) proteins of respiratory syncytial virus (RSV) is the usual metric by which immunity is measured. The work done by Schepens and colleagues, here, demonstrate that non-neutralizing antibodies induced in in vivo against the small hydrophobic protein (SHe) of RSV poses an alternative approach in designing an RSV vaccine and is additionally important to others in the field interested in developing novel methods in mounting a protective response in vivo. The authors show that antibodies generated against SHe require Fc-receptor engagement - specifically through alveolar macrophage, to clear RSV-infected cells (as SHe antibody tend to recognize infected cells and not viruses).

However, with the current data, there is weak evidence alveolar macrophages play a major role in the clearance of RSV-infected cells. As shown in Figure 5, knock out of about 75% of alveolar macrophages (Figure 5B) only increased pfu/lung by about two-fold (Figure 5C; SHe-KLH PBS vs SHe-KLH Cl. Lip.). Although the values may be significant, it is hard to believe that this can correlate to the logs differences of lung titers found in the other experiments. Have the authors considered looking at different time points besides 5 days post infection - perhaps the authors should do a time course experiment to see how these alveolar macrophages could affect viral replication.

To provide more evidence that alveolar macrophages are important for protection by SHe-based antibodies, we have performed new experiments, including analysis at different time points after infection, with or without depletion of alveolar macrophages. In the original manuscript the role of alveolar macrophages was evaluated at 5 days post infections. We now tested the impact of alveolar macrophage depletion on viral replication at 4 and 6 days after HRSV challenge. To this end, 3 days before infection, mice were treated intranasally with clodronate-loaded liposomes or with PBS. In addition, the mice were treated with KLH or SHe-KLH immune serum one day before and one day

after HRSV challenge. At 4 and 6 days after infection with 1×10^6 PFU of HRSV A2, the lungs were collected for viral titration. On day 4 after infection, mice with an intact alveolar macrophage compartment that had received SHe-KLH immune serum had approximately 10-fold lower virus titers as compared with KLH serum recipients. In contrast, in mice that were treated with clodronate-loaded liposomes there was only a 2-fold reduction in pulmonary HRSV titer in SHe-KLH immune serum treated mice as compared to KLH immune serum treated mice. This 2-fold difference is not statistically significant. In addition, SHe-specific IgG levels were comparable in PBS and clodronate-loaded liposome treated mice that had been passively immunized with SHe-KLH immune serum. These data, which are shown in the **new Figure E7**, indicate that alveolar macrophages are pivotal for the reduction in HRSV replication by SHe-specific antibodies at early time points during infection. We also investigated viral replication at 6 days post-infection. In PBS treated mice that had received SHe-KLH immune serum, the lung viral titers were reduced approximately 110-fold as compared to the corresponding KLH immune serum recipients (Figure E7C). In mice that were treated with clodronate liposomes, the lung viral titer in SHe immune serum treated mice was about 15-fold lower than in KLH immune treated mice (Figure E7D). Although this 15-fold difference in viral titer was not statistically significant (1-way anova, Kruskal-Wallis) there is a clear trend towards lower virus titers in mice treated with SHe immune serum as compared to control serum treated mice. These data suggest that at later time points during infection, next to alveolar macrophages also other leukocytes might be involved in the reduction in HRSV replication by SHe-specific antibodies. These results represent a time course experiment and are included in the revised manuscript as figure E7. Because at later time points during infection also leukocytes other than resident alveolar macrophages might be involved in SHe antibody mediated protection, we have replaced in the abstract "alveolar macrophages are required" by "alveolar macrophages are involved". In addition, based on the viral titers at day 6 after infection in the context of alveolar macrophage depletion, we have added the following in the results section:

"These data indicate that at later time points during HRSV infections other, possibly infiltrating, leukocytes aid at reducing HRSV replication in the lungs."

Additionally, the authors should also look how complement may be involved in virus-infected cells as was done by Terajima and colleagues (JVI December 2011, vol. 85, no. 24).

We performed new experiments to analyze the possible involvement of complement as suggested by referee #2. Next to direct neutralization and FcγR mediated antibody-dependent cell-mediated cytotoxicity (ADCC) or antibody-dependent cellular phagocytosis (ADCP), antibodies can also directly hamper viral replication by complement-dependent lysis (CDL) of infected cells or viruses. Terajima *et al*. investigated whether influenza A hemagglutinin-specific human monoclonal antibodies (including non-neutralizing, stem-binding broadly neutralizing and globular head-binding subtype-specific neutralizing antibodies) can mediated CDL. To test this, influenza A virus infected A549 cells were incubated with human monoclonal antibodies combined with or without complement for 2 hours. We followed a similar protocol to investigate the potential role for CDL in SHe-specific antibody mediated reduction of HRSV replication. HRSV or mock infected A549 cells were incubated with either PBS or heat inactivated KLH, SHe-KLH or convalescent HRSV immune serum in the presence or absence of complement. One and a half hours and 18 hours later the supernatant was collected and tested for the presence of lactate dehydrogenase (LDH). The left hand graph of **Figure R1A** below, shows that HRSV infection is associated with enhanced LDH release as compared to mock infected cells. Incubation of cells with antisera for 1.5 hours, in the presence or absence of complement does not increase cell lysis. The right hand graph of Figure R1A illustrates that at 18 hours post infection, cell lysis due to HRSV infection is increased. Although complement enhanced lysis of infected cells, this was independent from the presence of SHe-specific immune serum or anti-HRSV immune serum. We repeated this assay with different concentrations of SHe and anti-HRSV immune sera, but we could not observe antibody-dependent CDL of HRSV infected cells.

Next to measuring direct lysis of infected cells, we also investigated whether complement could facilitate in vitro HRSV neutralization. To this end plaque reduction assays were performed with heat inactivated KLH, SHe-KLH and convalescent mouse HRSV immune serum in the absence or presence of exogenously added low-tox rabbit complement. Complement modestly enhanced the neutralizing activity of mouse HRSV convalescent serum (Figure R1B, lower right graph). As expected, SHe-KLH immune serum did not prevent HRSV plaque formation. Although addition of complement had a modest impact on HRSV plaque formation in the presence of SHe-KLH immune serum, this was also the case in the presence of negative control KLH immune serum. This is in line

with the neutralization assays described in the original manuscript that showed that non-heat-inactivated SHe immune serum does not neutralize HRSV infection in vitro (Figure 1D in the original manuscript). In summary, using two different in vitro approaches, we found no evidence for a role of the complement system in SHe-specific antibody mediated reduction of HRSV replication.

It remains possible that in vivo, complement contributes to the control of HRSV by SHe-specific IgG. Such a contribution can be investigated in mice by depleting the complement via the use of cobra venom factor treatment or by the use of complement deficient KO mice. However, our observation that Fcγ Receptors are essential for the protective effect of SHe-specific antibodies indicates that complement, at least in the absence of Fcγ Receptors, does not play a crucial role in protection by SHe-specific antibodies. As the complement system can affect FcγR functions in multiple ways (recruitment of macrophages, FcγR expression, competition,… Schmidt and Gessner, Immunol. Lett., 2005, p. 56-67) it would be interesting to investigate the role of the complement system in SHe antibody mediated reduction of viral replication in the future. However, we believe that such experiments require a substantial number on in vivo experiments and are beyond the scope of the present manuscript.

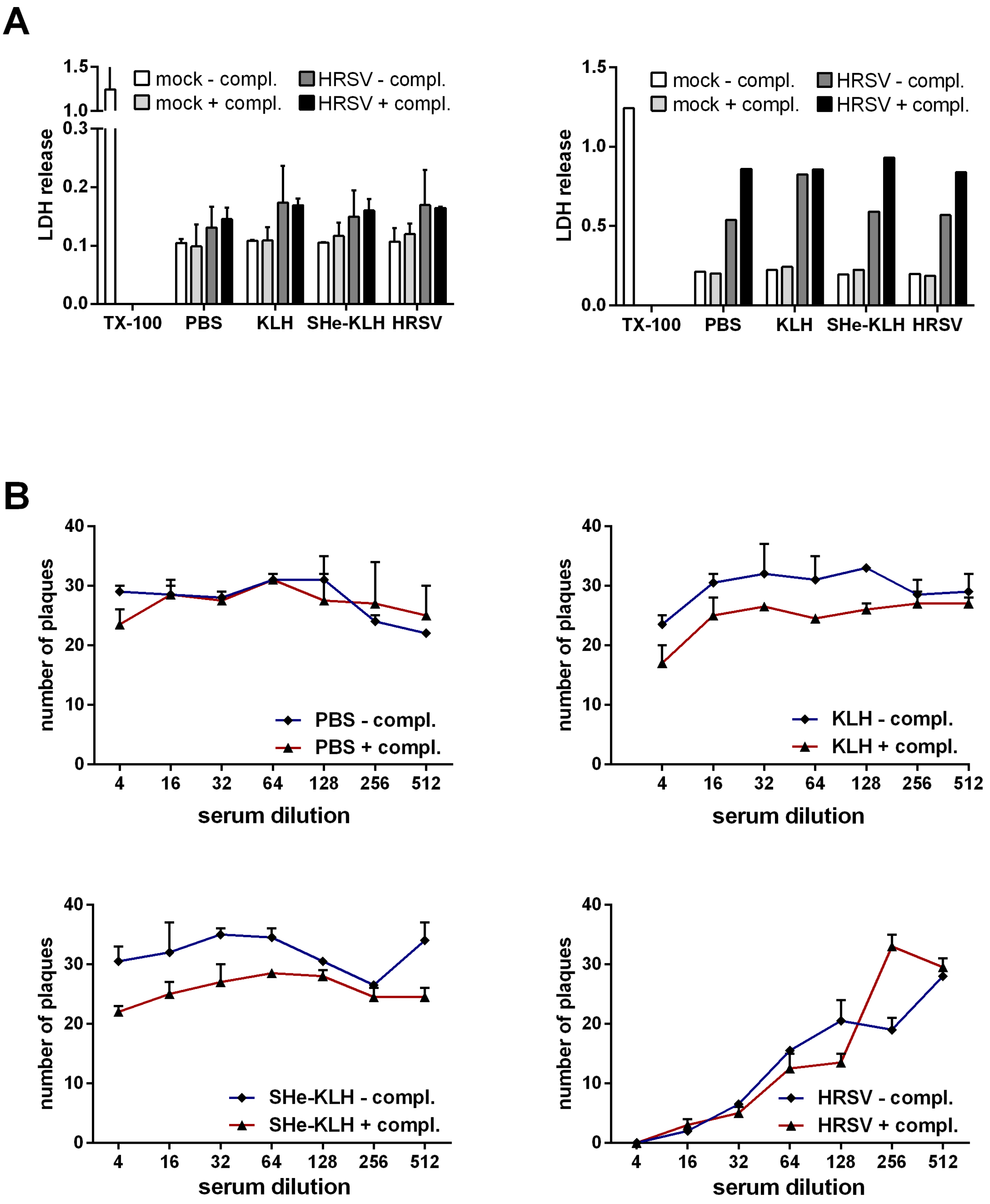

Figure R1. Analysis of a potential role for the complement system in SHe-specific antibody mediated reduction of HRSV replication. (A) Examination of a potential role for SHe-specific antibody mediated complement dependent lysis. A549 cells were mock infected or infected with 2 MOI of HRSV A2 (a concentration at which we observed by immunostaining that the majority of the cells are infected at 18 hours post infection). Eighteen hours after infection, the cells were washed and incubated with PBS or heat inactivated KLH immune serum (KLH), SHe-KLH immune serum (SHe-KLH) or serum derived from mice that were previously infected with HRSV (HRSV), with or without low-tox rabbit complement (Cedarlane, CL3051). The final dilution of the sera and the low-tox rabbit complement were respectively 1:200 and 1:20. As positive control for cell lysis (maximum lysis) cells were incubated with triton X-100 detergent (TX-100). Supernatant samples were taken at 1.5 and 18 hours and analyzed for the presence of lactate dehydrogenase (LDH) by the use of an colorimetric assay (Cytox non-radioactive cytotoxicity assay, Promega). The left and right graph respectively shows the mean measured O.D. value at 490 nm of the samples collected at 1.5 hours and 18 hours. (B) Examination of a potential role for complement in enabling SHe-specific

antibody mediated in vitro HRSV neutralization. Indicated dilutions of KLH, SHe-KLH or HRSV immune serum were mixed with 1:20 low-tox rabbit complement and 40 PFU HRSV A2 in a final volume of 50ul. After incubation for 30 minutes at 37°C, the mixtures were added to confluent Vero cells, grown in 96 well tissue culture plates. After 3 hours incubation at 37°C, 50 µl of growth medium containing 2% FCS and 0.6% avicel RC-851 (FMC Biopolymers) was added. Three days after infection, plaque formation was visualized by immunostaining with goat anti-HRSV serum and HRP-coupled anti goat IgG. The stained plaques were visualized by using TrueBlue peroxidase substrate (KPL, Gaithersburg, U.S.A.) and counted. The graphs show the mean (n=2 ± SEM) number of plaques of duplicate plaque reduction assays in which as indicated PBS or different dilutions of KLH, SHe-KLH or anti-HRSV (HRSV) immune sera in the presence (+ compl.) or absence (- cmpl.) of complement were tested.

Specific comments/corrections:

1) In the first paragraph of the introduction, the authors mentioned that there is no effective anti-viral or anti-inflammatory therapy for human respiratory syncytial virus. It would be beneficial for the readers if the authors mention a couple of countermeasure currently used in the introduction section (mention of these countermeasure is noted in the discussion section), besides the ones the authors pose - namely, palivizumab (a monoclonal antibody against the fusion protein) and ribavirin (guanosine analogue).

We agree with this suggestion and have now included the following section in the introduction of the revised manuscript:

"In high risk infants severe HRSV bronchiolitis can be prevented by prophylactic treatment with palivizumab, a HRSV neutralizing monoclonal antibody (Impact-RSV study group, 1998). Palivizumab, or its affinity-matured variant Motavizumab have however no therapeutic benefit (Ramilo et al., Pediatr Infect Dis J, 2014). A Cochrane study concluded that therapeutic treatment with aerosolized ribavirin, a guanosine analogue with antiviral activity against both RNA and DNA viruses, might reduce mortality and days of hospitalization in infants with severe HRSV infection (Ventre et al., The Cochrane database of systematic reviews, 201407). Due to its potential teratogenicity, ribavirin is not generally used to treat HRSV-associated illness."

2) It may also be helpful for the readers if the authors also give a brief description (or at least what is known about it) on the role of the small hydrophobic protein in the viral replication cycle.

We agree that this would be informative for the reader and included in the revised manuscript the following section in the introduction:

"Although the exact function of SH remains poorly understood, it folds into pentameric cation-selective ion channels that can activate the NLRP3 inflammasome (Carter et al, 2010; Gan et al, 2012; Triantafilou et al, 2013). The importance of these functions remains unknown as recombinant HRSV that lacks SH expression is not attenuated in vitro and only slightly attenuated in mice and non-human primates (Bukreyev et al, 1997; Whitehead et al, 1999)."

3) In the last sentence of the first paragraph on page 7, I think the authors were referring to figure 2F to H instead of 3F to H.

This mistake has been corrected in the revised manuscript.

4) In the first paragraph of page 8, the authors should also mention that they also treated the mice with ¬¬¬'not immunized (NI)' sera as one of their controls.

We agree and included the following sentence in the Result section of the revised manuscript:

"Mice that were not treated and not infected (NI) were used as negative control for HRSV induced bodyweight loss."

5) Figure 5B, y-axis: a better abbreviation of 'number' would be 'No.' rather than 'Nbr'.

This is corrected in the revised manuscript.

6) Page 10; Figure E4: Showing that Fc-gamma-R1-specific antibodies bind to WT macrophages and not the ones found in knock-out mice by immunofluorescence is visually pleasing, however,

perhaps using FACs analysis to measure binding to WT vs KO macrophages would be a better way (to show quantitatively) that they are truly not binding to the KO alveolar macrophages.

We agree with the referee that the immunofluorescence micrograph is not very quantitative to document expression of FcγR I (CD64) on alveolar macrophages. We have now performed flowcytometric analysis to confirm Fcγ Receptor I expression in murine alveolar macrophages. Bronchoalveolar Lavage Fluild (BALF) cells of wild type mice and FcγRI$^{-/-}$ BALB/c mice were isolated and stained with an Alexa647 conjugated anti-mouse FcγRI specific antibody or an APC conjugated isotype control antibody. Under steady state conditions resident alveolar macrophages represent the vast majority of BALF cells. Resident alveolar macrophages were also identified as auto-fluorescent (green channel), CD11c positive cells. The **new Figure E5** in the revised manuscript illustrates that, in contrast to FcγRI$^{-/-}$ alveolar macrophages, wild type alveolar macrophages are readily recognized by anti-FcγRI antibodies. These data confirm that alveolar macrophages do indeed express FcγRI. In the revised manuscript the microscopic images have been omitted and replaced with a new Figure E5, showing the FACS histograms.

Referee 3.

Referee #3 (Comments on Novelty/Model System):

Reasons for these choices can be found in my comments to authors

Referee #3 (Remarks):

The paper by Schepens et al. explores the performance of a respiratory syncytial virus (RSV) vaccine based on the extracellular domain of the small hydrophobic (SH) protein of human RSV. The authors synthesized a 23 amino acid peptide based on the SH ectodomain sequence of a group A RSV strain and linked it to keyhole limpet hemocyanin (KLH). The SHe-KLH conjugate was used to immunize mice and cotton rats searching for: i) peptide specific antibody responses and ii) protection against a RSV challenge. The results obtained demonstrate the induction of non-neutralizing antibodies specific for the SH ectodomain and some level of protection against a RSV challenge, suggesting a potential use of SH based vaccines in the future. Passive transfer of immune serum and depletion of certain cell types provided evidence that the SHe-KLH vaccine confers protection by non-neutralizing antibodies that act by some kind of antibody-dependent cellular mechanism likely involving alveolar macrophages. The results are clearly presented and generally support the conclusions reached by the authors.

I think that this unconventional SH based vaccine merits the investigation carried out by Schepens et al., but I feel that relevant information is missing in the manuscript to appreciate the vaccine potential. Thus, the authors should provide as much data and/or information as they have about the following main points:

1.- Duration of protection afforded by the SHe-KLH vaccine. There have been reports of RSV vaccines based on the N or M2-1 proteins that induced short lived protection, while vaccines based on G or F glycoproteins afforded long lasting protection. Is the new SH based vaccine of the first or second type?

Like referee 1, referee 3 wonders whether the protection elicited by SHe-based vaccination is short or long living. We suspect that Referee 3 refers to a seminal publication by Connors and colleagues where they demonstrated by using Vaccinia virus as vector that vaccination with the HRSV proteins F and G induces long living protection. In contrast vaccination with the Vaccinia vector encoding HRSV proteins N or M2-1, afforded only short living protection (Connors et al., J. Virol., 1991, p.1634-1637). In this report by Connors et al., short living duration was tested by challenge at 9 days after immunization whereas long living duration was tested by viral challenge at 28 days after immunization.

We performed two new experiments to evaluate the duration of protection by SHe-based immunization. The results of these experiments are included in the revised version of our manuscript in two new figures: **Figure E1 and Figure 2**.

For the first experiment, we used BALB/c mice from an ongoing immunization experiment that had been set up prior to the receipt of the referee reports and was initially intended to produce immune sera. This experiment comprised two groups of six BALB/c mice that had been immunized by intraperitoneal injection of 20 µg KLH or SHe-KLH adjuvanted with incomplete Freund's adjuvant (IFA). Immunizations were performed 3 times with 3 weeks interval. These animals were challenged 11 weeks (instead of 3 weeks) after the last immunization with 1 x 10^6 PFU HRSV. Six days after challenge, the lungs were collected to determine the pulmonary HRSV titer. Figure E1A and E1B illustrate that at 11 weeks after the last immunization all SHe-KLH immunized mice contained high levels of SHe-specific serum IgG that were only slightly lower than the levels 4 weeks after the last immunization. Importantly, challenged SHe-KLH immunized mice had a significantly, 10-fold lower pulmonary HRSV titer than KLH immunized control mice (Figure E1C). The body weight relative to the day of challenge was comparable for the two groups (Figure E1D).

In a second experiment we used five groups of BALB/c mice. We included an additional negative control group of mice immunized with PBS without adjuvant and we evaluated the performance of an alternative adjuvant: Sigma Adjuvant System (SAS), an oil-in-water adjuvant containing Monophosphoryl Lipid A. Two groups of mice were immunized intraperitoneally with SHe-KLH in combination with IFA or with SAS. As negative control mice were immunized with non-conjugated KLH in combination with the respective adjuvants. Immunizations with PBS, KLH/IFA and SHe-KLH/IFA were performed 3 times with 2 weeks interval. Immunizations with KLH/SAS and SHe-KLH/SAS adjuvant were performed 2 times with 2 weeks interval. As a third "immunization" the latter two groups of mice received PBS without adjuvant. Six weeks after the last immunization (this is 8 weeks after the last immunization with SAS), the mice were challenged with 1 x 10^6 PFU of HRSV A2. As shown in the new Figures 2A and 2B, three weeks after the last immunization most mice had high levels of SHe-specific serum IgG1 and IgG2a titers. Six days after HRSV challenge, the pulmonary HRSV titers of mice that were immunized with SHe-KLH in combination with IFA were significantly lower (about 30-fold) than the viral titer in mice that were immunized with non-conjugated KLH (Figure 2C). Similarly, also mice that were immunized with SHe-KLH combined with SAS had significantly lower pulmonary HRSV titers than the KLH/SAS mice. Although at 6 days post infection no significant bodyweight loss was observed, mice that were vaccinated with SHe-KLH either in combination with IFA or SAS tended to have a somewhat higher relative bodyweight as compared to the KLH control mice (Figure 2D).

In our opinion, the data from these two new experiments indicate that protection by SHe-KLH vaccination, which does not induce neutralizing antibodies, is of the second type: relatively long living in mice.

2.- Given the sequence differences between the SH ectodomains of RSV group A and group B viruses, what is the degree of cross-protection (if any) afforded by the SHe-KLH vaccine? Is there any cross-reactivity of the antibodies induced by SH from group A with the SH peptide from group B and vice versa?

Although the SHe amino acid sequence is well conserved within the HRSV A and within HRSV B subtypes, there is only minimal overlap (underlined sequence) between SHe of HRSV A (SHeA: NKLCEYNVFHNKTFELPRARVNT) and HRSV B (SHeB: NKLSEHKTFCNKTLEQGQMYQINT). Given the sequence difference between HRSV A and HRSV B subgroup viruses, it is fair and relevant to question the degree of cross-reactivity and hence potential cross-protection between HRSV A and B SHe. To evaluate the potential cross-reactivity between SHe of HRSV A and HRSV B viruses, KLH, SHe-KLH and HBc-SHeB immune sera were tested on SHeA and SHeB peptide ELISA. As shown in **the revised Figure E2I and –J**, SHeA peptide is bound by SHeA-KLH immune serum and not by HBc-SHeB immune serum. Accordingly, SHeB peptide can only be bound by HBc-SHeB immune serum and not by SHeA-KLH immune serum. In addition to peptide ELISA, the potential cross-reactivity of SHeA and SHeB was also tested by immunostaining of cells infected with HRSV A2 or a clinical HRSV B isolate JX576731. **Figure R2** bellow, illustrates that SHeA-KLH immune serum recognizes HRSV A2 plaques and not HRSV B plaques, whereas HBc-SHeB immune serum recognizes HRSV B and not HRSV A2 plaques. Together, these data suggest that there is no or minimal cross-reactivity of antibodies induced by SHe derived from group A against group B and vice versa. Hence it is unlikely that SHe vaccination would have any cross-protective activity. As a consequence a SHe-based HRSV vaccine candidate should contain both the SHeA and the SHeB antigen.

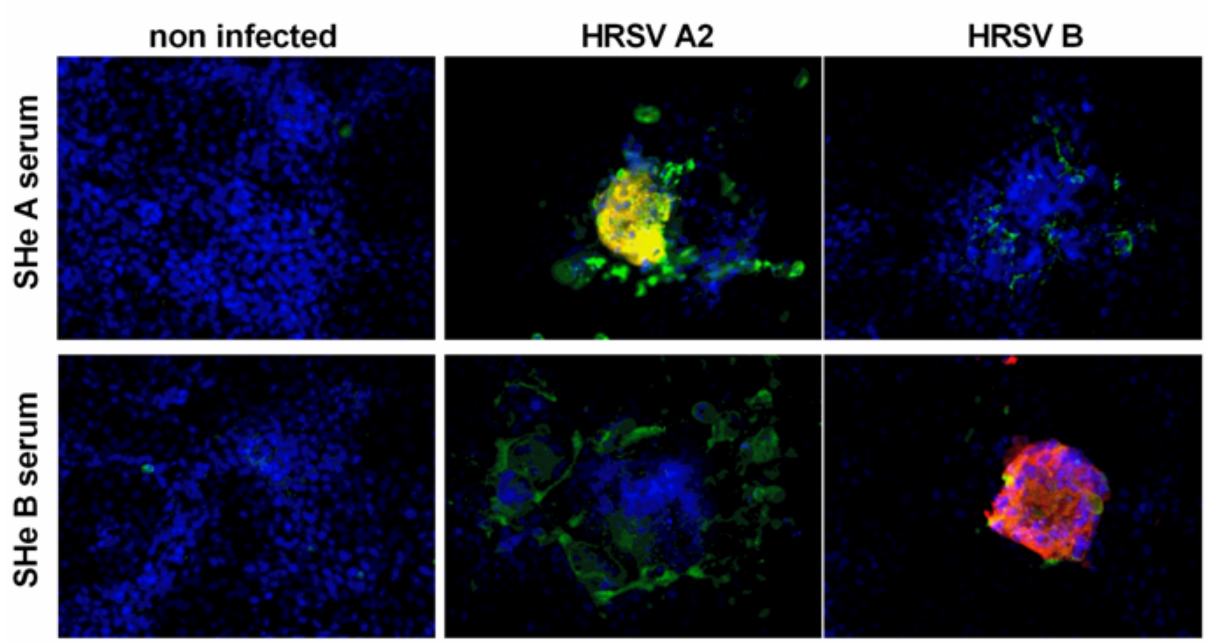

Figure R2. SHe immunization does not induce cross-reactive antibodies that recognize both HRSV A and HRSV B SH ectodomains. Vero cells were infected with 0.001 MOI of either HRSV A2 or the clinical HRSV B isolate JX576731 or mock infected. Two days after infection the cells were washed with PBS, fixed with 1% PFA and blocked with 1%BSA. Subsequently the viral plaques were stained with goat anti-HRSV immune serum (detected with Alexa488 conjugated anti-goat IgG, green signal) in combination with a 1/200 dilution of either SHeA-KLH and HBc-SHeB mouse immune serum (detected with Alexa555 conjugated anti-mouse IgG, red signal). The used SHe mouse sera were pre-cleared by overnight incubation with detached Vero cells at 4°C. The nuclei were stained with DAPI (blue). The images represent the overlay of the 3 different stains.

3.- What are the basis for the dose used in the immunizations? It would be helpful if the authors could provide data or information about dose effects that justify the chosen amount of SHe-KLH conjugate. The same apply to the passive administration of immune serum. Why 35 µl of serum and why intranasal administration? In other words, are the conditions used for this work optimal or we may expect even better performance of the SHe-KLH vaccine?

The use of a dose of 20 µg SHe-KLH antigen is based on our prior experience with the use of Influenza A M2e, which is also a 24 amino acid long peptide. Immunizations with 5 or 10 µg of M2e genetically conjugated to different protein carriers consistently gave good immune responses. Because our SHe-KLH construct is a chemical, and thus less well controlled, conjugate we decided to use 20 µg as antigen dose for immunizations. We performed new experiments to investigate the dose response of SHe-KLH antigen amounts. Groups of 2 BALB/c mice were immunized with different amounts of SHe-KLH ranging from 80 µg to 0.3 µg in combination with IFA. As negative control 80 µg of unconjugated KLH in combination with IVA was used. As shown in **Figure R3** below, after 3 intraperitoneal immunizations with 3 weeks interval mice that were immunized with 80, 20, 5 or 1.25 µg had comparable levels of SHe-specific serum IgG, IgG1 and IgG2a whereas immunization with 0.3 µg elicited lower levels of SHe-specific serum IgG and IgG2a. These data demonstrate that the dose of antigen can be reduced to 1 µg per immunization without significant loss of seroconversion.

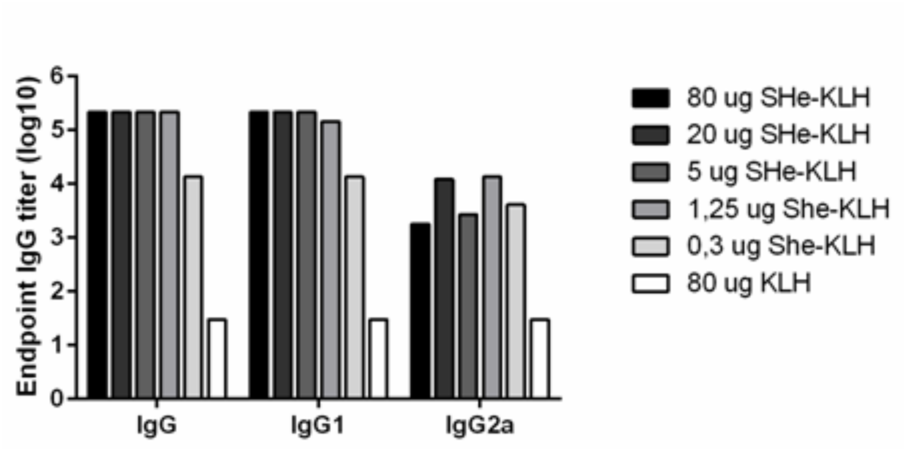

Figure R3. Dose response in active immunization. The induction of SHe-specific IgG is dependent on the used antigen dose. Groups of 2 BALB/c mice were immunized with either 80, 20, 5, 1.2 or 0.3 µg of SHe-KLH or 80 µg of KLH, adjuvanted with IFA. Three immunizations were performed with 3 weeks interval. Twenty days after each immunization sera were prepared and tested in a SHe peptide ELISA. The graph shows the mean endpoint titers of SHe-specific serum IgG, IgG1 and IgG2a of each indicated group of immunized mice.

To investigate the impact of the dose used for intranasal passive immunization with SHe-KLH immune serum, 4 groups of 6 mice were treated one day before and one day after infection with 50, 25, 5 or 0 µl of SHe-KLH immune serum. All administrated sera were adjusted to a final volume of 50 µl with KLH immune serum. Five days after challenge with 1 x 10^6 PFU of HRSV A2, the lungs were collected for HRSV titration. The results of this challenge experiment are presented in **new Figure E4A** and show that inhibition or HRSV replication by SHe immune serum at 5 days post infection is dose dependent. Whereas a dose of 5 µl SHe immune serum is able to reduce the lung HRSV titer by 3-fold, 25 and 50 µl SHe immune serum respectively reduce the lung HRSV titer by 11 and 17-fold. None of the mice displayed bodyweight loss during the experiment (**Figure E4B**).

As mentioned above in the response to referee 1, also intraperitoneal administration of SHe immune serum reduces HRSV replication (**new Figures E4C, E4D and E8**). However 400 µl of immune serum was required to obtain an 8-fold reduction in lung HRSV titers at 5 days post infection. In comparison, 35 µl of SHe immune serum administered intranasally was routinely used and is suited to obtain consistent and significant reduction in HRSV titers. Therefore, intranasal administration of SHe immune serum is an efficient way to reduce HRSV replication upon subsequent challenge and is also a way to reduce the number of mice required to obtain immune serum for passive immunizations.

Other points:

1.- Page 3, line 6: Is Haynes et al., the correct reference here? Please, check.

We apologize for this mistake, which is now corrected in the revised manuscript: we wanted to refer to Hall et al., 1991.

2.- Given the sequence divergence between the SH ectodomains of group A and group B viruses, I miss clear data/information about the degree of cross-reactivity of the respective antisera against the heterologous peptide and about the degree of cross-protection afforded in challenge studies. I may have missed this information but if so, it was not clear in the paper.

For this remark, we respectfully refer to our response to remark 2 of the referee and to the new figures E2I, E2J, the sequence alignments in Figures E10 and E11 and the following statement in the results section:

"The amino acid sequence of SHe is highly conserved among the group A HRSV A viruses but differs substantially from SHe in group B HRSV viruses where it is also sequence conserved (Fig E10 and E11) (Collins et al, 1990)."

And newly added:

"To test the potential cross-reactivity between SHe of HRSV A and HRSV B viruses, KLH, SHe-KLH and HBc-SHeB immune sera were tested in a SHeA and SHeB peptide ELISA. Figures E2I-J show that SHeA peptide is bound by SHeA-KLH immune serum and not by HBc-SHeB immune serum. Accordingly, SHeB peptide can only be bound by HBc-SHeB immune serum and not by SHeA-KLH immune serum. Hence to cover both HRSV A and HRSV B viruses a SHe-based vaccine should contain both the SHeA and SHeB antigen."

3.- The use of hepatitis B virus like particles is described only in the expanded view part of the manuscript. The generation of these particles and conjugation of the SH peptide should be described in Materials and Methods. Why not using the KLH approach in this case?

Due to the insolubility of the SHeB peptide in the conjugation buffer used for chemical linkage to KLH, we were unable to generate a reliable and soluble SHeB-KLH conjugate. Instead, we were successful in generating such a conjugate using mHBcore as a carrier. We regret that the generation of this conjugate was not described in the Materials and Methods section. The description of how HBc-SHeB was produced in now mentioned in the material and methods section.

4.- The depletion of macrophages was done only in the mice that passively received immune serum. Why not depleting macrophages in mice immunized with SHe-KLH before challenge? This would be more informative about the role of macrophages in the full response afforded by the conjugate and not only by passively administered antibodies.

We completely agree with this remark. To test the role of alveolar macrophages in an active vaccination experiment, we performed a new experiment in which mice were vaccinated with KLH or SHe-KLH in combination with IFA. Nineteen days after the last immunization half of the mice were treated with PBS or clodronate loaded liposomes. We found that prior to treatment with PBS or clodronate loaded liposomes, SHe-KLH immunization induced SHe-specific IgG (**new Figure 6F**). Three days later all mice were challenged with 1 x 10^6 PFU of HRSV A2. Five days after challenge the lungs were collected for HRSV titration. As expected in the absence of treatment with clodronate loaded liposomes, SHe-KLH vaccinated mice had significantly lower (6.5-fold) lung HRSV titers than KLH vaccinated mice (**new Figure 6G**). In contrast in mice in which alveolar macrophages were depleted by clodronate loaded liposomes the lung HRSV titer of SHe-KLH and KLH vaccinated mice did not significantly differ (1.6-fold). These data show that also in SHe-KLH immunized mice, alveolar macrophages are important for protection. These data are included in the revised manuscript (new Figure 6F-G).

5.- As acknowledged by the authors, the impact of SHe protein vaccination on RSV replication is lower than the sterilizing immunity afforded by F-protein based vaccines. This is however not easily seen in the Figures, because the way data is represented. For instance, in Figure 2C the difference in PFU/gram lung between HRSV and SHe-KLH groups seems minimal, since all HRSV values are on top of the abscissas' axis. A log representation of these data would be more illustrative. Please, change Figures to offer a clear picture of SHe-KLH performance in comparison with HRSV.

Referee 3 suggests to use a log scale for showing HRSV pfu levels. In all vaccination experiments described in figures 1, 2, 3, 4, 5, 6F-G, E1, E6 and E7 of the revised manuscript the viral titers are now shown in log 10 scale. Still, in some experiments, in which the possible contribution of different cell types (NK cells, alveolar macrophages and T cells) or dose effects were investigated as described in figures 6A-E, E4, E5 and E9, we prefer to depict the lung viral titers in a linear scale to allow better visualization of small differences.

I found the Discussion extremely long and cumbersome. Please, shorten it by avoiding repeating what it was already mentioned and concluded under Results.

We agree with this comment and have now shortened the discussion.

The heading of the second Figure E6 should be "Figure E7".

We apologize for this mistake and have corrected this in the revised manuscript.

8.- Figure 7D: The sera from RSV infected mice reacted with SHe-KLH but in Figures 2A and 2B the sera of RSV infected mice failed to react with the same conjugate. Please, clarify.

Refree 3 notes that in figure 7D of the original manuscript sera from HRSV infected mice react with SHe-KLH in an ELISA, but not in figures 2A and 2B of the original manuscript. This is however a misinterpretation due to confusing headings in figure 7D. Figure 7D shows the binding of different

sera, including SHe-KLH serum, to plates coated with lysates of HRSV infected cells; not the binding of HRSV immune serum to SHe-KLH coated plates. Panel D shows an ELISA with coated SHe peptide in which HRSV serum fails to bind. We have adapted the headings in the panels of this figure (now numbered as Figure 8 in the revised manuscript) to avoid confusion by the reader.

In summary, addition of more information and data will help to illustrate the full potential of the SH based RSV vaccine as well as polishing some of the aspects raised in the comments mentioned above will improve the manuscript clarity.

We believe that by responding to all the remarks of the referees and by performing new experiments, we have provided additional information and new data to illustrate the potential of the SH-based vaccine approach.

2nd Editorial Decision 26 August 2014

Thank you for the submission of your revised manuscript to EMBO Molecular Medicine. We have now received the enclosed reports from the referees that were asked to re-assess it.

As you will see, the reports are conflicted so we asked for an additional advice from an active scientist expert in the field. While referee 1 is now fully satisfied and supports publication, referees 2 (mainly) and 3 remain cautious about the findings and their significance for the scientific community. Our expert adviser summarised well the situation and added that the antigen used in this study will unlikely be used as a stand alone vaccine as it is (as you mention too) inferior to the F-protein based vaccines. However, the novel mechanism and target provide interesting insights that could be used in combination with more classical approaches. Therefore, for the paper to be formerly accepted, we would need you to rephrase the abstract, introduction, discussion and The Paper Explained accordingly to better reflect this fact. There is no need to completely reformat the article, only better justifications on how this novel vaccine could be used in humans.

Please submit your revised manuscript within two weeks.

I look forward to reading a new revised version of your manuscript.

***** Reviewer's comments *****

Referee #1 (Comments on Novelty/Model System):

This is a revised manuscript.

Referee #1 (Remarks):

The Authors have addressed my criticism in great details. I find the paper improved and acceptable for publication.

Referee #2 (Remarks):

The authors addressed referees' 1 and 3 question about the longevity of protection afforded by the vaccine with a challenge experiment 11 weeks post vaccination. Three months is on the short side of longevity studies and this experiment may be insufficient to argue: "The reduction of HRSV in SHe-KLH immunized mice is not short living".

HRSV lung titers were used as correlates of protection. Body weight differences are repeatedly mentioned throughout the manuscript, and the authors claim to observe tendencies towards less

weight loss for vaccinated mice. This begs the question why they did not perform prior virus titration experiments to ensure weight loss. Switching to a more mouse pathogenic strain or increasing the number of animals could have helped.

The investigation of additional time points post challenge, as suggested by referee 2 confirmed a crucial role of alveolar macrophages in the clearance of HRSV infection in mice, but also added to this referee's doubts about them being solely responsible for the effect.

The investigation of the complement system in vivo, as suggested by referee 2, would indeed be interesting but could be part of a future manuscript focusing on the involvement of various compartments of the immune system.

The authors addressed the question by referee 3 regarding group A and B cross-protection. They did not see antibody cross-reactivity in a peptide ELISA, which they ascribe to the minimal sequence overlap between the group A and B SH ectodomains. The SHe domains are however well conserved within their respective groups. It would be important to show that vaccination with one specific group A SHe domain protects against a broad range of various group A strains.
In summary, investigations on the breadth of protection against various strains and true longevity studies are necessary to justify the conclusions made in the present paper.

Referee #3 (Remarks):

Schepens et al. have made great effort to respond in this resubmission to the criticisms raised by the reviewers to the original manuscript. I do not find any further caveat that requires experimental work. Yet, I still find that the protection afforded by the SH vaccine against experimental infection of mice or cotton rats with human RSV is very limited. I wonder what future this vaccine may have in humans.

2nd Revision - authors' response 08 September 2014

Referee 1.

Referee #1 (Comments on Novelty/Model System):

This is a revised manuscript.

Referee #1 (Remarks):

The Authors have addressed my criticism in great details. I find the paper improved and acceptable for publication.

Authors' response:

We are very happy with this comment

Referee 2.

Referee #2 (Remarks):

The authors addressed referees' 1 and 3 question about the longevity of protection afforded by the vaccine with a challenge experiment 11 weeks post vaccination. Three months is on the short side of longevity studies and this experiment may be insufficient to argue: "The reduction of HRSV in SHe-KLH immunized mice is not short living".

Authors' response:

We did our best to postpone the challenge as long as possible after vaccination, taking into account the timeline of the revision process (3 months + 1 month extra granted). Published data on protection against HRSV induced by vaccination are very frequently performed on 2-6 weeks after the last immunization. We used "not short living" based on the paper by Connors et al (JV, 1991, p. 1634-) in which protection that had waned by day 28 after immunization with M2 and N constructs was considered as "relatively short-lived".

HRSV lung titers were used as correlates of protection. Body weight differences are repeatedly mentioned throughout the manuscript, and the authors claim to observe tendencies towards less weight loss for vaccinated mice. This begs the question why they did not perform prior virus titration experiments to ensure weight loss. Switching to a more mouse pathogenic strain or increasing the number of animals could have helped.

Authors' response:

We agree with this comment. We used clinical isolates that had been amplified on HEp2 cells to obtain a more pathogenic challenge model in mouse. However, none performed better than RSV A2 in our hands.

The investigation of additional time points post challenge, as suggested by referee 2 confirmed a crucial role of alveolar macrophages in the clearance of HRSV infection in mice, but also added to this referee's doubts about them being solely responsible for the effect.

Authors' response:

The evidence for the involvement of alveolar macrophages is based on conditional cell depletion experiments, using clodronate-loaded liposomes. Based on multiple experiments, involving passive and now also active immunization experiments with SHe antibodies and SHe-KLH conjugate, respectively, we consistently found that such depletion significantly reduced HRSV replication in challenged mice. Making conclusions based on a single experimental approach is not ideal. In the future, we plan to perform reconstitution experiments in which alveolar macrophages are re-introduced in mice that had been treated with clordonate liposomes.

Finally, we expressed our prudence on the involvement of alveolar macrophages in SHe-based immune protection in the discussion "Alternatively, this may indicate that other Fcγ R expressing cells such as e.g. infiltrating monocytes, NK cells and neutrophils could also be involved, especially at later time points during infection (Fig E7)."

The investigation of the complement system in vivo, as suggested by referee 2, would indeed be interesting but could be part of a future manuscript focusing on the involvement of various compartments of the immune system.

Authors' response:

We investigated the involvement of the complement system for SHe-based immune protection following the suggestion of referee 2. We interpreted "Additionally, the authors should also look how complement may be involved in virus-infected cells as was done by Terajima and colleagues (JVI December 2011, vol. 85, no. 1634-1637)." as a suggestion to study the involvement of complement in vitro, as was done in the Terajima paper.

Experiments in complement-deficient mice with SHe-based vaccination strategies would help to further clarify the contribution of this component of the immune system in protection.

The authors addressed the question by referee 3 regarding group A and B cross-protection. They did not see antibody cross-reactivity in a peptide ELISA, which they ascribe to the minimal sequence overlap between the group A and B SH ectodomains. The SHe domains are however well conserved within their respective groups. It would be important to show that vaccination with one specific group A SHe domain protects against a broad range of various group A strains.

Authors' response:

We agree with this remark. We have so far exclusively used RSV A2 for challenge of mice, because this strain consistently replicated in mice and at high challenge dose also induced some weight loss in the mice. In the cotton rat experiment, strain RSV Tracy was used. Challenge experiments with other RSV A strains that replicate and are pathogenic in mice are planned in the future.

In summary, investigations on the breadth of protection against various strains and true longevity studies are necessary to justify the conclusions made in the present paper.

Authors' response:

Assessment of extended longevity of protection is ongoing. We plan to challenge mice at 4 and 6 months after immunization with SHe-KLH. To assess protection against a panel of RSV A viruses, we plan to obtain pathogenic RSV A strains from other laboratories.

Referee 3.

Schepens et al. have made great effort to respond in this resubmission to the criticisms raised by the reviewers to the original manuscript. I do not find any further caveat that requires experimental work. Yet, I still find that the protection afforded by the SH vaccine against experimental infection of mice or cotton rats with human RSV is very limited. I wonder what future this vaccine may have in humans.

Authors' response:

We are pleased with the appreciation for our efforts to respond to the concerns that had been raised by the reviewers.

We have now highlighted in our abstract, introduction, discussion and "the paper explained" that clinical development of SHe as a stand lone HRSV vaccine candidate is an unlikely path. Please see text highlighted in green in the manuscript text. Both its mechanism of action and the choice of this antigen, warrant investigation of the use of SHe antigen as an adjunct to vaccines that aim at inducing neutralizing antibodies or protective T cells.
