# Supplementary material for: Protection and mechanism of action of a novel human respiratory syncytial virus vaccine candidate based on the extracellular domain of small hydrophobic protein
Source: EMBO Mol Med. 2014 Oct 8;6(11):1436–54. doi: 10.15252/emmm.201404005 (PMC4237470; doi:10.15252/emmm.201404005)
Supplement: Supplementary file 2 [file emmm0006-1436-sd2.pdf]

Table of content:

Figure S1. Protection afforded by SHe-based vaccination is not short living. BALB/c mice were immunized with KLH or SHe-KLH combined with Incomplete Freund's adjuvant.

Figure S2. Immunization of mice with virus like particles conjugated with a peptide corresponding to the SH ectodomain of HRSV B subgroup viruses (SHeB) induces SHeB-specific antibodies and reduces HRSV B induced body weight loss and pulmonary cell infiltration.

Figure S3. SHe-KLH immunization reduces pulmonary HRSV replication in cotton rats without exacerbating the disease.

Figure S4. Passive immunization by intranasal and parenteral administration of SHe-KLH serum reduces HRSV replication.

Figure S5. Depletion of NK cells does not abrogate the impact of SHe immune serum on HRSV replication.

Figure S6. Alveolar macrophages express FcγRI at their surface.

Figure S7. A role for alveolar macrophages SHe immune serum mediated reduction of viral replication at 4 and 6 days post infection.

Figure S8. The reduction of HRSV replication by intraperitoneal treatment with SHe immune serum depends on alveolar macrophages.

Figure S9. Depletion of CD4 and/or CD8 cells does not abrogate the reduction of HRSV replication in SHe immunized mice.

Figure S10. Sequence conservation of the SH ectodomain of HRSV A subgroup viruses. Alignment of the SHe amino acid sequences.

Figure S11. Sequence conservation of the SH ectodomain of HRSV B subgroup viruses.

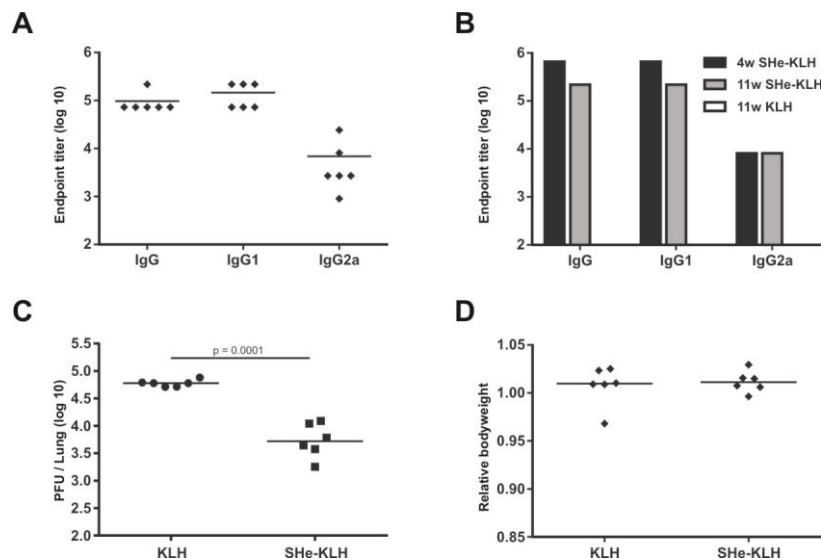

Figure S1. Protection afforded by SHe-based vaccination is not short living. BALB/c mice were immunized with KLH or SHe-KLH combined with Incomplete Freund's adjuvant. Serum was collected 4 weeks and 11 weeks after the third immunization. Eleven weeks after the last immunization the mice were challenged with 1×10^6 PFU HRSV A2. Six days after challenge the lungs were collected to determine the pulmonary HRSV titer by plaque assay. (A) Individual SHe-specific IgG, IgG1 and IgG2a titers in sera collected 11 weeks after the last immunization as determined by SHe peptide ELISA. The horizontal bar indicates the mean IgG titer. (B) Endpoint SHe-specific IgG, IgG1 and IgG2a titers of pooled sera of KLH and SHe-KLH vaccinated mice collected 4 or 11 weeks after the last immunization, as tested in a SHe peptide ELISA. (C) Vaccination with SHe-KLH reduces HRSV A2 replication in the lungs of challenged BALB/c mice. The graph shows the number of PFU per lung for each mouse, sampled 6 days after challenge with 1×10^6 PFU of HRSV A2 (2-sided unpaired t-test). The horizontal bar indicates the mean lung virus titer. (D) Vaccination with SHe-KLH is not associated with enhanced bodyweight loss upon HRSV infection. The graph shows the relative bodyweight at day 6 post infection calculated as the ratio between bodyweight at day 6 and bodyweight at day 0. Horizontal bars indicate the mean relative body weight.

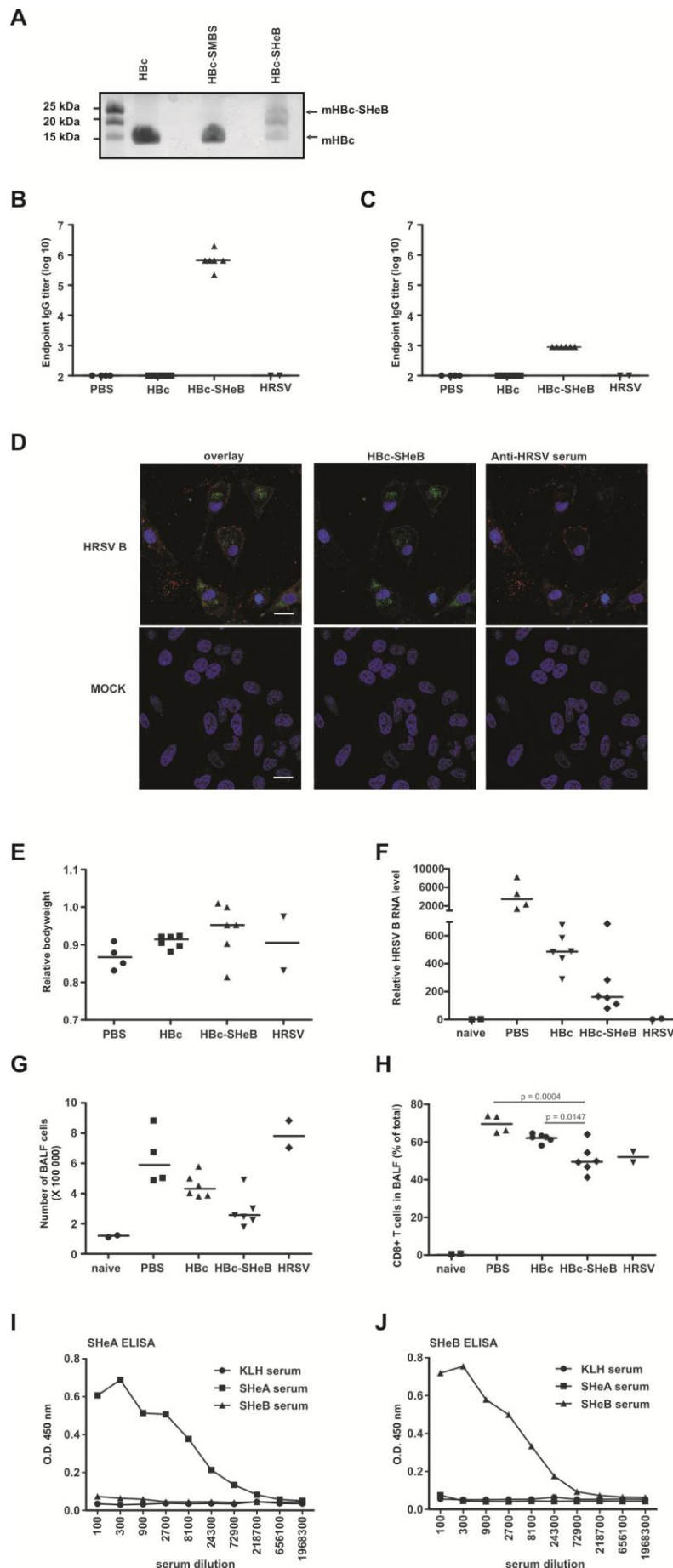

Figure S2. Immunization of mice with virus like particles conjugated with a peptide corresponding to the SH ectodomain of HRSV B subgroup viruses (SHeB) induces SHeB-specific antibodies and reduces HRSV B induced body weight loss and pulmonary cell infiltration. BALB/c mice were immunized 3 times intraperitoneally with 20 μ g HbC or 20 μ g HbC-SHeB (both in combination with incomplete Freund's adjuvant) or with PBS. Immunizations were performed at three-week intervals. Mice infected with 1×10^6 PFU HRSV A2 were used as controls. Sera were collected three weeks after the last immunization or nine weeks after HRSV A2 infection. Three weeks after the last immunization, the mice were challenged with 1.5×10^6 PFU of a clinical HRSV B isolate. Seven days post infection, bronchoalveolar lavage fluid (BALF) was collected to assess pulmonary leukocyte infiltration and HRSV B RNA levels. BALF of naive mice was used as negative control. (A) Conjugation of SHeB peptide to HbC virus like particles. The image shows a Coomassie blue stained SDS-PAGE gel loaded with unconjugated (HbC), Sulfo-MBS conjugated (HbC-SMBS) and Sulfo-MBS-SHeB conjugated HbC protein (HbC-SHeB). (B) Immunization of BALB/c mice with HbC-SHeB

induces SHeB-specific serum IgG. The Graph shows the endpoint titer of SHeB-specific IgG of each mouse at 3 weeks after the last immunization. Horizontal bars represent the mean IgG titer. (C) Immunization of BALB/c mice with HBc-SHeB does not induce high levels of SHeB-specific serum IgG. The Graph shows the endpoint titer of SHeA-specific IgG of each mouse at 3 weeks after the last immunization. Horizontal bars represent the mean IgG titer. (D) Serum from HBc-SHeB immunized mice recognizes SH at the surface of HRSV B infected cells. Vero cells were either infected with 5 MOI of a clinical HRSV B isolate or mock infected. Two days later they were fixed overnight with 2% paraformaldehyde, permeabilized and stained with sera from HBc or HBc-SHeB immunized mice (detected with Alexa488 conjugated anti-mouse IgG, green signal) in combination with a polyclonal anti-HRSV goat immune serum (detected with Alexa568 conjugated anti-goat IgG, red signal) and DAPI nuclear stain (blue signal). Confocal images were recorded with a Zeiss SP5 confocal microscope. The scale bars in the right lower corner indicate 20 μ m. (E) Effect of HBc-SHeB immunization on body weight loss induced by HRSV B infection. Body weight determined on day 7 after challenge of individual mice relative to the body weight on the day of challenge is depicted. Horizontal bars represent the mean IgG titer. (F) Effect of HBc-SHeB immunization on HRSV B RNA levels in the BALF of infected mice. The graph represents the relative RNA level, calculated as $1/2^n$ (n is the number of PCR cycles) with the calculated value for the mock infected mice was set as 1. Horizontal bars represent the mean IgG titer. The relative levels of HRSV B N RNA were lower in mice immunized with HBc-SHeB than in mice immunized with HBc (significant difference between HBc and HBc-SHeB, $p = 0.0439$; Unpaired 2-sided Student-t test). Mouse 3 in the HBc-SHeB immunized group displayed the highest relative HRSV B N RNA level within this group and could be assigned as an outlier according to the Dixon's Q test. This mouse also displayed the largest loss in relative body weight and the highest infiltration of CD8⁺ T cells within this group. (G) Effect of HBc-SHeB immunization on pulmonary cell infiltration in infected mice. The cells in the BALF, isolated on day seven after infection, were counted manually. Horizontal bars represent the mean IgG titer. There was a trend towards less cell infiltration in the lungs of mice immunized with HBc-SHeB as compared to HBc immunized mice (significant difference between HBc and HBc-SHeB, $p = 0.0118$; Unpaired 2-sided Student-t test). (H) BALF immune cell composition was determined on an LSR-II flow cytometer (BD Biosciences) by analyzing cellular autofluorescence and surface expression of CD3 ϵ , CD4, CD8a, CD11b, CCR3, MHC-II, and CD11c. Seven days post infection, CD8⁺ T cells were the dominant leukocyte population in the BALF of all challenged mice. Horizontal bars represent the mean IgG titer. The percentage of CD8⁺ T cells in challenged HBc-SHeB immunized mice was significantly lower in HBC immunized mice (one-way ANOVA Tukey's multiple comparisons test). (I) SHeA peptide can only be recognized by SHeA immune serum and not by SHeB immune serum. The graph shows the O.D. 450 nm values of a SHeA peptide ELISA used to test the indicated dilutions of KLH serum, SHeA-KLH serum and HBc-SHeB serum. (J) SHeB peptide can only be recognized by SHeB immune serum and by not SHeA immune serum. The graph shows the O.D. 450 nm values of a SHeB peptide ELISA used to test the indicated dilutions of KLH serum, SHeA-KLH serum and HBc-SHeB serum.

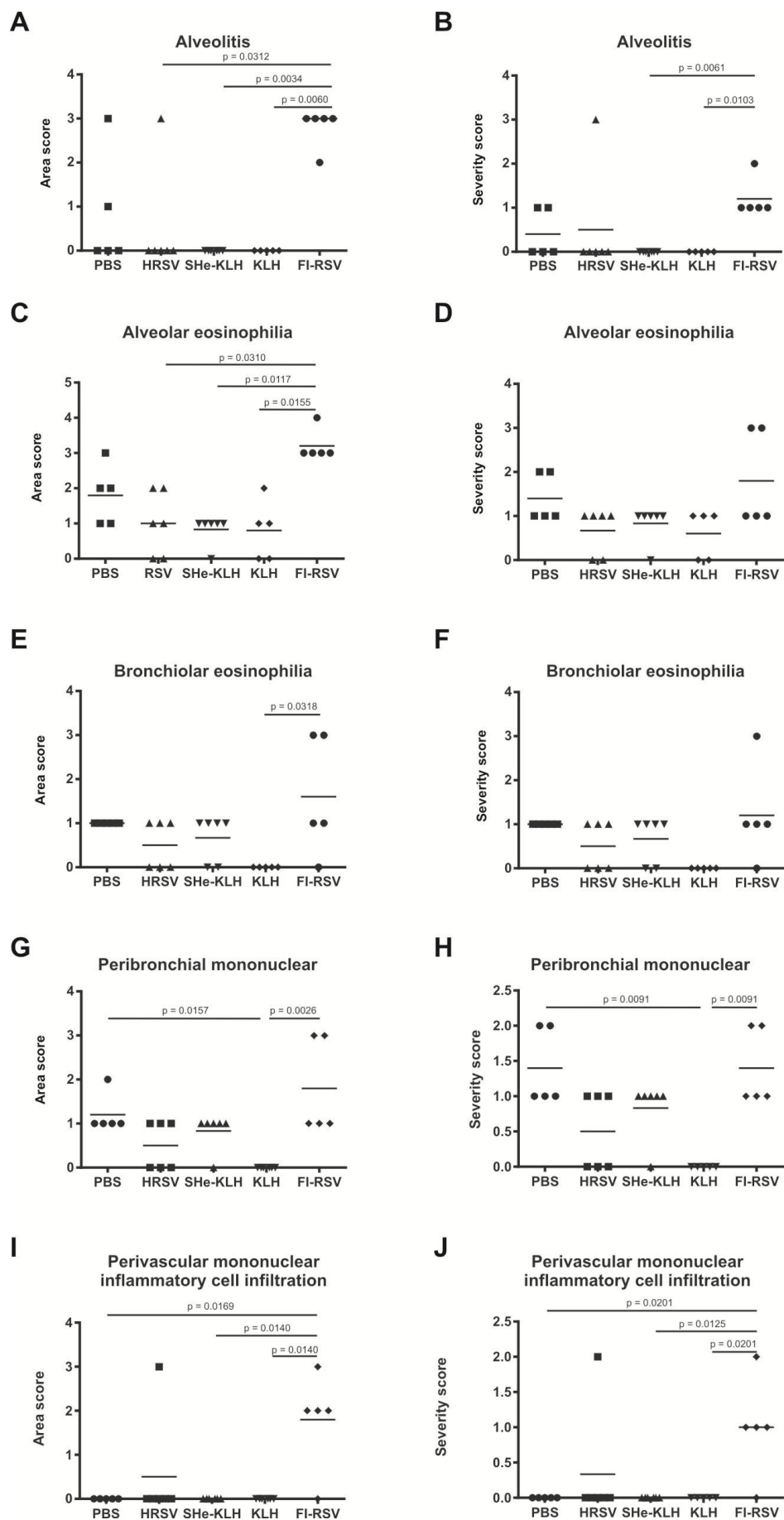

Figure S3. SHe-KLH immunization reduces pulmonary HRSV replication in cotton rats without exacerbating the disease. Histopathology score of cotton rats, immunized and challenged as in Figure 3. Five days post infection the left lung of each animal was prepared for histopathology. Sections were stained with hematoxylin & eosin and a pathologist from the Center for Comparative Medicine, BCM, scored them for the area and severity of the indicated parameters. The significance of the differences in scores was tested by one-way ANOVA Dunn`s multiple comparisons test. (A) Alveolitis area score. (B) Alveolitis severity score, (C) Alveolar eosinophilia area score. (D) Alveolar eosinophilia severity score. (E) Bronchiolar eosinophilia area score. (F) Bronchiolar eosinophilia area score. (G) Peribronchial mononuclear inflammatory cell infiltration area score. (H) Peribronchial mononuclear inflammatory cell infiltration severity score. (I) Perivascular mononuclear inflammatory cell infiltration area score. (J). Perivascular mononuclear inflammatory cell infiltration severity score. Each data point represents an individual cotton rat and horizontal bars depict the mean.

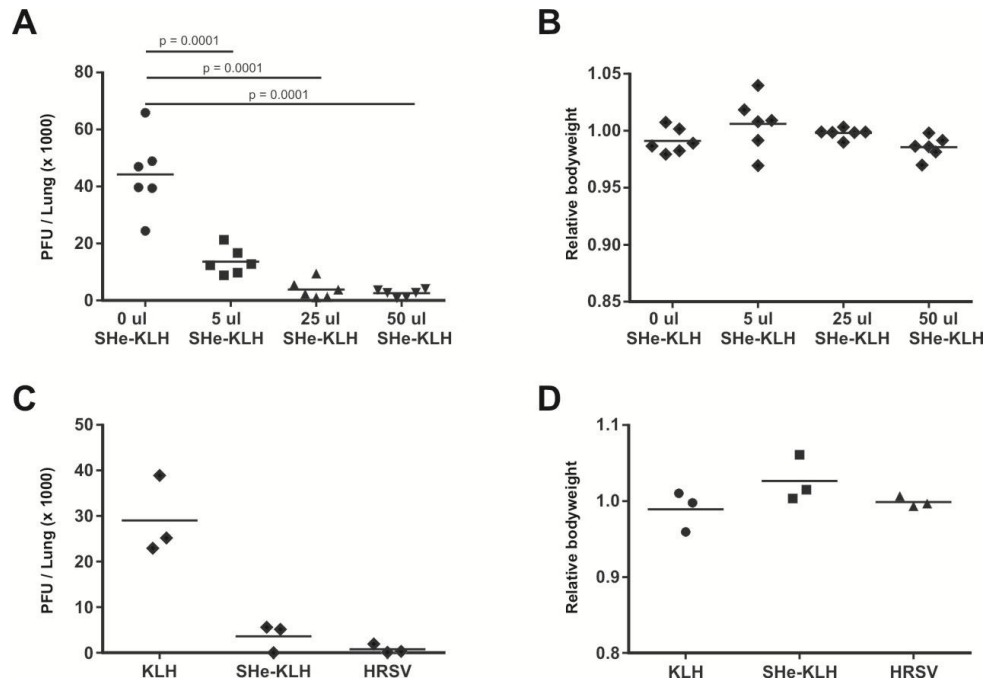

Figure S4. Passive immunization by intranasal and parenteral administration of SHe-KLH serum reduces HRSV replication. (A) The reduction of HRSV replication by SHe immune serum treatment is dose dependent. Four groups of 6 mice were treated one day before and one day after infection with respectively 50, 25, 5 and 0 μ l SHe-KLH immune serum, administered intranasally. All administrated sera were adjusted to a final volume of 50 μ l using KLH immune serum. Five days after challenge with 1×10^6 PFU RSV A2 the lungs were collected for HRSV titration. The graph shows the number of PFU per lung for each mouse and horizontal bars represent the mean (one-way ANOVA Tukey's multiple comparisons test). (B) Relative bodyweight of mice treated with different amounts of SHe-KLH immune serum at day 5 post infection calculated as the ratio between bodyweight at day 5 and bodyweight at day 0. Horizontal bars represent the mean. (C) Parental administration of SHe-KLH serum reduces HRSV replication. Sixteen hours before challenge with 5×10^5 PFU HRSV A2, 400 μ l of KLH or SHe-KLH immune serum or 300 μ l of convalescence serum of HRSV infected mice was administered intraperitoneally to BALB/c mice. Five days after challenge the lungs were collected to determine the pulmonary HRSV titer by plaque assay. The graph shows the number of PFU per lung for each mouse. Horizontal bars represent the mean. (D) Relative bodyweight of mice treated with the indicated sera via intraperitoneal injection at day 5 post infection calculated as the ratio between bodyweight at day 5 and bodyweight at day 0. Horizontal bars represent the mean.

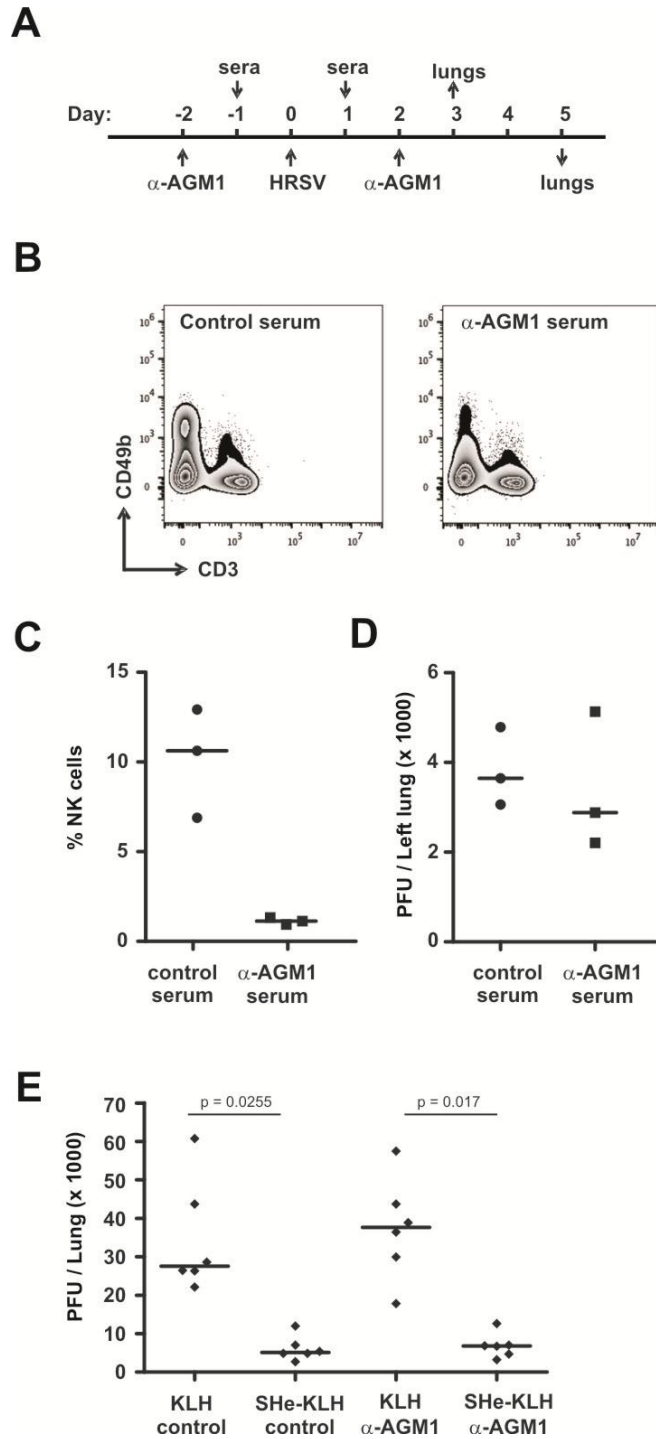

Figure S5. Depletion of NK cells does not abrogate the impact of SHe immune serum on HRSV replication. Six groups of BALB/c mice were either treated (intravenous injection) with 35 μ l anti-asialo GM1 rabbit serum (α -AGM1) or 35 μ l control rabbit serum (control serum) two days before and two day after infection. As indicated, mice were additionally treated intranasally with 40 μ l of mouse KLH and SHe-KLH immune serum one day before and one day after infection. All mice were infected with 1 x PFU 10^6 HRSV A2 on day 0. At 3 days post infection (peak of pulmonary NK cell infiltration) mice that were treated with either control rabbit serum or anti-asialoGM1 rabbit serum on days -2 and 2 were sacrificed to quantify the pulmonary HRSV titer by plaque assay (left lung) and NK cell infiltration by flow

cytometry (right lung lobes). These mice were not treated with KLH or SHe-KLH sera. (A) Schematic representation of the protocol used to investigate the impact of NK cell depletion on the anti-viral activity of SHe-KLH immune serum. (B and C) Mice treated with anti-asialoGM1 rabbit serum (α -AGM1 serum) display lowered NK cell infiltration as compared to mice treated with control rabbit serum (control serum). Single cells were prepared by passing minced right lung lobes through 70 μ m nylon meshes and investigated by flow cytometry. The lung cells were blocked with mouse Fc block (BD Pharmingen) and stained with LIVE/DEAD Fixable aqua LIVE/Dead stain, anti-F480-PerCP-Cy5.5, anti-CD3 ϵ -PE, anti-CD49b-V450. NK cells were defined as live lymphocytes, that were CD49b positive and negative for the F480 monocyte marker and the CD3 ϵ T cell marker. The left and right plots in B exemplify anti-CD3 ϵ and anti-CD19b staining of lung samples from mice that were treated with respectively control immune serum and anti-asialoGM1 serum. The graph in C represents the quantitative analysis of the presence of NK cells in the lungs of 3 mice that were treated with control serum and 3 mice that were treated with anti-asialoGM1 serum. (D) Treatment with anti-asialo GM1 serum did not significantly impact HRSV replication at 3 days post infection. (E) Treatment with anti-asialoGM1 does not impair SHe immune serum mediated reduction of HRSV replication. The graph shows the lung viral titers of each mouse that was treated with either, KLH immune serum in combination with rabbit control serum (KLH control), SHe-KLH immune serum in combination with rabbit control serum (SHe control), KLH immune serum in combination with rabbit anti-asialoGM1 serum (KLH α -AGM1) or SHe-KLH immune serum in combination with rabbit anti-asialogGM1 serum (SHe α -AGM1) (one-way ANOVA Dunn's multiple comparisons test). In C, D and E horizontal bars represent the median.

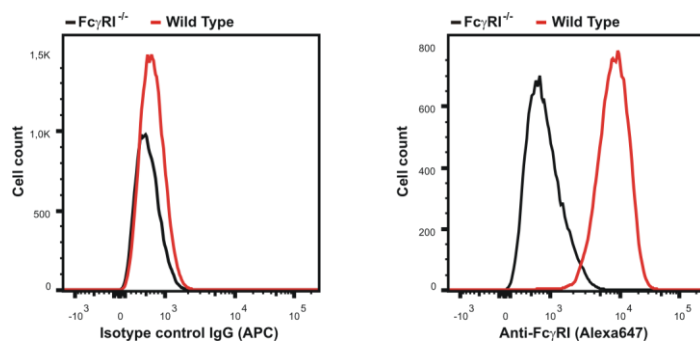

Figure S6. Alveolar macrophages express $Fc\gamma RI$ at their surface. The left and right hand histograms respectively show the binding of APC conjugated IgG1k isotype control mouse antibody and Alexa647 conjugated mouse IgG1k anti- $Fc\gamma RI$ antibody to resident alveolar macrophages in the BALF of wild type and $Fc\gamma R^{-/-}$ BALB/c mice. Resident alveolar macrophages were identified as autofluorescent (green channel) CD11c positive single cells.

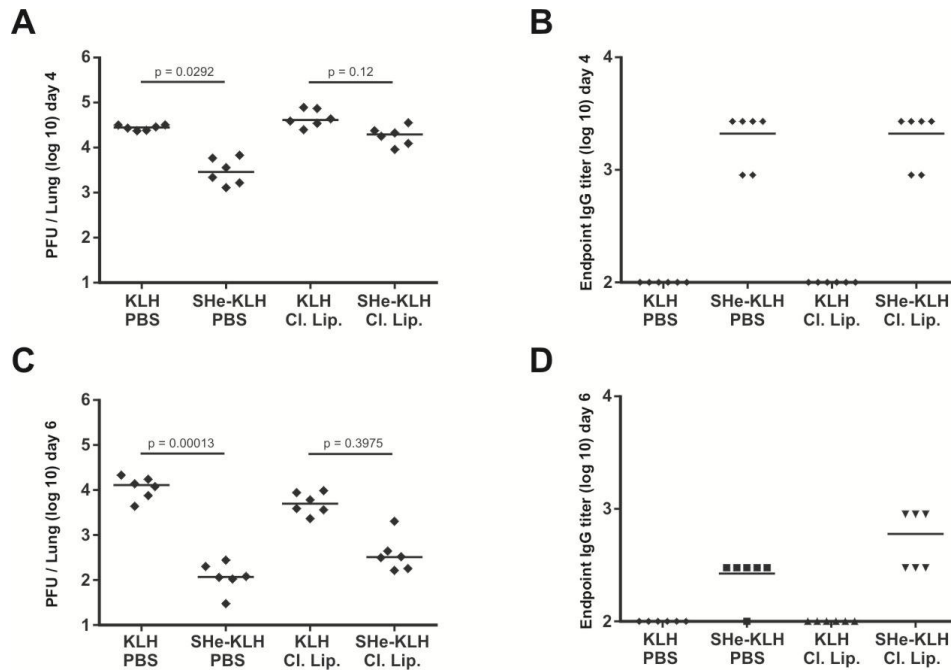

Figure S7. A role for alveolar macrophages SHe immune serum mediated reduction of viral replication at 4 and 6 days post infection. On day 3 before infection, PBS (PBS) or clodronate loaded liposomes (Cl. Lip.) were administered intranasally to BALB/c mice. One day before and one day after viral challenge, mice were additionally treated with 35 μ l of either KLH (KLH PBS and KLH cl. Lip.) or SHe-KLH (SHe-KLH PBS and SHe-KLH cl. Lip.) immune serum. At 4 and 6 days after infection with 1×10^6 PFU of HRSV A2, the lungs were collected for viral titration. (A) Depletion of alveolar macrophages abolishes SHe immune serum mediated reduction of HRSV replication at 4 days post infection. The graph shows the number of PFU per lung for each mouse, sampled at 4 days after challenge. Horizontal bars represent the median (one-way ANOVA Dunn's multiple comparisons test). (B) Depletion of alveolar macrophages does not impact the level of SHe-specific IgG in the day 4 lung homogenates of infected mice treated with SHe-KLH immune serum. The graph shows the endpoint SHe-specific IgG titer in the lung homogenates of each mouse as tested by SHe peptide ELISA with horizontal bars representing the mean. (C) Depletion of alveolar macrophages impacts SHe immune serum mediated reduction of HRSV replication at 6 days post infection. The graph shows the number of PFU per lung for each mouse, sampled at 6 days after challenge. Horizontal bars represent the median (one-way ANOVA Dunn's multiple comparisons test). (D) Depletion of alveolar macrophages does not lower the levels of SHe-specific IgG in the day 6 lung homogenates of infected mice treated with SHe-KLH immune serum. The graph shows the endpoint SHe-specific IgG titer in the lung homogenates of each mouse as tested by SHe peptide ELISA. Horizontal bars represent the mean IgG titer.

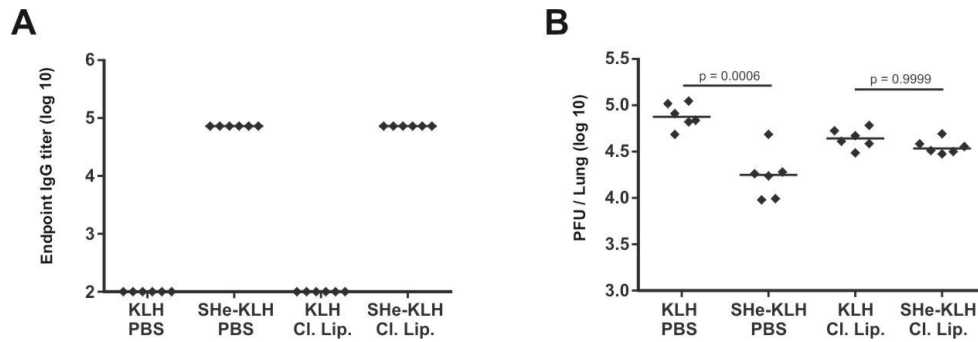

Figure S8. The reduction of HRSV replication by intraperitoneal treatment with SHe immune serum depends on alveolar macrophages. Three days before challenge BALB/c mice were treated intranasally with either PBS (PBS) or clodronate loaded liposomes (Cl. Lip.). One day before challenge 400ul of KLH (KLH PBS and KLH Cl. Lip.) or (SHe-KLH PBS and SHe-KLH Cl. Lip.) SHe-KLH immune serum was administered intraperitoneally. Five days after challenge with 1×10^6 PFU HRSV the lungs were collected for virus titration. (A) At 5 days post infection all mice that were passively immunized with SHe-KLH immune serum had comparable SHe-specific IgG titers in their sera. The graph shows the endpoint SHe-specific serum IgG titers at 5 days post infection, as determined by SHe peptide ELISA. Horizontal bars represent the mean (B) Depletion of alveolar macrophages impedes the reduction of HRSV replication evoked by parental administration of SHe immune sera. The graph shows the number of PFU per lung for each mouse and horizontal bars represent the median (one-way ANOVA Dunn's multiple comparisons test).

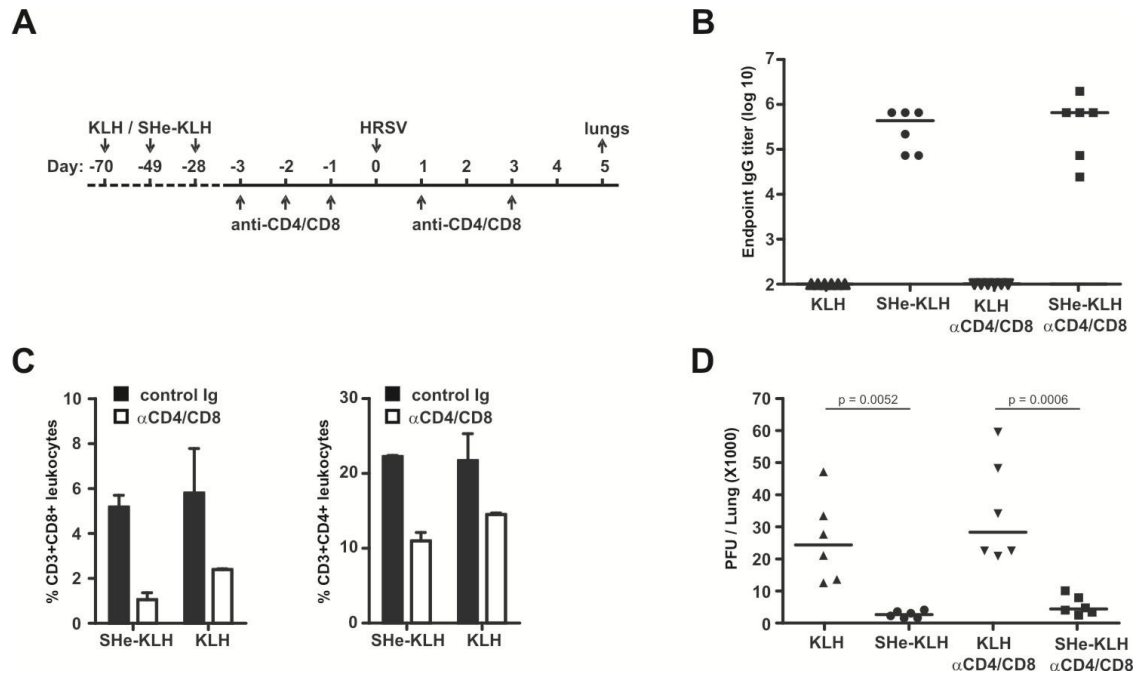

Figure S9. Depletion of CD4 and/or CD8 cells does not abrogate the reduction of HRSV replication in SHE immunized mice. BALB/c mice were immunized 3 times intraperitoneally with 20 µg KLH or 20 µg SHE-KLH (both in combination with incomplete Freund's adjuvant). Immunizations were performed at three-week intervals. Four weeks after the last immunization (day 0) the mice were challenged with 1x PFU 10⁶ HRSV A2. As indicated, mice were treated with 150 µg anti-CD4 (GT4) + 150 µg anti-CD8 (53-6-7) depleting rat IgG monoclonal antibodies or 300 µg control goat IgG at days -1, -2, -3, 1 and 3 by intraperitoneal injection. Five days after challenge the lungs and spleens were collected for analysis. (A) Schematic overview of the protocol used to investigate the role of CD4 and/or CD8 T cells. (B) SHE specific IgG endpoint titers 3 weeks after the second boost immunization detected by a SHE peptide ELISA. Horizontal bars represent the mean. (C) Treatment with anti-CD4 and anti-CD8 antibodies reduced the levels of CD4 and CD8 cells in the spleens. The left and right graph respectively show the percentage of CD3+CD8+ and CD3+CD4+ cells of the CD45+ spleen cells of mice treated with control rat IgG or anti-CD4 and anti-CD8 rat IgG. (D) Treatment with anti-CD4 and anti-CD8 antibodies does not abrogate the reduction in HRSV replication in mice immunized with SHE-KLH. The graph shows the lung viral titers at 5 days after infection and horizontal bars represent the median (one-way ANOVA Tukey's multiple comparisons test).

Figure S10. Sequence conservation of the SH ectodomain of HRSV A subgroup viruses. Alignment of the SHe amino acid sequences. (A) Alignment of SHe amino acid sequences of clinical HRSV A isolates collected by Lima *et al.* in Sao Paulo during the 2004 – 2005

season, including GA5 and GA2 genotypes (Lima *et al*, 2012). (B) Alignment of the SHe amino acid sequence of clinical HRSV A isolates collected in Denver Children's Hospital during the 2003-2004, 2004-2005, and 2005-2006 seasons by Kumaria *et al*. (Kumaria *et al*. 2011). (C) Alignment of the SHe amino acid sequences of clinical HRSV A isolates collected in the Milwaukee area from 1998 to 2010, by Rebuffo-Scheer *et al*. (Rebuffo-Scheer *et al*, 2011) including GA1, GA2, GA5 and GA7 genotypes. (D) Alignment of the SHe amino acid sequences of clinical HRSV A isolates collected in Belgium and the Netherlands between 2001 and 2011, by Tan *et al*. including GA1, GA2, GA4, GA5 and GA7 genotypes (Tan *et al*, 2013a and 2013b). E. Alignment of the SHe amino acid sequences of HRSV A lab strains: HRSV A2 (M74568); HRSV long (AY911262); RSS-2 (NC001803); HRSV A line-19 (FJ614813); HRSV Tracy.

gil157143655lgb|ABV24801.1|V42-65
gil157143669lgb|ABV24803.1|V42-65
gil157143661lgb|ABV24804.1|V42-65
gil157143669lgb|ABV24808.1|V42-65
gil157143671lgb|ABV24811.1|V42-65
gil157143679lgb|ABV24813.1|V42-65
gil157143687lgb|ABV24817.1|V42-65
gil157143693lgb|ABV24820.1|V42-65
gil157143695lgb|ABV24821.1|V42-65
gil157143697lgb|ABV24822.1|V42-65
gil157143709lgb|ABV24828.1|V42-65
gil157143719lgb|ABV24833.1|V42-65
gil157143727lgb|ABV24837.1|V42-65
gil157143733lgb|ABV24840.1|V42-65
gil157143737lgb|ABV24842.1|V42-65
gil157143739lgb|ABV24843.1|V42-65
gil157143731lgb|ABV24839.1|V42-65
gil157143729lgb|ABV24838.1|V42-65
gil157143741lgb|ABV24844.1|V42-65
gil157143683lgb|ABV24815.1|V42-65
gil157143703lgb|ABV24825.1|V42-65
gil157143725lgb|ABV24836.1|V42-65
gil157143647lgb|ABV24797.1|V42-65
gil157143649lgb|ABV24798.1|V42-65
gil157143651lgb|ABV24799.1|V42-65
gil157143653lgb|ABV24800.1|V42-65
gil157143657lgb|ABV24802.1|V42-65
gil157143663lgb|ABV24805.1|V42-65
gil157143667lgb|ABV24807.1|V42-65
gil157143671lgb|ABV24809.1|V42-65
gil157143673lgb|ABV24810.1|V42-65
gil157143677lgb|ABV24812.1|V42-65
gil157143681lgb|ABV24814.1|V42-65
gil157143685lgb|ABV24816.1|V42-65
gil157143689lgb|ABV24818.1|V42-65
gil157143691lgb|ABV24819.1|V42-65
gil157143699lgb|ABV24823.1|V42-65
gil157143701lgb|ABV24824.1|V42-65
gil157143705lgb|ABV24826.1|V42-65
gil157143707lgb|ABV24828.1|V42-65
gil157143711lgb|ABV24829.1|V42-65
gil157143713lgb|ABV24830.1|V42-65
gil157143715lgb|ABV24831.1|V42-65
gil157143717lgb|ABV24832.1|V42-65
gil157143721lgb|ABV24834.1|V42-65
gil157143723lgb|ABV24835.1|V42-65
gil157143645lgb|ABV24796.1|V42-65
gil157143735lgb|ABV24841.1|V42-65
gil157143665lgb|ABV24806.1|V42-65

[illegible]

gil352963102|glb|AEQ63584.1|/42-65
gil352963114|glb|AEQ63595.1|/42-65
gil352963126|glb|AEQ63606.1|/42-65
gil352963138|glb|AEQ63617.1|/42-65
gil352963150|glb|AEQ63628.1|/42-65
gil352963162|glb|AEQ63639.1|/42-65
gil352963174|glb|AEQ63650.1|/42-65
gil352963186|glb|AEQ63661.1|/42-65
gil352963198|glb|AEQ63672.1|/42-65

NKLS EHKTF CNKLTLEGGMYQINT

JX576729/42-65
JX576730/42-65
JX576731/42-65
JX576732/42-65
JX576733/42-65
JX576734/42-65
JX576735/42-65
JX576736/42-65
JX576737/42-65
JX576738/42-65
JX576739/42-65
JX576740/42-65
JX576741/42-65
JX576742/42-65
JX576743/42-65
JX576744/42-65
JX576745/42-65
JX576746/42-65
JX576747/42-65
JX576748/42-65
JX576749/42-65
JX576750/42-65
JX576751/42-65
JX576752/42-65
JX576753/42-65
JX576754/42-65
JX576755/42-65
JX576756/42-65
JX576757/42-65
JX576758/42-65
JX576759/42-65
JX576760/42-65
JX576761/42-65
JX576762/42-65

NKLSSEHKTFCKNTLEQQMQMYQINT

P

S S

P

gill11055541gib|AAG28139.1|I42-65
gill11055541gib|AAG28137.1|I42-65
gill11055521gib|AAG28127.1|I42-65
gill11055489gib|AAG28111.1|I42-65
gill11055497gib|AAG28115.1|I42-65
gill11055053gib|AAG28118.1|I42-65
gill11055483gib|AAG28108.1|I42-65
gill11055485gib|AAG28109.1|I42-65
gill11055487gib|AAG28110.1|I42-65
gill11054915gib|AAG28112.1|I42-65
gill11055493gib|AAG28113.1|I42-65
gill11055495gib|AAG28114.1|I42-65
gill11055499gib|AAG28116.1|I42-65
gill11055010gib|AAG28117.1|I42-65
gill11055050gib|AAG28119.1|I42-65
gill11055070gib|AAG28120.1|I42-65
gill11055090gib|AAG28121.1|I42-65
gill11055511gib|AAG28122.1|I42-65
gill11055513gib|AAG28123.1|I42-65
gill11055515gib|AAG28124.1|I42-65
gill11055517gib|AAG28125.1|I42-65
gill11055519gib|AAG28126.1|I42-65
gill11055523gib|AAG28128.1|I42-65
gill11055525gib|AAG28129.1|I42-65
gill11055527gib|AAG28130.1|I42-65
gill11055529gib|AAG28131.1|I42-65
gill11055531gib|AAG28132.1|I42-65
gill11055533gib|AAG28133.1|I42-65
gill11055535gib|AAG28134.1|I42-65
gill11055537gib|AAG28135.1|I42-65
gill11055539gib|AAG28136.1|I42-65
gill11055543gib|AAG28138.1|I42-65
gill11055547gib|AAG28140.1|I42-65
gill11055549gib|AAG28141.1|I42-65
gill11055551gib|AAG28142.1|I42-65
gill11055553gib|AAG28143.1|I42-65
gill11055555gib|AAG28144.1|I42-65
gill11055557gib|AAG28145.1|I42-65
gill11055559gib|AAG28146.1|I42-65
gill11055561gib|AAG28147.1|I42-65
gill11055563gib|AAG28148.1|I42-65
gill11055565gib|AAG28149.1|I42-65
gill11055567gib|AAG28150.1|I42-65
gill11055481gib|AAG28107.1|I42-65

Rebuffo-Scheer *et al.* (Rebuffo-Scheer *et al.*, 2011). (C) Alignment of the SHe amino acid sequences of clinical HRSV B isolates collected in Belgium and the Netherlands between 2001 and 2011 by Tan *et al.* (Tan *et al.*, 2013a and 2013b). (D) Alignment of the SHe amino acid sequences of HRSV B isolates reported by Chen *et al.* (Chen *et al.*, 2000).
